# Reconstructing the ancestral gene pool to uncover the origins and genetic links of Hmong–Mien speakers

**DOI:** 10.1186/s12915-024-01838-9

**Published:** 2024-03-13

**Authors:** Yang Gao, Xiaoxi Zhang, Hao Chen, Yan Lu, Sen Ma, Yajun Yang, Menghan Zhang, Shuhua Xu

**Affiliations:** 1 State Key Laboratory of Genetic Engineering, Human Phenome Institute, Zhangjiang Fudan International Innovation Center, Center for Evolutionary Biology, Department of Liver Surgery and Transplantation, Liver Cancer Institute, Zhongshan Hospital, Fudan University, Shanghai, 200032 China; 2 School of Life Science and Technology, ShanghaiTech University, Shanghai, 201210 China; 3 Key Laboratory of Computational Biology, Shanghai Institute of Nutrition and Health, University of Chinese Academy of Sciences, Chinese Academy of Sciences, Shanghai, 200031 China; 4 Ministry of Education Key Laboratory of Contemporary Anthropology, Collaborative Innovation Center for Genetics and Development, Fudan University, Shanghai, China; 5 Institute of Modern Languages and Linguistics, and Ministry of Education Key Laboratory of Contemporary Anthropology, School of Life Sciences, Fudan University, Shanghai, 200433 China

**Keywords:** Reconstructing genomes, Hmong-Mien, Next-generation sequencing, Genomic diversity, Local adaptation

## Abstract

**Background:**

Hmong–Mien (HM) speakers are linguistically related and live primarily in China, but little is known about their ancestral origins or the evolutionary mechanism shaping their genomic diversity. In particular, the lack of whole-genome sequencing data on the Yao population has prevented a full investigation of the origins and evolutionary history of HM speakers. As such, their origins are debatable.

**Results:**

Here, we made a deep sequencing effort of 80 Yao genomes, and our analysis together with 28 East Asian populations and 968 ancient Asian genomes suggested that there is a strong genetic basis for the formation of the HM language family. We estimated that the most recent common ancestor dates to 5800 years ago, while the genetic divergence between the HM and Tai–Kadai speakers was estimated to be 8200 years ago. We proposed that HM speakers originated from the Yangtze River Basin and spread with agricultural civilization. We identified highly differentiated variants between HM and Han Chinese, in particular, a deafness-related missense variant (rs72474224) in the *GJB2* gene is in a higher frequency in HM speakers than in others.

**Conclusions:**

Our results indicated complex gene flow and medically relevant variants involved in the HM speakers’ evolution history.

**Supplementary Information:**

The online version contains supplementary material available at 10.1186/s12915-024-01838-9.

## Main text

### Background

The Hmong–Mien (HM), also known as Miao–Yao, speakers are a group of linguistically related people living primarily in the mountains of Southern China and Southeast Asia. The majority of the HM speakers living in China include three main populations belonging to the Hmongic branch (Miao and She) and the Mienic branch (Yao) [1]. Previous studies on the genetics of HM populations mainly focus on the Y chromosome and mitochondrial DNA (mtDNA). The genetic difference between the Hmong and Mien populations has been found. The Hmong populations had more contact with the northern East Asians [2]. A specific and highly frequent Y haplotype group (O-M7) that existed in the modern HM population has been found in the ancient DNA samples from the Daxi site in the middle reach of the Yangtze River 5300–6400 years ago, which suggests that the Daxi people might share ancestry with modern HM populations [3]. However, the O-M7 haplotype also appeared in a group of Mon–Khmer speakers in the investigation of a larger sample [4]. The history of the HM population may not be simple. In the autosomal study, the HM population was found to carry a specific genetic component [5, 6] and receive gene flow from the ancestors of southern East Asians [7] and Sino–Tibetan-related ancestry [6]. However, including some other recent studies [8–11], genetic research in the HM population is mainly based on sparse single-nucleotide polymorphisms (SNPs), with the Miao population as the main population and the lack of Yao population data. Limited by data, the demographic history model and adaptive evolution of the HM populations are still not comprehensive and clear. At present, only one ancient individual (500 years old) related to the HM population has been discovered in Guangxi [12], which limits the study of HM history. Nonetheless, HM language populations still have been under-investigated in recent genomic studies using whole-genome sequencing [13, 14]; in particular, these studies only involve whole-genome sequencing data from the Miao and She populations but lack Yao population, which limits a full investigation of the ancestral origins and evolutionary history of HM speakers.

We collected 80 Yao blood samples from Guangxi Province, where more than half of the Yao people reside, and sequenced all of them to high coverage (30 ×) (see Additional file 1: Table S1) (see “Methods”). After quality control, 12.8 million high-quality variants were called autosomes, with more than 80% of them being biallelic single-nucleotide variants (SNVs) (see Additional file 2: Text S1). Through dbSNP (version 154) annotation, a total of 504,927 novel variants were discovered, accounting for 4.92% of the total number of biallelic SNVs. Further functional annotation indicated strong biological effects of 2889 novel variants. We also identified 4423 fragments of Yao with a unique or specific archaic infiltration with a total length of 71.56 MB using Archaicseeker2.0 [15] (Additional file 2: Text S2; Additional file 1: Tables S2-6). We integrate the Yao dataset with the public dataset of other populations (Additional file 2: Text S3, Fig. S1), such as Miao and She. Taking advantage of these datasets, we attempted to reveal the genetic structure of HM populations and provide genetic evidence for the origin of HM populations.

### Results

#### The genetic structure of the present-day HM population

**Fig. 1:**
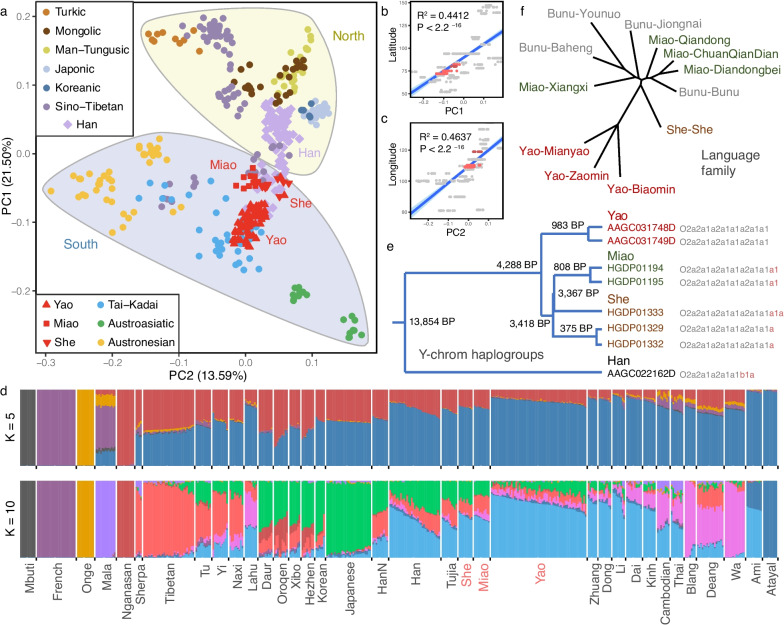
Genetic diversity among Hmong-Mien-speaking (HM) subpopulations. **a** The hierarchical genetic coordinates of HM speakers by PCA in the context of East Asia. The north–south population is divided by the geographical boundary, along the Qinling Mountains—Huai River line. **b** Linear fitting between PC1 and latitude. The red dots mean HM groups. The regression lines fitted using all the groups in the PCA. The blue shadow is a 95% confidence interval. **c** Linear fitting between PC2 and longitude. **d** The ancestral component inference of the HM speakers by ADMIXTURE software. **e** Paternal genetic model and divergence time estimation of HM population by Y chromosome O-M7 haplogroup. **f** The genealogy tree of the HM language family was constructed by the distance matrix generated by the comparison of basic words

All 80 Yao samples were collected from Guilin and Laibin. These two subgroups are closely clustered in the phylogenetic tree (Additional file 2: Fig. S2). The focus of this study is on the history of the HM population on a larger scale. Therefore, we regard two subgroups as a group in the analysis. To determine the genetic coordinates of present-day HM speakers, we performed principal component (PC) analysis (PCA) (see “Methods,” “PCA”). In the Eurasian context (Additional file 2: Fig. S3), three HM groups formed a cluster. HM speakers were located at the end of the East Asian cluster and far away from the European and Siberian clusters, indicating that HM speakers are typical East Asian descendants. In the context of East Asia (Fig. 1a), we applied linear fitting to project the genetic coordinates to the geographical coordinates of locations where population samples were collected. PC1 fitted the latitude well (Fig. 1b), while PC2 fitted the longitude well (Fig. 1c). Compared with the Han Chinese population, the HM speakers were located more in the south of East Asia and clustered together with Sino–Tibetan speakers and Tai–Kadai speakers. HM speakers were mainly distributed along the north–south axis. The Miao and She populations were located in the northern part of the HM language family, while the Yao population was located in a more southern part (Fig. 1a).

We next dissected the ancestry composition of HM speakers (see “Methods,” “ADMIXTURE”). When the number of ancestral populations (K) was assumed to be five, the results exhibited a low cross-validation error compared to others (Additional file 2: Fig. S4c). East Asian populations were mainly composed of the northern component, represented by Nganasan, and the southern component, represented by Taiwan Aboriginal. The Yao population harbored a higher southern genetic component than the Miao and She populations and the ancestral compositions of She and Miao were almost identical (Fig. 1d). These patterns are consistent with that shown in PCA (Fig. 1a). The analysis of shared genetic drift showed that Miao and She shared the most genetic drift (Additional file 2: Fig. S5) (see “Methods,” “Outgroup F3”). Therefore, the genetic evidence obtained so far supports that the split of Yao from other HM groups occurred earlier than that between She and Miao. We estimated that the divergence time between Yao and She was 5790 years by MSMC [16] and MSMC-IM [17], and we also estimated that the divergence time of Yao and Miao to be 5899 years and that of Miao and She was 7197 years (Additional file 2: Fig. S6a) (see “Methods,” “Divergence time”). These divergence patterns inferred with MSMC were not concordant with the genetic structure of the HM population. We speculate that the divergence time might have been affected due to population isolation or recent gene flow from peripheral populations.

Previous studies have shown that O2a2a1a (O-M7) is found in high frequency in some HM populations [18]. Daxi relics (5300–6400 years ago) in the middle reaches of the Yangtze River that carry this haplogroup are also considered to be related to the ancestors of modern HM populations [3]. Therefore, O-M7 can be considered as a specific Y haplogroup of HM speakers. In our analysis (see “Methods”), O-M7 was indeed enriched in the HM sub-branch groups (She: 3/7; Miao: 2/7; Yao: 2/44) but absent or rare in non-HM populations (Tibetan: 0/18; Dai: 0/6; Han: 1/21) (Additional file 1: Table S7-8). Eight O-M7 samples could be further classified into different subgroups (Fig. 1e). Unlike the Han Chinese population, the seven HM populations belonged to the O-N5 lineage. Consistent with the results obtained from the autosomal data, the evidence from the Y haplogroup analysis also supports the closer genetic relationship between Miao and She. Considering the specificity of O-N5 in the HM speakers, we estimated the time of the most recent common ancestor (MRCA) of this haplogroup in the HM speakers to be around 4288 years ago and that between Miao and She to be around 3418 years ago (Fig. 1e, Additional file 2: Fig. S7).

To further examine the relationship between genetic affinity and linguistic affinity, we calculated the distance between several branches of the HM language family using the similarity of basic words and by constructing the language tree based on the distance matrix (see “Methods”). The results show that the Miao and Yao language branches clustered respectively (Fig. 1f, Additional file 1: Table S9). Suffixes represent branches within a language family. Among them, the languages spoken by the Miao and Bunu populations belong to the Hmongic language branch of the HM language family, and the languages spoken by the Yao population belong to the Mienic language branch (Additional file 2: Text S4). The distance between the She and Miao languages was closer than that between the She and Yao languages, indicating that the Yao language split first, followed by the She and Miao languages. These patterns are highly consistent with the genetic data.

On a finer scale, we also examined the genetic diversity among the HM branches. Overall, the HM population showed lower genetic diversity than the Han Chinese and Tibetan populations belonging to the Sino–Tibetan language family (Additional file 2: Fig. S8). Among them, the genetic diversity of the Yao population was equivalent to that of Dai belonging to the Tai–Kadai language family, and higher than that of the She and Miao populations. The genetic diversity of the She population was the lowest among the three HM populations. From the historical changes in the effective population size, both She and Miao populations experienced a bottleneck event after the divergence of the HM populations (Additional file 2: Fig. S9), which may partly explain the differences in genetic diversity observed. We also found the sex-biased admixture in the HM population, where the admixture of HM populations was prone to combinations of southern males and northern females (Additional file 2: Fig. S10). Moreover, we did not observe HM-specific mtDNA haplogroups (Additional file 1: Table S10-11).

#### Admixture-driven differentiation of HM subpopulations

**Fig. 2:**
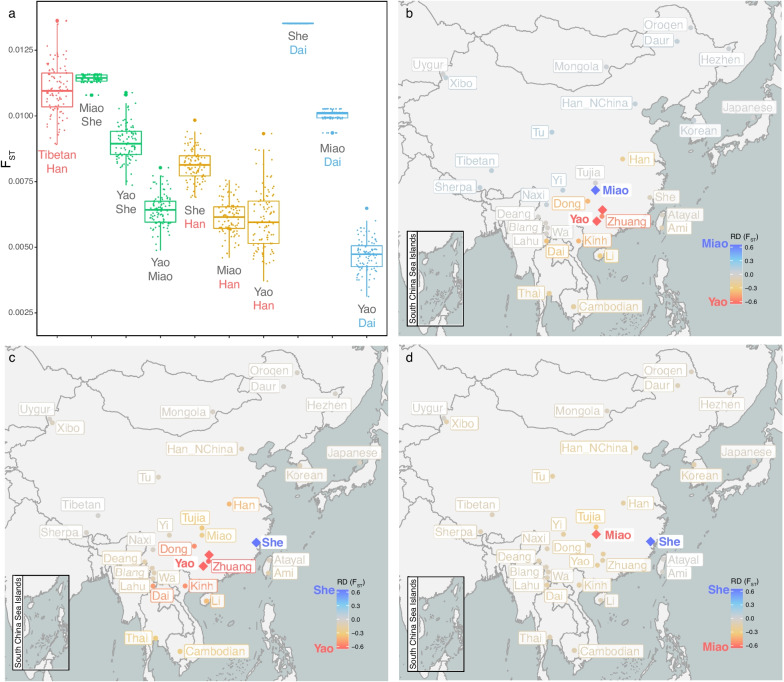
Gene flow from surrounding populations promoted the formation of a subpopulation structure within the HM population. **a** *F*_ST_ of the pairwise population was the result of randomly selecting 9 samples and repeating it 100 times. Each point represents a repetition. **b**–**d** The diamonds denote two target populations, and the other dots denote the populations tested. The Yao samples come from two sites, Guilin and Laibin. The color represents the relative difference in *F*_ST_ between the population to be tested and the two target populations (see “Methods”). The results show that surrounding populations contributed gene flow to the HM populations

Our further analysis indicated that gene flow from surrounding populations played an important role in shaping the genetic structure within the HM population. First, Yao showed a closer relationship with Tai–Kadai speakers (Fig. 2a, Additional file 1: Table S12), especially Zhuang, compared with Miao and She (Fig. 2b,c, Additional file 2: Fig. S5). PCA in the context of Southern East Asia (Additional file 2: Fig. S11) showed that Dong and Zhuang from the Tai–Kadai language family were the two populations with the closest relationship to Yao. These patterns were also confirmed by GLOBETROTTER [19] analysis, that is, the present-day Yao population was affected by gene flow from the ancestors of Zhuang, Han, and Miao by about 1100 years (Additional file 2: Text S5). The extra affinity between Yao and Zhuang may have resulted from the geographical overlapping of Yao and Zhuang, which can be seen from the sample distribution map (Fig. 2b&c). Second, Miao was more influenced by Tujia than She (Fig. 2d) and more influenced by northern populations than Yao (Fig. 2b), while She was relatively isolated and had fewer genetic connections with other populations (Fig. 2a,c,d). The isolation of the She population can also be confirmed with the analysis of the run of homozygosity (ROH) (Additional file 2: Fig. S12) and the rare allele sharing (Additional file 2: Text S6). This pattern can be largely explained by the geographical distribution of these populations (Fig. 2c,d). These results reflect recent genetic admixtures that occurred between the HM and surrounding populations. In particular, the three HM populations all showed a close relationship with the Han Chinese population. The genetic differentiation measured by the *F*_ST_ of each HM population with the Han population was even smaller than that of any pair of the HM populations (Fig. 2a), and they share quite a lot of rare variants with the Han Chinese population (Additional file 2: Text S6). The results of MSMC-IM also supported the recent gene flow between the three HM subpopulations and the Han population (Additional file 2: Fig. S6b).

#### Genetic origins of the HM population

To gain further insight into the genetic history of the present-day HM populations, we tried to explore the origin of the HM population. The main ancestral source of the HM speakers was the southern populations (> 70%) (Fig. 1d). In the higher dimension of admixture analysis (*K* = 10), the genetic components of East Asia were further subdivided. The southern component was divided into the southeast islands’ component (deep blue), represented by the Austronesian speakers; the southwest inland component (purplish-red), represented by the Austroasiatic speakers; and the South China components (blue), shared by the HM and the Tai–Kadai speakers. The proportion of the South China genetic composition was more than 50% in HM and Tai–Kadai speakers, and they were probably derived from the same ancestral population (Fig. 1d). We estimated the divergence time between the HM population and the Tai–Kadai population (see “Methods,” “Divergence time”). To minimize the influence of recent gene flow, we used the relatively isolated She population to represent HM speakers and estimated the divergence time between the She and Dai as 8200 years (Additional file 2: Fig. S6c). We also estimated that the divergence time between HM and Han was 10,800 years based on the historical change of effective population size (*N*_e_). Therefore, HM and Tai–Kadai have a closer genetic relationship compared with the Han Chinese population.

Furthermore, the South China component shared by HM and TK ancestors could also be observed in the Han Chinese population (Fig. 1d). The South China component showed a high proportion in the southern and northern Han Chinese populations (46.52%, 22.39%) but not in the Tibetan people of the Sino–Tibetan language family. These results suggest that the infiltration of the South China component into the Han Chinese population occurred after the Han-Tibetan divergence and before the expansion of the Han Chinese population. Through further analysis, we found that the Han Chinese population shared more genetic drift with HM populations than with Tai–Kadai populations (Additional file 1: Table S13). Therefore, the South China components in the Han Chinese population may have originated from the common ancestor of HM speakers.

**Fig. 3:**
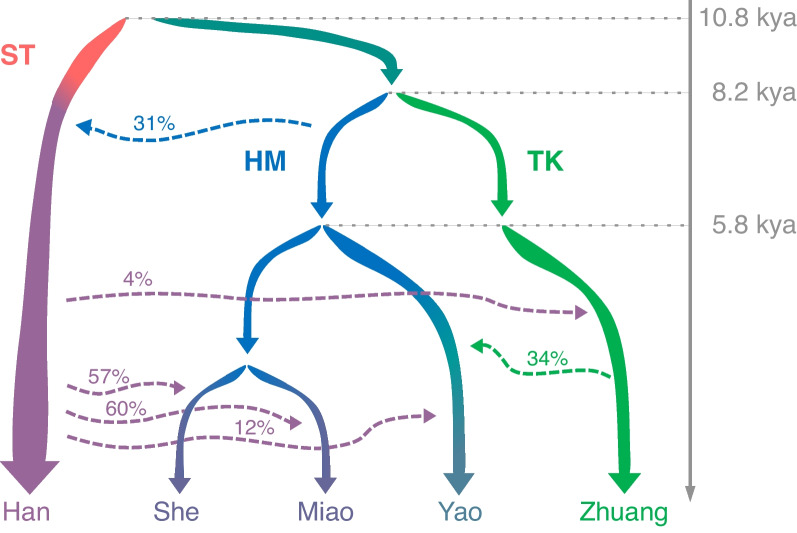
A fine origin history model of the HM speakers by qpGraph. The basic skeleton was obtained through the analysis of the above group structure and history and then further adjusted according to the score. The final score was 7.53 × 10^−6^. The dotted line represents the admixture events, and the percentage was the infiltration proportion of the current admixture event. The divergence time in the figure is the result of MSMC-IM analysis

We further built a fine evolution model of HM speakers with the qpGraph in ADMIXTOOLS2 [20] (see “Methods”). Based on the score of the model generated by the automatic search, we speculate that at least three genetic admixture events have occurred in the HM, TK, and ST populations (Additional file 2: Text S7). We designed the preliminary skeleton of the model based on our conclusion. According to the score, branch length, admixture ratio, and other information of alternative models (Additional file 2: Text S7, Fig. S13), the preliminary model was further fine-tuned, and finally, the software scored our model as 7.53 × 10^−6^, giving strong support to our model (Fig. 3, Additional file 2: Fig. S13b). According to the model, HM and Tai–Kadai populations shared the MRCA. About half of the genetic components of the early Han Chinese population came from the MRCA of the HM speakers, which may explain the substantial genetic differences between the Han and the Tibetan populations. The Yao was first separated from the HM speakers and was influenced by the Tai–Kadai speakers. Affected by the expansion of the Han Chinese population, the present-day HM populations independently received the gene flow from the Han Chinese populations of slightly different genetic backgrounds. Compared with Miao and Yao, She experienced a longer period of population isolation.

#### Reconstruction of HM ancestral genomes

**Fig. 4:**
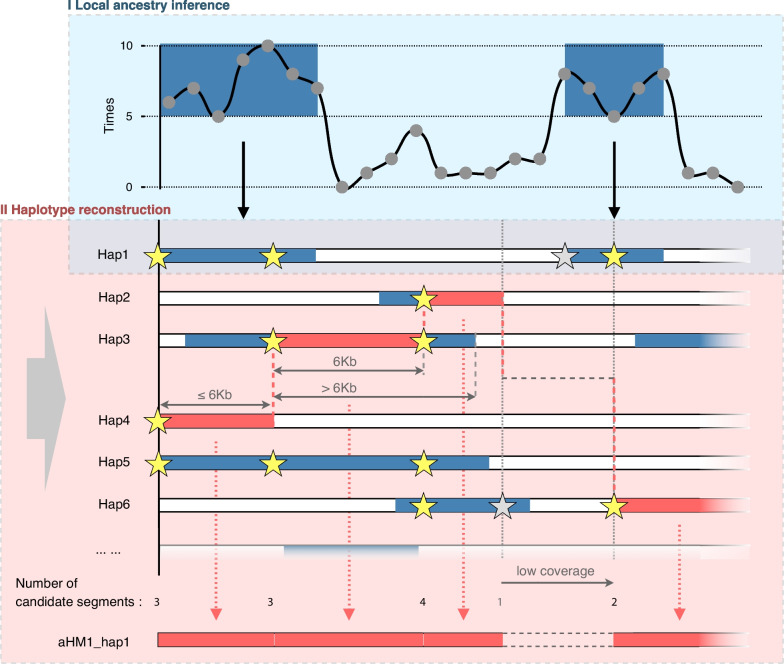
A workflow for reconstructing the ancestral genome based on present-day populations. I. Local ancestry inference of modern human genome. The segments inferred as HM in 5 of the 10 results were treated as candidate segments. Grey dots denote variants. II. Assembly of HM ancestral genomes using candidate segments. Each time, a segment was randomly selected from the optional starting sites and extended back no more than 6 kb. The low-coverage regions of the ancestral segments were skipped. The pentagrams denote the starting sites for candidates.

The ancestors of HM may have played an important role in the history of East Asia. However, the lack of sufficiently ancient HM-representative samples has limited a deeper analysis. In previous studies [5, 6], a special genetic component shared by HM populations was observed, but the corresponding local genomic regions were not identified. Here, we designed a scheme to extract ancestral fragments from the genomes of present-day HM populations for the reconstruction of the ancestral HM genomes (Fig. 4) (see “Methods”). We first identified the ancestral fragments in the genome of present-day HM populations using local ancestral inference [21] (see “Methods”). To dissect the recent linkage disequilibrium, all the obtained ancestral fragments were broken into segment sizes of 6 kb, and the ancestral genome was reconstructed by random sampling with replacement. In addition, to avoid the diversity bias in local regions resulting from uneven coverage of the genomic pool, we treated the genomic regions with low coverage of ancestral segments as missing data. Eventually, we constructed 30 high-fidelity HM ancestral (aHM) genomes using this method.

Next, we evaluated the quality of reconstructed aHM genomes. It turned out that the coverage of these reconstructed genomes was about 74.73% of the whole genome, a great gain compared with the traditional single-nucleotide polymorphism array and a typical ancient DNA sequencing effort. A significant positive correlation was observed between identity-by-descent (IBD) analysis and local ancestry inference [22] (Fisher’s exact test, *P* < 2.2^−16^) (Additional file 2: Text S8), indicating that the result of local ancestral inference is reliable (see “Methods”). The inbreeding analysis also showed that no pair of samples had a relationship of the third degree or closer in all the reconstructed ancestral genomes (see “Methods”). The reconstructed aHM genomes showed good quality. Analysis of shared genetic drift between the reconstructed ancestral genome and 29 modern East Asian populations [23] showed that the present-day Hmong-Mien populations and the reconstructed ancestral population shared the most genetic drift (Additional file 2: Fig. S14) (see “Methods,” “Outgroup F3”). Moreover, the results of ancestral inference show that the proportion of the genetic components of reconstructed aHM genomes in the ancient DNA samples (Gaohuahua, around 500 ya) [12] related to the HM population in Guangxi was higher than that in the present-day HM population (Additional file 2: Fig. S15). The ancient Gaohuahua genomes have been reported to share the most genetic drift with HM-speaking groups [12]. Thus, reconstructed aHM genomes largely represent the ancestors of HM speakers or proto-HM populations.

#### Ancient founders in the Yangtze River Basin

We further analyzed the genetic architecture of the proto-HM populations together with present-day populations. With the high coverage of aHM genomes constructed, we performed PCA of the aHM genomes with modern Eurasian populations (see “Methods”). The aHM showed distinction from the present-day HM populations on PC2, corresponding to a north–south geographical differentiation (Additional file 2: Fig. S16). The geographic coordinates of the aHM were more southeast than those of the other present-day East Asian populations. This pattern can be explained by the persistent influence of northern East Asian ancestry in southern East Asia [24].

Next, we performed an ADMIXTURE analysis of combined data of the aHM and present-day populations assuming a different number of ancestral populations (*K* = 2–15) (Additional file 2: Fig. S17) (see “Methods”). Interestingly, the aHM showed a single pure genetic component throughout all of the ADMIXTURE analyses. Assuming three ancestral populations (*K* = 3), our reconstructed aHM genomes represented a genetic component of Southern East Asia. When *K* = 6, this genetic component of aHM was in a low frequency among all of the East Asian populations, suggesting that the aHM population may be one of the founder ancestral groups of East Asians. In addition, the aHM component showed a higher frequency in southern populations than in northern populations, and even slightly higher in the present-day HM populations, the Tai–Kadai populations, the Taiwanese aboriginal people, and the Han Chinese population. These results suggest that the aHM population likely originated in the southeast region of East Asia.

**Fig. 5:**
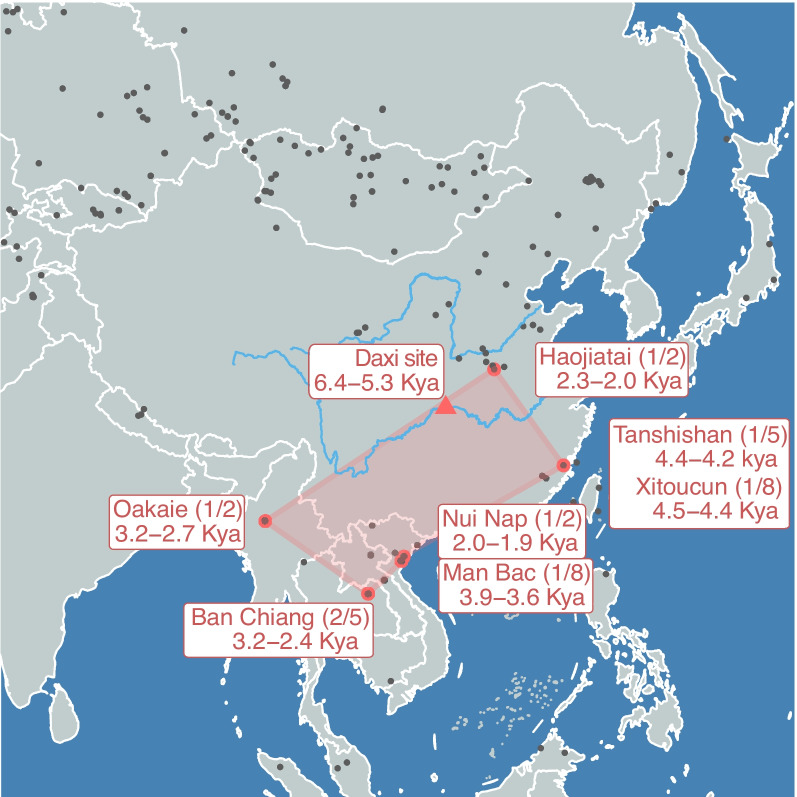
A map for tracking the population migration history of the HM population. The black dots in the image are all publicly available ancient DNA samples. The red triangle denotes the Daxi site in the middle reaches of the Yangtze River. The red dot shows the eight ancient DNA samples that share the most genetic drift with the reconstructed aHM population. The numbers in parentheses represent the total number of samples in the relic and the number of samples associated with aHM. These ancient DNA samples came from people who mainly lived in southern East Asia for about 3000–4000 years, which is later than the time of the most recent common ancestor of present-day HM populations. These ancient DNA samples reflect the footprints of population diffusion after the divergence of HM populations. Ancient DNA samples showed that the ancestors of the HM population reached the China–Indochina Peninsula in the South and the Yellow River Basin in the North

In contrast to the ancient DNA samples of known geographical location but unknown ethnic information, our reconstructed aHM genomes provided a more explicit message in tracing human migration history. We integrated 29 East Asian populations with the reconstructed HM ancestral population as a reference dataset as well as 968 ancient DNA samples located in East, Southeast, and Central Asia [24–38]. By analyzing the shared genetic drift of each ancient sample and each reference population [23], we identified eight high-quality ancient samples that shared the most genetic drift with the aHM genomes (Fig. 5, Additional file 1: Tables S14-15) (see “Methods”). These samples mainly lived about 3000–4000 years ago, that is, the period after the divergence of HM populations. The geographical locations of these ancient samples indicated the habitation of the aHM population and the dispersal routes since the initial divergence. In brief, the aHM population was mainly distributed in the south of East Asia but later arrived in Thailand in the South and the lower reaches of the Yellow River Basin in the north. The aHM population was likely to live between the two places, that is, in the middle and lower reaches of the Yangtze River Basin. A study based on the Y chromosome in Daxi Culture also identified traces of HM ancestors’ activities in the Yangtze River Basin [3]. According to the dating of eight ancient DNA samples, the earliest sample appeared in the southeast coastal area of China around 4300–4400 years ago. The ancient DNA samples found in the China–Indochina Peninsula and the Yellow River Basin also provided evidence that the aHM population spread in East Asia to both directions, north and south.

#### Footprints of natural selection in the HM population

**Fig. 6:**
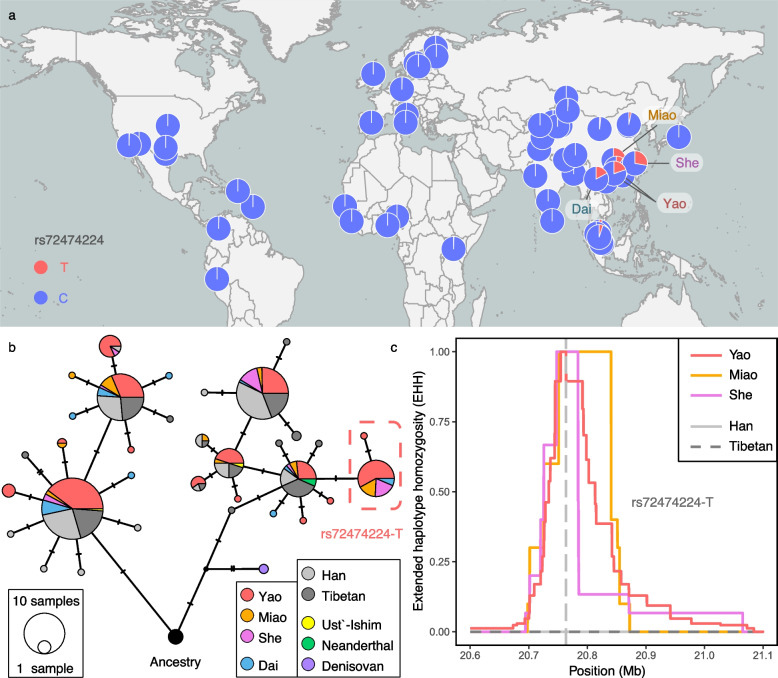
rs72474224 showed a specific positive selection signal in populations of Southern East Asia, including HM. **a** Distribution of allele frequency of rs72474224-T in the context of worldwide populations. **b** Haplotype network was constructed by PopART using the 39 SNVs located in *GJB2*. The rs72474224-T only appears on the haplotypes in the red box. **c** Extended haplotype homozygosity (EHH) of rs72474224-T. This allele showed positive selection signals in HM populations

We separately extracted 10 samples from the Yao, Miao, and She populations to construct the HM population group, and calculated *F*_ST_ with the Han population. A total of 13,180 variants (Additional file 1: Table S16, Additional file 2: Fig. S18) were identified based on between-population analysis using site-specific *F*_ST_ (top 0.1%), of which 35.30% were expression quantitative trait loci (eQTL), which is much higher than the average density of eQTL in the whole genome (15.47%) (*P* < 2.2 × 10^−16^; Additional file 2: Text S8). There were 1854 genes covered by the 13,180 variants significantly enriched in multiple regulatory pathways and the composition of protein structure such as protein binding (FDR *P* = 2.5 × 10^−58^), plasma membrane (FDR *P* = 7.2 × 10^−36^), and obesity-related traits (FDR *P* = 5.1 × 10^−22^) (Additional file 1: Table S17). In addition, we also found that some variants associated with disease risk were different among populations. For example, rs7756992, a variant in the *CDKAL1* gene, associated with type-2 diabetes [39], had a lower frequency in the Yao population (Yao-G:0.492; Han-G:0.625), which means that the risk of disease might be reduced. Notably, a deafness-related missense mutation rs72474224 (p.Val37 Ile) located in the *GJB2* gene showed a higher allele frequency in the southern East Asian population including HM [39] (Yao-T:0.153; Miao-T:0.250; She-T:0.278) but a lower frequency in other populations in the world (Fig. 6a) [40]. The variant rs72474224 is highly conservative (GERP: 5.21); the derived allele is located only on a specific haplotype from Southern East Asian populations (Fig. 6b). It cannot be explained by the archaic infiltration or the founder effect (Fig. 6b). In addition, rs72474224 showed a strong selection signal in HM populations (Fig. 6c). This prevalence of the disease-risk allele in natural populations suggests the existence of pleiotropy.

To overcome the power loss in detecting natural selection due to the potential cancellation of signatures resulting from recent gene flow, we further analyzed population-specific ancestral fragments based on the reconstructed ancestral genomes. We calculated the *F*_ST_ of each variant between the reconstructed aHM and the Han Chinese population (Additional file 1: Table S18). Despite there being some missing variants in the reconstructed ancestral genomes, we successfully collected a total of about 10 million SNVs shared by the ancestral and present-day populations. It turned out that this strategy increased the power for detection (Additional file 2: Fig. S19). Altogether 2371 (37.39%) of a total of 6341 extra SNVs underlying natural selection were identified as significant eQTL in the GTEx database, which is again higher than the average density of eQTL in the whole genome (15.47%). The genes covered by these additional variants were also found to be significantly enriched in multiple functional pathways such as protein binding (FDR *P* = 1.5 × 10^−33^) and plasma membrane (FDR *P* = 1.6 × 10^−26^) (Additional file 1: Table S19). The most significantly differentiated variants (*F*_ST:_ 0.853) were located in the *AF146191.4* gene (lincRNA) on chromosome 4. Another significantly differentiated variant was located in the *PLCB2* gene on chromosome 15 (*F*_ST:_ 0.847), which encodes a phosphodiesterase and participates in the signal transduction pathway of the type 2 taste receptor [41].

We also used the iHS method to identify the selection signals of the HM population. We analyzed the present-day HM population (consisting of 10 Yao, 10 Miao, and 10 She) (Additional file 2: Fig. S20a) and the reconstructed aHM population (Additional file 2: Fig. S20b) separately, and detected possible selection variants of 133,611 (Additional file 1: Table S20) and 146,604 (Additional file 1: Table S21) according to the threshold of | iHS |> 2. The intersection variants of the two populations are 9,950. In the present-day HM population, the most significant signal gene is *LINC00552*, a lncRNA. Other genes include *ADI1*, *HLA-DQA1*, and *SFTPA1*. In the aHM population, the most significant signal gene is *RBFOX2.* Other genes include *DDX1*, *SLC4A8*, *SMARCC1,* and *CHD9*. We also compared the selection signals found by *F*_ST_ and iHS methods, but the signal variants found by different methods have differences and few intersections (Additional file 2: Fig. S21).

We also applied four methods, *F*_ST_ (Additional file 2: Fig. S22), iHS (Additional file 2: Fig. S23), XPEHH (Additional file 2: Fig. S24), and Tajima’s D, to detect the natural selection signals of Yao population, and determined candidate selection signal regions based on 100 kb segmentation. For the results, most of the signal segments identified in each method were unique on their own, and only a small number of signals were shared with other methods (Additional file 2: Fig. S25). Thus, we found 6 sharing segments in the 3 most commonly used methods (*F*_ST_, iHS, and XPEHH). These 6 shared signal segments included chr13:99400001–99800000 (Two protein-coding genes: *SLC15A1*, plays an important role in the uptake and digestion of dietary proteins [42]; *DOCK9*, associated with irregular astigmatism and corneal ectasia [43]), chr14:106000001–106100000 (Two protein-coding genes: *IGHA2* and *IGHG4*, both of them involve immunoglobulin heavy chains [44, 45]) and chr19:57500001–57600000 (No protein-coding gene. Only a pseudogene *RPL7AP69*). Furthermore, using a more relaxed threshold can help us find two signal segments sharing in all four methods including chr1:161500001–161600000 (Two protein-coding genes: *FCGR3A* and *FCGR3B*, both of them involve low affinity immunoglobulin gamma Fc region receptor [46, 47]) and chr6:32500001–32600000 (Two protein-coding genes: *HLA-DRB1* and *HLA-DQA1*, both of them involve HLA class II histocompatibility antigen [48]).

Taking advantage of the new approach based on the reconstructed ancestral genomes, we applied more stringent criteria for screening the natural selection signals: (i) candidate adaptive alleles only exist in HM populations; (ii) statistically significant difference in allele frequency (> 0.3) between the aHM and the Han Chinese population; (iii) significant enrichment of adaptive alleles in the aHM genomes (see “Methods”). We identified 2779 variants (Additional file 1: Table S22), 395 of which were eQTL. Notably, the 175 genes regulated by these QTL were significantly enriched in the external side of the plasma membrane (FDR *P* = 1.7 × 10^−13^), interferon-gamma-mediated signaling pathway (FDR *P* = 8.2 × 10^−12^), ER to Golgi transport vesicle membrane (FDR *P* = 1.7 × 10^−11^), and MHC class II protein complex (FDR *P* = 4.8 × 10^−10^) (Additional file 1: Table S23). In addition, among the 2779 variants, we identified a differential variant aggregation region of 102 kb, and 151 variants in this region showed a frequency difference of 70% or larger. The two genes involved in this region, *SLCO1B3*, and *SLCO1B7*, are members of the liver-specific organic anion transporter family; they encode transmembrane receptors, play a key role in bile acid and bilirubin transport, and participate in bile salt recycling [49–51].

**Fig. 7:**
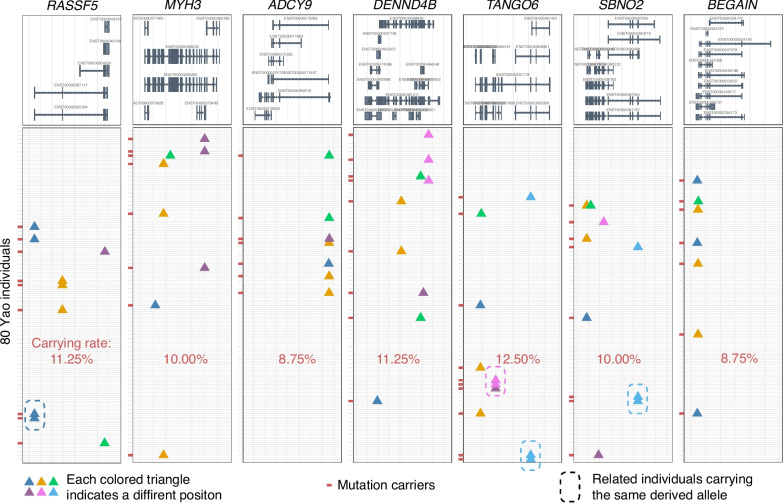
Rare derived alleles with strong effects were dispersed in different individuals. The top shows all transcripts of each gene, and each line below is a Yao individual. Triangles of different colors indicate that rare derived alleles are located in different loci. The box indicates that two related individuals share the same rare variants at this locus. The individual marked with a short red line indicates that the individual carries at least one variant with strong effects on the gene. The proportion of individuals carrying strong effect variants on each gene is the carrying rate

We also found that the rare variants with strong effects played a role in the adaptation of the Yao population to the environment. Based on the allele frequency, we determined the ancestral allele in the East Asian population and then found new mutations in the Yao population. Seven protein-coding genes (*RASSF5*, *MYH3*, *ADCY9*, *DENND48*, *TANGO6*, *SBNO2*, and *BEGAIN*) were specifically enriched with strong effects on recently derived alleles in the Yao population (Additional file 1: Table S24). However, these alleles were not detected in the Han population. We annotated 42 rare variants specifically carried by the Yao individuals on these 7 genes for conservatism (GERP) and pathogenicity (CADD). We found 32 out of the 42 mutations with GREP ≥ 2 or CADD ≥ 10, especially 27 of them with GREP ≥ 4 or CADD ≥ 15, indicating the potential functions of these rare mutations. We also found that these strong effects in rare variants showed familial aggregation, that is, 4 of the 24 pairs of related samples shared at least one strong effect in rare variants in these genes. However, this proportion was only 35/3136 in the unrelated sample pairs (*P* = 1.76 × 10^−4^; Additional file 2: Text S8, Additional file 1: Table S25). The enrichment of rare mutations in these genes suggests that there may be positive selection at the gene level in the Yao population. Multiple rare variants with a strong effect from one gene were dispersed in different individuals (Fig. 7), which greatly improves the carrying rate of mutated genes in the Yao population. However, the positive selection acting on these genes did not increase the frequency of each allele, and they were missing in the Han Chinese population. Therefore, we speculate that there might be negative selection forces as well acting on these sites. The functional annotation also indicated the potential pleiotropy of these genes. For example, *SBNO2* is related to bone homeostasis [52] and also participates in the pro-inflammatory cascade [53]. *MYH3* is related to muscle organ development [54] and also to the Freeman-Sheldon syndrome [55]. *RASSF5* is related to wound healing [56] and also to multiple human cancers [57–61]. The various effects of these pleiotropic genes could be driven by different evolutionary forces.

In addition, we identified HLA types in the HM population (Additional file 1: Table S26). The HLA-B * 15:02 allele has been reported to be strongly associated with severe skin allergic reactions caused by carbamazepine, and the East Asian population has a high allele frequency [62]. In our data, the frequency of the HLA-B * 15:02 allele in the Yao population (14.52%, 18/124) is higher than that in Miao (2/20), She (0/20), Dai (1/18), Han (2/80), and Tibetan (0/76) populations (Additional file 2: Fig. S26, Additional file 1: Table S27). This guides the use of related drugs. However, the driving force of HLA-B * 15:02 allele fluctuation in the population needs to be studied.

## Discussion

In this study, we collected 80 samples of the Yao population and performed a deep whole-genome sequence. We identified 504,927 novel variants and 71.56 MB of Yao-specific ancient infiltration fragments which expanded the gene pool of modern humans. In particular, we have also found mutations such as rs72474224 ((p.Val37Ile, *GJB2*) that are associated with deafness in the HM population are in high frequency (Fig. 6), indicating the medical potential of genetic research on minority populations. Although the sample size may lead to a rough estimation of the allele frequency in the Miao and She populations, the high allele frequency observed in three independent populations is sufficient to support our conclusion.

We inferred the paternal and maternal haplogroups of the HM population, as well as estimated the time to the MRCA of the HM-specific paternal haplogroup O-N5 (4288 years ago, Fig. 1e), which is approximately 1800 years earlier than the time of the MRCA of O-N5 previously reported (2500 years ago) [7]. This may benefit from the whole-genome deep sequencing. In addition, we found that in the three populations of Yao, Miao, and She, the main paternal and maternal haplogroups were different (Additional file 1: Table S8, S11), which may reflect the complex population history after the divergence of the HM population. In addition, the frequency of O-M7 (upstream of O-N5) in the ancient population of the Daxi site is 5/16, which is similar to the frequency in the present-day She (3/7) and Miao (2/7) populations, but the frequency of O-M7 in the Yao population is extremely low (2/44). Daxi site may be a direct ancestor of the Miao population or the O-M7 lineage may have been diluted in the Yao population. However, the lack of autosomal variation at the Daxi site makes it difficult to draw an exclusive conclusion.

Our analyses including PCA (Fig. 1a), ancestry (Fig. 1d), linguistic similarity (Fig. 1f), and Y chromosome haplogroups (Fig. 1e) showed that Yao was first separated from the HM population. However, we also noticed that the *F*_ST_ between Miao and She is larger than that between them and Yao (Fig. 2a). We speculate that *F*_ST_ is more sensitive to recent population isolation. For example, Ami and Atayal, two indigenous peoples in Taiwan Island, have extremely high *F*_ST_ with other populations (Additional file 1: Table S12). Therefore, we evaluated the recent gene flow between the HM subgroups and the surrounding populations with *F*_ST_. In addition, we found that there were differences in the divergence time or the time of the MRCA obtained based on different data and methods. Y chromosome data estimates that the MRCA of the HM population is 4288 years ago (Fig. 1e), while MSMC and MSMC-IM estimate that the divergence time between Yao and Miao, as well as between Yao and She, is 5800 years ago, and the divergence time between Miao and She is 7197 years ago (Additional file 2: Fig. S6a). The genetic traces on the Y chromosome can be disrupted by recent population events, such as the bottleneck leading to the loss of ancient haplogroups, and recent gene flow leading to the insertion of new haplogroups, all of which may lead to underestimation or overestimation of the time of the MRCA. Therefore, the time estimation from the Y chromosome is only used as auxiliary evidence. The divergence pattern of MSMC is consistent with the results of *F*_ST_, and we speculate that it may also be affected by recent gene flow or isolation, such as the admixture between HM and Han resulting in underestimated divergence time (Additional file 2: Fig. S6b). In the MSMC results, the divergence time between Yao and She is consistent with that between Yao and Miao, which is consistent with the model where Yao first separated from the HM population. Therefore, we speculate that this is a reliable time for the MRCA for the HM population. As for the divergence time between Miao and She, which is inferred to be 7197 years ago, we speculate that this may be related to admixture with the Han Chinese population and recent isolation.

Our further analysis of genetic and linguistic data supported the consistent relationship within the HM group, which indicated a substantial genetic basis of the HM language family. In particular, the She population was found to have closer ties with the Miao population from the perspective of both genetics and linguistics. At present, the two views on the formation time of the HM language family are 2500 years ago [63] and 4243 years ago [64]. The time of 4243 years is similar to the time of the MRCA of the HM population inferred based on the Y chromosome (4288 years ago), but later than the time of the MRCA of HM speakers inferred based on the autosome. (5800 years ago). In addition, from this perspective, the formation time of the Hmong (Miao) language branch is 2777 years ago [64], which is later than our inferred time based on the Y chromosome (3418 years ago). Therefore, we suggested that the formation time of the HM language family was underestimated owing to the population admixture after divergence. We also observed considerable gene flow to the HM population from the Tai–Kadai and the Han populations, implying a complex population history of different language speakers.

Based on the comparison of the ancestral components of autosomes and X-chromosomes, we found that there is a sex-biased admixture in present-day HM populations, with more admixture between southern males and northern females (Additional file 2: Fig. S10). However, this is not contradictory to the common belief that the population migration southward was male-driven. The main reason is that the sex bias of population migration is different from the preference for spouse selection. Some ethnic historical records indicate that most southern ethnic minorities did not intermarry with outsiders until nearly a hundred years ago, as can be seen from the ancestral components on the autosomes. In the past, there was a tradition among the Yao ethnic group that women did not marry other ethnic groups, while a minority would marry women from other ethnic groups, which may lead to a high proportion of northern ancestral components on the X chromosome.

In palaeontological research, the ancestral genome has been reconstructed by searching the homologous blocks of the extant genome under the divergence model [65, 66]. However, this method is not suitable for reconstructing the ancestors of closely related human populations in a complex model with admixture due to recombination and replacement. We thus developed an approach to reconstruct the ancestral genomes of a certain ethnic group based on present-day population genome data. This strategy facilitates the identification of the ancestral genomic segments from the present-day populations and overcomes the limitations of ancient DNA data or the unavailability of ethnic information from ancient samples. It can show the genomic diversity of ancestral populations. Moreover, we found that the method is robust. In most genomic regions, there is no significant sequence difference between different populations, while a small number of ancestral inference errors in these regions are unlikely to affect the results significantly. However, we would make it clear that the reconstructed ancestral genomes may not represent the true individuals that have existed in real history in the current version; rather, they are the proxy genomes representing an ancient gene pool of relatively isolated populations with less influence from recent gene flow owing to the massive migration of human populations.

With this approach, we gained further insights into the genetic origins and admixture history of the HM populations. The Yangtze River Basin is one of the cradles of agricultural civilization in East Asia. There is evidence that rice agriculture has been developing in the lower reaches of the Yangtze River for 6000–8000 years. We estimated that the differentiation of the HM populations was about 5800 years, which is also consistent with the origin and development time of agricultural civilization in the Yangtze River Basin. There is also evidence of the establishment of rice agriculture in the middle reaches of the Yangtze River during the Daxi period about 5300–6400 years ago. Ancient DNA samples found in the Daxi site indicated that the Daxi culture is related to the HM ancestral population. We also found evidence that the HM ancestors lived in the Yangtze River Basin and extended northward and southward through the study of other ancient DNA samples. The high consistency between geographical and genetic coordinates implied that the HM population has settled in the current area for a long time (Fig. 1b,c). All the evidence suggests that the ancestral HM population was one of the early groups that developed with agricultural civilization in the Yangtze River Basin. The genetic components of the HM ancestral population found in present-day East Asian populations, especially southern East Asian populations, also support the founder effect of HM ancestors.

In this study, we also revealed an admixture pattern of “mutual gene flow” among several major populations in East Asia, including the Han Chinese, HM, and Tai–Kadai populations. Our model is different from that proposed in previous studies, which typically assumes that two or more ancestral populations are admixed into a new population (A + B—> C). In our model, the two ancestral populations have a short contract or a small proportion of infiltration such that the two ancestral populations obtain a certain proportion of genetic components from each other (A + B—> A' + B'). According to our current research, this admixture pattern of mutual infiltration may have played a major role in the formation of most East Asian populations.

Despite there being more published ancient DNA data available, there is an obvious gap between ancient samples and present-day populations owing to the lack of ethnic label in the ancient samples. Frequent human dispersals and prevalent gene flow have prevented most studies from establishing reliable links between geographic information and ethnic information. In this study, we attempted to label 968 ancient DNA samples by calculating the shared genetic drift between each sample and present-day populations. This effort narrowed the gap to some degree and established a rough link between ancient samples and present-day populations, which is expected to facilitate tracing the genetic origins and admixture history of present-day populations.

We use multiple methods to detect potential natural selection signals on the genome of HM and Yao populations, and different methods complement each other based on different principles and assumptions (Additional file 2: Fig. S21, S25). Although the significance of some selected signals is currently unclear, it provides a reference for other related studies. In addition, we also found that the reconstructed aHM genome is very helpful for detecting selection signals. It can weaken the impact of recent gene flow, highlighting some masked signal variants. However, from the results, it can be seen that the selection signals based on reconstructed ancestral genome detection are more complementary than substitutive compared to using present-day population genomes. We speculate that this may reflect the natural selection in different historical periods.

We found that the cancellation of natural selection signatures could result in a power loss of commonly used methods for detecting selection. Several such cases have been identified in the analysis of Yao genome data. The cancellation could be due to multiple effects of a gene being affected by positive and negative selection forces at a certain time point, or it could be due to environmental changes in history that have caused a gene to be successively affected by positive and negative selection forces at different time points. This type of alleles might be in relatively low frequency in the population and do not show remarkable differentiation between different populations. These atypical selection signals of the low allele frequency and cancellation of natural selection can be only recognized when population data are available and carefully analyzed. Therefore, we suggest that much more attention be paid to the low-frequency variants in the analysis of diverse natural populations, especially ethnic minority groups.

## Conclusions

In this study, we investigated the genetic diversity and local adaptation of the HM populations. We developed a method for reconstructing the ancestral genome based on genomes of present-day populations, and we further demonstrated that this method is robust and helpful for the study of genetic structure, population history, and local adaptation. Our results support that the three main HM populations, Miao, Yao, and She, shared the most recent common ancestor about 5800 years ago, with their origins dating back to the middle reaches of the Yangtze River. The genetic history of the HM populations is complex. The Yao population first diverged from the HM populations. The She population has experienced a longer period of population isolation after separating from the Miao population. All three HM groups have been influenced by gene flow from the surrounding populations. For example, Yao received gene flow from Zhuang, while Miao received gene flow from Tujia. Each of the three HM groups received a slightly different gene flow from the Han Chinese population. In the study of local adaptation, we found that a risk variant rs72474224 in the *GJB2* gene is associated with deafness and underlying positive selection in the HM populations. We speculate that this gene may have other unknown effects on the evolution of the HM populations.

### Methods

#### Samples

In total, 80 Yao blood samples were collected from the main inhabited area of the Yao population, Guangxi Province, in southern China. Informed consent was obtained from all the individuals who participated in this study.

### Data compilation

**Table:** 

Resource	Source	Identifier
**Deposited data**		
Yao80	This manuscript	xushua@fudan.edu.cn
AAGC	[67]	https://ngdc.cncb.ac.cn/bioproject/browse/PRJCA000246
HGDP	[68]	https://www.ebi.ac.uk/ena/browser/view/PRJEB6463
SGDP	[14]	https://www.ebi.ac.uk/ena/browser/view/PRJEB9586
1KGP	[69]	https://www.internationalgenome.org/
Human origin	[70]	http://www.ebi.ac.uk/ena/data/view/PRJEB6272
ADP	[71]	https://ega-archive.org/datasets/EGAD00010001491
GTEx v7		https://gtexportal.org/
Clinvar	[39]	https://www.ncbi.nlm.nih.gov/clinvar/
*PGG*.SNV	[40]	https://www.pggsnv.org/
**Software and algorithms**		
bwa	[72]	https://github.com/lh3/bwa
picard	[73]	http://broadinstitute.github.io/picard/
samtools	[74]	https://github.com/samtools/samtools
GATK	[75]	https://gatk.broadinstitute.org/hc/en-us
bcftools	[74]	https://github.com/samtools/bcftools
king	[76]	https://www.chen.kingrelatedness.com
vcftools	[77]	https://github.com/vcftools/vcftools
Archaicseeker2	[15]	https://github.com/Shuhua-Group/ArchaicSeeker2.0
FlashPCA2	[78]	https://github.com/gabraham/flashpca
ADMIXTURE	[79]	http://software.genetics.ucla.edu/admixture/
AdmixTools	[23]	https://github.com/DReichLab/AdmixTools/
AdmixTools 2	[20]	https://github.com/uqrmaie1/admixtools/
HaploGrep	[80]	https://github.com/seppinho/haplogrep-cmd
Y-LineageTracker	[81]	https://github.com/Shuhua-Group/Y-LineageTracker
BEAST	[82]	http://beast.community/
SHAPEIT2	[83]	https://mathgen.stats.ox.ac.uk/genetics_software/shapeit/shapeit.html
ChromoPainter	[21]	https://github.com/sahwa/ChromoPainterV2
GLOBETROTTER	[19]	https://people.maths.bris.ac.uk/~madjl/finestructure/globetrotter.html
hap-ibd	[22]	https://github.com/browning-lab/hap-ibd
MSMC	[16]	https://github.com/stschiff/msmc
MSMC-IM	[17]	https://github.com/wangke16/MSMC-IM
VEP	[84]	https://github.com/Ensembl/ensembl-vep
Selscan	[85]	https://github.com/szpiech/selscan
KOBAS	[86]	http://kobas.cbi.pku.edu.cn/
PopART	[87]	http://popart.otago.ac.nz/index.shtml
REHH	[88]	https://gitlab.com/oneoverx/rehh/
Data analysis code	This manuscript	https://github.com/Shuhua-Group/Construct-ancestral-genome 10.5281/zenodo.10499683

#### Whole-genome sequencing and data processing

Each 1–3 µg of DNA from blood was sheared into segments of 200–800 bp with the Covaris system. DNA segments were then treated according to the Illumina DNA sample preparation protocol. The DNA library was sequenced on the Illumina X10 platform using 150 bp paired-end reads, and the sequencing depth of clean data exceeded 30 × for each sample.

Reads of each sample were merged and mapped to the human reference genome (b37) using bwa mem version 0.7.10. The Picard (version 1.117) was used to mark duplicate reads after mapping. Then, we executed local indel realignment and base quality recalibration using GATK (version 3.6). Variant calling was executed through the *haplotypecaller* command of GATK, using the gvcf mode. The whole-genome sequencing data of other reference populations used in this study adopted the same analysis process, including 9 Dai, 10 Miao, and 10 She from the Human Genome Diversity Project (HGDP) [13] and Simons Genomic Diversity Project (SGDP) [14] and 40 Han, 33 Tibetan, and 5 Sherpa from the Asian Admixed Genomes Consortium (AAGC) [67].

We integrated these 80 Yao samples and other reference populations mentioned above by a joint call and implemented strict quality control through VQSR and a universal mask. Finally, autosomal biallelic SNVs were retained for downstream analysis (Panel 1). For the Y chromosome and mtDNA, the output of joint variant calling was used directly without VQSR and a universal mask. To further expand the reference populations, we integrated Panel 1 with global populations from the Affymetrix Human Origins genotyping dataset [70] and the East Asian population from the Asian Diversity Project (ADP) [71] by retaining the common variants, which were used in the analysis of variant density insensitivity (Panel 2).

#### Genetic relationship

We calculated the genetic relationship of all Yao samples from Panel 1 using the KING (version 2.2, related model) and removed at least one sample for each related pair to ensure that no pair had a relationship of the third degree or closer after filtering.

For 30 reconstructed genomes of the HM ancestral population, we performed the relationship inference, and the results show that no pair had a relationship of the third degree or closer.

#### PCA

PCA was performed in three different contexts, including Eurasia, East Asia, and Southern East Asia context. First, target populations were extracted from the Panel 2 dataset using the bcftools [74] (version 1.10.2) for each context, and the variants with a missing rate ≤ 1% and minor allele frequency ≥ 5% were retained. These informative candidate variants were downsampled by vcftools [77] (version 0.1.15) according to the principle that the physical distance between any two variants is not less than 50 kb, which can eliminate the linkage disequilibrium (LD) between loci. Finally, 33,576 (Eurasia contexts), 32,443 (East Asia contexts), and 32,308 (southern East Asia) SNPs were used in PCA. The selected variants underwent PCA by FlashPCA [78] (version 2.0). In addition, in the results of the East Asian context, we performed linear fitting of PC1 and PC2 with the latitude and longitude, respectively.

When performing PCA on the reconstructed ancestral population, to balance the sample size, we randomly selected 10 reconstructed samples and integrated them with the Panel 2 dataset in the Eurasian context. Considering the missing rate of reconstructed ancestral genomes, we filtered out the variants with a missing rate greater than 1% in any population. Then, as in the previous process, the variants with a minor allele frequency < 5% in all samples were filtered out and downsampled according to a 50-kb physical interval for LD. Finally, 24,740 SNPs were used. FlashPCA was used to perform PCA.

#### ADMIXTURE

We used the Panel 2 dataset. The selected target population includes all East Asian people and Mbuti is the representative of African ancestry, with French as the representative of European ancestral, Mala as the representative of South Asian ancestral, Onge as the representative of Negrito, and Nganasan as the representative of Siberian ancestral. The data preprocessing was similar to PCA. The variants with a missing rate ≤ 1% and a minor allele frequency ≥ 5% were retained, but the standard of downsampling was a 10-kb interval, which helped to improve the resolution. Finally, 73,785 SNPs were used. We ran the ADMIXTURE [79] software according to the gradient of the assumed number of ancestral populations from 2 to 15. Then, we ran an additional 20 times to detect fluctuations in the admixture model. Only one-time fluctuation occurs at *K* = 5, and other Admixture models are consistent (Fig. S4).

When inferring the ancestral components of the reconstructed HM ancestral population with the present-day populations, we randomly selected 10 of the reconstructed 30 ancestral genomes and integrated them with samples used in the above ADMIXTURE analysis. The only difference from the above data preprocessing is that only variants with a missing rate = 0 were retained. In total, 32,956 SNPs were used.

#### Pairwise FST

To quantify the genetic affinity between Yao and other populations more accurately, we used the Panel 1 dataset, 13,198,356 SNPs in total. To avoid the bias caused by the sample size, according to the sample size of the population with the smallest sample size, we randomly selected nine samples from each population each time and calculated the pairwise *F*_ST_ between two populations 100 times [89].

To research the recent population admixture, we used the Panel 2 dataset to infer the degree to of the two target populations were affected by other populations after the divergence of the two target populations. Here, we developed a new method. We calculated the relative difference (RD) of genomic affinity between the two target populations (A, B) and other reference populations (X). The formula is as follows:

$$relative\;difference=\frac{F_{ST}(A,X)-F_{ST}(B,X)}{F_{ST}\left(A,X\right)+F_{ST}(B,X)}$$

If the relative difference is positive, the greater it is, the more the genetic communication between population X and population B. If the relative difference is negative, the smaller it is, the more the genetic communication between population X and population A.

The variants from the Panel 2 dataset with a missing rate ≤ 1% and a minor allele frequency ≥ 10% were retained and downsampled according to a 10-kb physical interval. In total, 66,640 SNPs were used to calculate *F*_ST_.

#### Outgroup F3

To infer the genetic relationship between the three present-day HM populations (Yao, Miao, and She) and other East Asian populations, we calculated the shared genetic drift between each HM population and all other populations with the outgroup F3 method. Based on the Panel 2 dataset, we filtered out the variants with a missing rate > 1% in all samples or MAF < 5% in all populations and downsampled according to the physical interval of no less than 10 kb between any two variants to eliminate LD. After data processing, 79,802 SNPs are reserved. The outgroup F3 was performed using the qp3pop command in the ADMIXTOOLS software package [23] with Mbuti as the outgroup population.

When evaluating the quality of the reconstructed HM ancestral genomes, we extracted 10 reconstructed HM ancestral samples and merged them with the Panel2 dataset. Only the variants without any missing genotype in all samples and MAF more than 10% in any single population were retained, and the variants were downsampled using a 10-kb physical interval. Finally, 36,361 SNPs are reserved. We calculated the shared genetic drift of each HM-related population including three present-day HM populations (Yao, Miao, She) and reconstructed ancestral populations (aHM) with other East Asian reference populations using the outgroup F3 method.

To identify the ancient DNA samples most closely related to aHM, we labeled these ancient samples according to the classification of present-day populations. We integrated 968 publicly available ancient samples, using the Panel 2 dataset and reconstructed aHM data as the reference populations, and filtered out variants with a missing rate > 20% in any one reference population. Finally, 106,153 SNPs are reserved. However, due to the high missing rate, the number of observable sites of ancient DNA samples ranges from several hundred to several hundred thousand. We calculated the shared genetic drift of each ancient sample with all reference populations. We found that 11 ancient samples shared the most genetic drift with the reconstructed aHM compared to other reference populations. Among them, three samples were filtered out owing to low coverage, and finally, only eight ancient samples were retained for further analysis.

#### Y chromosome and mtDNA haplogroups

To construct the maternal and paternal genealogy, we classified the mtDNA and Y chromosome haplogroups of the Panel1 datasets. The mtDNA haplogroups were classified using HaploGrep2 [80] based on PhyloTree17 [90]. The Y chromosome haplogroups were classified using Y-LineageTracker [81] based on the ISOGG Y-DNA phylogenetic tree 2019–2020 (https://isogg.org).

To estimate the age of NRY haplogroups, 106 samples with sufficient coverage and depth were used to construct the NRY phylogenetic tree and calculate the age of haplogroups. We performed Bayesian evolutionary analyses using BEAST v.2.6.0 [82] with the GTR model under the strict clock to construct the phylogenetic tree and estimate the coalescent times of NRY haplogroups and their sub-lineages. The results are visualized in FigTree v1.4.4. The age of the NRY haplogroup CT-M168 (71,760 years, 95% CI = 69,777–73,799)[91] was used for the age estimation of all NRY haplogroups.

#### Linguistic distance

The linguistic distance matrix among the 12 HM languages was derived from Deng’s work [92]. For calculating the linguistic distance, we first obtained the similarity matrix of HM languages, which is measured by the sharing proportions of lexical cognates between every two language samples. Second, we transformed the similarity matrix into the distance matrix using the following equation:

$$D=(-{{\text{log}}}_{10}(s))\times 100$$

where *D* is the distance value between two languages, and *s* is the similarity value between two languages.

#### qpGraph

To visualize the population history model of present-day HM populations and verify the reliability of our model, we constructed an admixture graph using qpGraph in the admixtools2 package [20]. Here, we used the Panel 2 dataset and filtered out variants with a missing rate > 0 in all samples and an MAF < 0.1 in all populations. Finally, 75,381 SNPs are reserved. According to the results of the above analyses, we designed the basic skeleton of the HM population’s origin model and refined the model based on the information provided by admixtools2 such as the score, branch length, and admixture ratio.

#### Haplotype inference

To determine the haplotypes of each sample, we filtered out all the variants with a missing rate greater than 5% and used SHAPEIT2 [83] to phase all the samples from Panel 1. We used a genetic map obtained from HapMap II [93] and adopted 0.5 Mb for the window size, as recommended for sequence data.

#### Local ancestry inference

To identify the HM-specific ancestral segments in the genome of present-day HM populations, we performed local ancestral inference by ChromoPainter [21] (V2). Based on the phased data of the Panel 1 dataset, we used 9 Dai, 9 Han, 9 Tibetan, and 9 present-day HM samples as the donors to paint the other HM samples. Considering the divergent order of the HM populations and the recent population admixture, we used nine Yao samples to represent the HM population as the donor when we painted the genomes of the Miao and She populations, and we used nine She samples to represent the HM population as the donor when we painted the Yao samples. According to the default parameters, each genome was painted 10 times. These results were used to reconstruct the aHM genome.

#### Reconstruction of ancestral genomes

Firstly, based on local ancestral inference, we constructed a candidate haplotype library for HM ancestors. For sample size balance, there are 10 samples each for Miao, Yao, and She in the library. To fully reflect the genetic diversity of the population, sample selection has comprehensively considered the results of IBD and PCA. Candidate haplotypes in the library require support from at least half of the 10 ancestral inference results. Assembly starts from a starting site and randomly selects one from the candidate haplotypes passing through this site. Then extend back along this haplotype for no more than 6000 bp. When a breakpoint was encountered or the expansion reached 6000 bp, we repeat the above steps to randomly select the next candidate haplotype to continue extending (sampling without replacement). To avoid the diversity deviation caused by the low coverage of ancestral fragments in local areas, we only selected genome regions covered by at least 12 candidate haplotypes (20% of the sample size in the library) to reconstruct the ancestral genome. Finally, we reconstructed 60 aHM haplotypes and assembled 30 aHM individual genomes. Assembly of each genome is through replacement sampling.

#### IBD

We adopted the default parameters of hap-ibd software for IBD analysis based on the phased Panel 1 dataset with the HapMap II genetic map. We compared the HM ancestral fragment from local ancestry inference and a homologous fragment from IBD analysis at the haplotype level for 30 HM samples used in the reconstruction of ancestral genomes. We designed the contingency table (Additional file 2: Text S9) and tested whether there was a significant correlation between the above two fragments through Fisher’s exact test.

#### Divergence time

We applied multiple sequentially Markovian coalescent (MSMC, version 1.1.0) analyses [16] to infer the divergence time from the high-coverage genomes. Two samples were selected from each population when the divergence time was to be estimated for the two populations. We first used bamCaller.py for each sample to generate the mask file, which gives the regions in which the genome of that individual was covered sufficiently. In addition to the mask for each sample, we also used the additional mappability mask for GRCh37, which gives all regions in which short sequencing reads can be uniquely mapped. Rather than phasing the data from bamCaller.py directly, we used the phased Panel 1 dataset. We calculated the absolute estimation by assuming a slow mutation rate of ~ 1.25 × 10^−8^ per base per human generation for a generation time of 29 years, as the results based on the slow mutation rate agree better with the palaeoanthropological record and with the estimates from mtDNA [94–96]. Then, further analysis was conducted according to the recommended parameters of MSMC-IM (-beta 1e-8,1e-6 –printfittingdetails –plotfittingdetails –xlog). The divergence time between Han and HM was estimated by their Ne inferred by MSMC. We calculated the mean value and standard deviation of Ne for each period of three HM populations. If Han’s Ne was not within the range of adding or subtracting three times the standard deviation of HM in the same period, Han and HM were considered divergent. We chose the earliest time range of their latest divergence and calculated the median of this time range as the final divergence time.

#### Selection

To identify variants underlying natural selection in HM populations, we used the Han Chinese population as the reference population. The loci with a missing rate of more than 10% in any of the five groups were filtered out, including 40 Han, 10 She, 10 Miao, and 62 unrelated Yao samples from two sampling sites. We calculated the *F*_ST_ of each locus using 40 Han samples and 30 modern HM populations (10 Yao, 10 Miao, and 10 She) used in reconstructing the ancestral genome, and the algorithm balanced the difference in sample size [89]. The calculated results were sorted from large to small according to *F*_ST_, and the loci with a large *F*_ST_ (top 0.1%) were considered to be affected by natural selection. These loci were annotated by the Ensembl Variant Effect Predictor (VEP) [97] (GRCh37 ensemble92), the GTEx database v7 (https://gtexportal.org/), and the Clinvar database [39] (GRCh37.20210302). Functional enrichment analysis was performed by KOBAS [86] (http://kobas.cbi.pku.edu.cn/). Using the same method, we also found significantly differentiated loci between the 30 reconstructed aHM genomes and 40 Han samples. Extended haplotype homozygosity (EHH) [98] was estimated with an R package, REHH [88]. A haplotype network was constructed by PopART [99] using all of the 39 SNVs located in the *GJB2* gene from the phased Panel 1 dataset. GERP +  + scores [100] were obtained from the *PGG*.SNV database [40] (https://www.pggsnv.org/).

To search HM-specific selection signals, we developed an approach with more stringent criteria. First, the allele frequency in the Han Chinese population should be fixed. Second, the difference in allele frequency should be greater than or equal to 0.3 between the reconstructed aHM and the Han Chinese population. Third, the allele frequency must be distributed in a gradient in the following order: the HM ancestral population, the present HM population, and the Han Chinese population. SNVs involved in the top 0.1% *F*_ST_ between the reconstructed HM ancestral population and the Han Chinese population but not in the top 1% *F*_ST_ between the present HM population and the Han Chinese population were considered extra SNVs.

#### Rare variants of strong effects

We focused on MAF ≤ 20% SNVs with moderate or high biological effects among 40 Han genomes. An allele with a higher frequency than the other allele was considered a major ancestral allele in East Asian populations. An allele with a lower frequency was considered a recently derived allele in East Asian populations. We calculated the proportion of individuals carrying at least one derived SNV for each gene by population. The highest frequency of a recently derived allele in each gene in the Yao population was listed in the last column (Additional file 1: Table S24). In addition, genes carrying multiple dispersed rare derived alleles of strong effect SNVs were searched according to the following standards: (1) no derived allele in the Han Chinese population; (2) more than 10% of samples carrying at least one derived allele; (3) the frequency of the highest derived allele should be less than 5% in Yao population; (4) genes carry at least two SNVs.

#### Statistical analysis

All details of the statistics applied are provided in the Supplemental Information. We have implemented Fisher’s exact test three times in R through the *ecx* command.

### Supplementary Information

**Additional file 1:** **Table S1.** Quality control information of whole genome sequencing of Yao samples. **Table S2.** Total length of Archaic segments and Ancestry segments at the individual level among HM. **Table S3.** Higheast Altai Neanderthal segments in 80 Yao samples. **Table S4.** Higheast Denisova segments in 80 Yao samples. **Table S5.** Unique Altai Neanderthal segments in 80 Yao samples. **Table S6.** Unique Denisova segments in 80 Yao samples. **Table S7.** Y-haplogroups inferred by Y-LineageTracker software based on Panel 1 dataset. **Table S8.** The distribution of Y-haplogroups in sequencing data samples. **Table S9.** Hmong-Mien language distence matrix. **Table S10.** mtDNA-haplogroups inferred by Haplogrep2 software based on Panel 1 dataset. **Table S11.** The distribution of mtDNA-haplogroups in sequencing data samples. **Table S12.** Pairwise-F_ST_ between two populations based on Panel 2 dataset in the context of East Asia. **Table S13.** Inferring the origin of the South China components in the Han Chinese population through f4. **Table S14.** 8 ancient DNA samples sharing the most genetic drift with the Hmong-Mien ancestral population. **Table S15.** The F3 results of 8 ancient DNA samples sharing the most genetic drift with the Hmong-Mien ancestral population. **Table S16.** Top 0.1% signal of the pairwise F_ST_ of HM and Han. **Table S17.** KOBAS pathway analysis on the genes in which highly differentiated SNVs between HM and Han are located. **Table S18.** Top 0.1% signal of the pairwise F_ST_ of aHM and Han. **Table S19.** KOBAS pathway analysis on the genes in which newly identified signal SNVs of aHM are located. **Table S20.** IHS results of the HM population group. **Table S21.** IHS results of the aHM population group. **Table S22.** SNVs distributed in a gradient among aHM, HM and Han. **Table S23.** KOBAS pathway analysis on the genes that regulated by eQTLs from HM special SNVs. **Table S24.** Proportion of carrying recent derived alleles. **Table S25.** 3-degree or closer relationship pairs in 80 Yao samples. **Table S26.** HLA types of all samples in Panel 1 dataset. **Table S27.** Frequency statistics of each HLA type in each population.

**Additional file 2.** This file includes Supplemental Text and Supplemental Figures S1 to S28.

## Data Availability

The script for reconstructing the ancestral genomes from this work can be found on GitHub and Zenodo, https://github.com/Shuhua-Group/Construct-ancestral-genome (10.5281/zenodo.10499683). The release of the variants of 80 Yao samples by this work is permitted by The Ministry of Science and Technology of the People’s Republic of China (permission no. 2022BAT1948) at the National Omics Data Encyclopedia (https://www.biosino.org/node) with accession number OEZ014103. All data generated or analyzed during this study are included in this published article, its supplementary information files, and publicly available repositories.

## Abbreviations

aHM: HM ancestral
EHH: Extended haplotype homozygosity
eQTL: Expression quantitative trait loci
HM: Hmong–Mien
IBD: Identity-by-descent
mtDNA: Mitochondrial DNA
MRCA: The most recent common ancestor
PC: Principal component
PCA: Principal component analysis
ROH: Run of homozygosity
SNP: Single-nucleotide polymorphism
VEP: The Ensembl Variant Effect Predictor
VQSR: Variant Quality Score Recalibration
