# Supplementary material for: Reconstructing the ancestral gene pool to uncover the origins and genetic links of Hmong–Mien speakers
Source: BMC Biol. 2024 Mar 13;22:59. doi: 10.1186/s12915-024-01838-9 (PMC10935854; doi:10.1186/s12915-024-01838-9)
Supplement: Supplementary file 2 — Additional file 2. This file includes Supplemental Text and Supplemental Figures S1 to S28. [file 12915_2024_1838_MOESM2_ESM.pdf]

Supplemental Information for

Reconstructing the ancestral gene pool to uncover the origins and genetic links of Hmong–Mien speakers

Yang Gao, Xiaoxi Zhang, Hao Chen, Yan Lu, Sen Ma, Yajun Yang, Menghan Zhang, Shuhua Xu.

* Correspondence to: xushua@fudan.edu.cn

This PDF file includes:

Supplemental Text

Supplemental Figure S1 to S28

CONTENTS

S1. Statistics and annotation on variants 4

S2. Archaic introgression segments detection 5

S3. Populations and samples 7

S4. Hmong-Mien language family 8

S5. GLOBETROTTER for Yao population 10

S6. The rare allele sharing on populations 11

S7. The model of qpGraph 12

S8. Statistical analysis 13

S9. Supplementary Figures 16

Fig.S1 | Geographic distribution of populations involved in the article. 16

Fig S2 | The genetic location of the Yao population on the phylogenetic tree in the context of East Asian populations. 17

Fig.S3 | PCA results in the context of Eurasia. 18

Fig.S4a | The initial result of admixture. 19

Fig.S4b | 20 additional results of admixture. 22

Fig.S4c | The CV error of 21 repetitions of admixture. 23

Fig.S4 | The results of 21 repetitions of admixture. 23

Fig S5 | Shared genetic drift of three present-day HM populations with other East Asian populations by outgroup F3. 24

Fig S6a | The divergence time between three HM subgroups. 25

Fig S6b | The divergence time between HM subgroups and Han. 26

Fig S6c | The divergence time between HM subgroups and Dai. 27

Fig S6 | The divergence time inferred by MSMC and MSMC-IM. 27

Fig S7 | NRY phylogenetic tree is constructed based on samples in Panel1 dataset. 28

Fig.S8 | Genetic diversity in several East Asian populations from Panel 1 dataset. 29

Fig.S9 | The changes in historical effective population size inferred by MSMC. 30

Fig.S10 | Comparison of ancestral components between autosomal and X-chromosome. 31

Fig.S11 | PCA analysis in the context of Southern East Asia 32

Fig.S12 | The length and quantity distribution of run of homozygosity (ROH) in different populations in the Panel 1 dataset.. 33
Fig.S13a | qpGraph models generated by automatic search. 34
Fig.S13b | The qpGraph model of the basic skeleton designed by us. 35
Fig.S13c | An alternative graph of Yao population not receiving the gene flow from the TK. 36
Fig.S13d | An alternative graph of the three HM populations not receiving the gene flow from the Han population. 37
Fig.S13e | An alternative graph combining missing gene flow in Fig.S13d and Fig.S13c.. 38
Fig.S13 | The admixture model of HM populations from qpGraph. 38
Fig.S14 | The present-day Hmong-Mien populations and the reconstructed ancestral population shared the most genetic drift.. 39
Fig.S15 | Inference of ancestral components of ancient DNA samples from Guangxi. 40
Fig.S16 | PCA of reconstructed HM ancestors in the context of Eurasia. 41
Fig.S17 | The reconstructed HM ancestral genome in admixture analysis......................... 42
Fig.S18 | Manhattan plot of pairwise F_{ST} for aHM, HM and Han. 43
Fig.S19 | Additional selection signals were found in HM ancestral populations reconstructed by ancestral segments. 44
Fig.S20 | Manhattan plot using the P value of iHS for HM and aHM. 45
Fig.S21 | Venn plot of HM selection signals detected by different methods. 46
Fig.S22 | Manhattan plot of pairwise F_{ST} between HM and Han. 47
Fig.S23 | Manhattan plot using the P value of iHS for Yao, Miao, She and Han. 48
Fig.S24 | Manhattan plot of pairwise XPEHH for Yao, Miao, She and Han........................ 49
Fig.S25 | Venn plot of shared signal segments identified by 4 methods in Yao. 50
Fig.S26 | The frequency of HLA-B*1502 allele in East Asia populations............................ 51
Fig.S27 | Clinically relevant SNVs carried by the population and sharing among populations. 52
Fig.S28 | The distribution of frequency differences of HDVs between Yao and Han. 53

S1. Statistics and annotation on variants

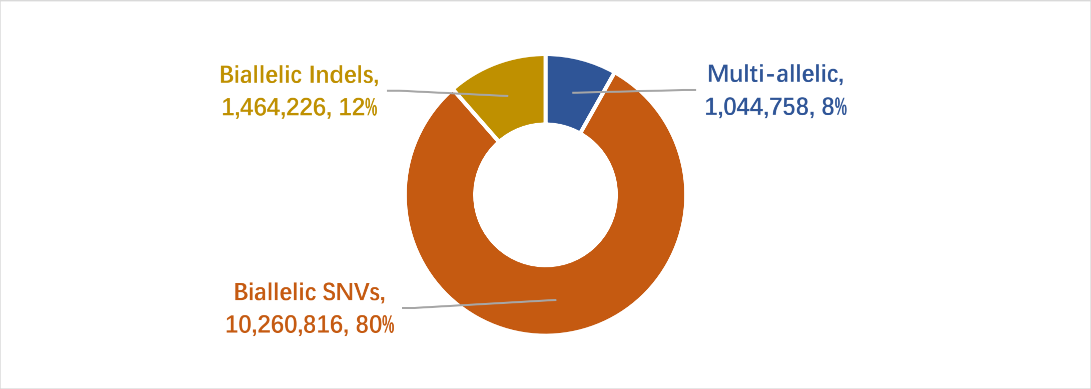

		Number of sites	dbSNP147		dbSNP154	
			Number of Novel Sites	Novelty rates	Number of Novel Sites	Novelty rates
**Multi-allelic**		1,044,758	426,152	40.79%	303,942	29.09%
**Biallelic**	SNVs	10,260,816	1,347,210	13.13%	504,927	4.92%
	Indels	1,464,226	368,635	25.18%	148,253	10.13%

We calculated the number of each type of variants on autosomal in 80 Yao samples (see the table above) based on the dataset after VQSR quality control. The biallelic SNVs are the main type of variants, accounting for 80% of the total variants. Furthermore, we annotate the biallelic SNVs with effect by VEP (see the table below). Variants that can produce multiple effects in different transcripts will be included in each effect category. Therefore, the total number of variants in the table below is greater than the actual number of variants.

Effect	Variant.type	All SNVs	Novelty SNVs
MODIFIER	3 prime UTR variant	118,146	6,358
	5 prime UTR variant	37,607	2,260
	NMD transcript variant	1,148,312	59,636
	coding sequence variant	53	5
	downstream gene variant	1,492,192	73,148
	intergenic variant	3,889,806	186,946
	non-coding transcript exon variant	215,940	10,572
	non-coding transcript variant	3,010,014	152,999
	intron variant	5,298,809	267,169
	upstream gene variant	1,451,454	72,329
	mature miRNA variant	274	7
	**Total**	**16,662,607**	**831,429**
LOW	incomplete terminal codon variant	28	3
	splice region variant	12,967	617
	stop retained variant	69	1
	synonymous variant	39,687	1,175
	**Total**	**52,751**	**1,796**
MODERATE	missense variant	48,036	2,670
	**Total**	**48,036**	**2,670**
HIGH	splice acceptor variant	597	68
	splice donor variant	886	60
	start lost	178	16
	stop gained	798	81
	stop lost	132	6
	**Total**	**2,591**	**231**
**Total**		**16,765,985**	**836,126**

S2. Archaic introgression segments detection

We integrated the Archaic fragments results of 7 populations: Yao, Miao, She, Han, Dai, Tibetan and Sherpa. Each fragment was calculated the carrying rate for each population. We focused on two types of Archaic fragments: (1) only Yao carrying (unique) and not observed in anyone else; (2) Yao with the highest carrying rate and 5% higher than everyone else.

For Yao unique segments, we identified 2744 Altai Neanderthal fragments of Yao unique with a total length of 48.34Mb. The highest frequency fragment was chr10:130578274-130582765, with a frequency of 10%, which was located in the intergenic region. chr2: 170627655-170630774, with a frequency of 7.5%, located in *KLHL23* gene, which may be related to pyridoxal 5-phosphate salvage; chr3: 65345147-65351684 and chr3:65357288-65373839 were 6.25%, which were located on the *MAGI1* gene. The product of this gene may act as a scaffold protein at the cell-cell junction; chr3:65357288-65373839, with a frequency of 6.25%, is located in the *PRR5L* gene and involves mTOR signaling and PI3K / Akt signaling pathways.

We identified 1,062 Denisova fragments of Yao unique with a total length of 15.95Mb. The highest frequency is 5%, with two segments: (1) chr12: 114480786-114607342 involves four genes, *RP11-100F15.1*, *RP11-100F15.2*, *HAUS8P1* and *GLULP5*. The first two are lincRNA genes and the last two are pseudogenes; (2) chr16: 56597152-56617724 is located in *MT4* gene and is related to metallothionein binding metal and copper homeostasis.

For Yao highest segments, we identified 622 Altai Neanderthal fragments of Yao unique with a total length of 7.24Mb. The 11 fragments with the highest frequency are in the region of chr3:50311321-50419883, with a frequency of 71.9% - 75%, involving more than 20 genes, among which *HYAL1*, *HYAL2* and *HYAL3* encode lysosomal hyaluronidase, which degrades hyaluronic acid in cells. The other nine relatively high-frequency fragments are in the region of chr14:105971720-106143750, with a

frequency of 64.4% - 68.8%, involving genes *IGHE*, *IGHA2*, *IGHG4*, *IGHG2* and *TMEM121*, which are related to immune function.

We identified 25 Denisova fragments of Yao unique with a total length of 481.9 Kb. Five fragments are in the region of chr12:115147211-115166989, with a frequency range of 33.1% - 37.5%, involving a lincRNA gene *RP4-601P9.2*.

S3. Populations and samples

The samples in this article are integrated from multiple data sets, including AAGC, HGDP, SGDP, ADP, and HumanOrigin. The samples from AAGC and HGDP/SGDP are whole genome sequencing data, and the sequencing depth of all samples is about 30x. These two parts are combined as Panel 1 dataset. The samples from HumanOrigin and ADP are genome-wide data, which are combined with the samples from AAGC as a Panel 2 dataset. There is a certain intersection between the samples from HGDP/SGDP and HumanOrigin. Han samples from AAGC are sampled from multiple locations, covering Han populations in southern China and Han populations in northern China. Therefore, the location of 40 Han samples from AAGC in the figure does not represent the actual sampling location.

In this study, the population was classified by language families. These include the Hmong-Mien language speakers represented by Yao, Miao, She, etc, the Tai-Kadai language speakers represented by Zhuang, Dong, Dai, Li, etc, the Austronesian language speakers represented by Ami, Atayal, etc, and the Sino-Tibetan language

speakers represented by Han, Tibetan, etc, and the Austroasiatic language speakers represented by Wa, Bulang, Deang, etc.

S4. Hmong-Mien language family

(1) Yao

Yao-Mianyao: It is a dialect with a large population and the widest distribution. There are county branches inhabited by Yao ethnic, and most of them use this dialect.

Yao-Biaomin: The Yao people who use Biaomin dialect are mainly distributed in Dongshan area of Quanzhou County, Guangxi, and there are also some in Guanyang adjacent to Dongshan and Dao County and Shuangpai in Hunan.

Yao-Zaomin: The Yao people who speak this dialect are mainly distributed in Liannan, Guangdong, and there are also some in Yangshan and Yizhang, Hunan.

(2) She

She language refers to the language used by the She ethnic. It belongs to the Sino-Tibetan language family and the language branch is undetermined. It is distributed in some mountainous areas of Fujian, Zhejiang, Guangdong, Jiangxi, Anhui and other provinces in China

(3) Miao

Miao language can be divided into three dialects: Miao-Chuanqiandian (more than 3 million people), Miao-Xiangxi (1 million people) and Miao-Qiandong (2.1 million

people). These three dialects together with Bunu and other languages form the Miao language branch.

Miao-Xiangxi: Also known as "Eastern dialect". It is used by 1 million people, mainly distributed in Xiangxi Prefecture, Hunan Province.

Miao-Qiandong: Also known as "Central dialect". It is used by more than 2 million people, mainly distributed in Qiandongnan Prefecture, Guizhou Province.

Miao-Chuanqiandian: Also known as "Western dialect". It is used by more than 3 million people and involves a variety of sub dialects.

Miao-Diandongbei: It belongs to the sub dialect of Miao-Chuanqiandian language, which is used by 300,000 people.

(4)Bunu

It belongs to the Miao language branch of the Hmong-Mien language family in the Sino-Tibetan language family. The use population is about 340,000, accounting for about 24% of the total population of Yao ethnic in China. It is mainly distributed in some mountainous areas of Guangxi, China, and there are also some users in some counties near Guangxi in Guizhou, Hunan and Yunnan.

Bunu-Bunu: It is mainly distributed in the west of Guangxi, some in the south of Guizhou and the southeast of Yunnan, and the population is much higher than that of other Bunu dialects.

Bunu-Baheng: It is scattered in Southwest Hunan, Southeast Guizhou and Northern Guangxi.

Bunu-Younuo: It has a small population and is distributed in several villages in Longsheng Ethnic Autonomous County and Jinxiu Yao Autonomous County, Guangxi.

Bunu-Jiongnai: Roughly the same as Bunu-Younuo.

S5. GLOBETROTTER for Yao population

```
### INFERRED SOURCES AND DATES ('best-guess' conclusion: one-
date-multiway)
########################################
### 1-DATE FIT EVIDENCE, DATE ESTIMATE, SINGLE BEST-FITTING
DONORS
gen.1date proportion.source1 maxR2fit.1date fit.quality.1event
fit.quality.2events bestmatch.event1.source1
bestmatch.event1.source2 proportion.event2.source1
bestmatch.event2.source1 bestmatch.event2.source2
38.1842471467983 0.36 0.436952345881768 0.956094753263112
0.989925704134004 Han_NChina Zhuang 0.29 Miao Zhuang
########################################
```

Here, we take Yao as the target population, and other available population data as the surrogate populations, including Ami, Atayal, Blang, Cambodian, Dai, Daur, Deang, Dong, Han, Han_NChina, Hezhen, Japanese, Korean, Lahu, Li, Miao, Mongola, Naxi, Oroqen, She, Sherpa, Thai, Tibetan, Tu, Tujia, Uygur, Wa, Xibo, Yi, Zhuang. We randomly selected 10 samples for each surrogate population with a sample size of more than 10.

According to the example of GLOBEROTTER, we executed ChromepainterV2 twice in preprocessing. To generate "copy vectors", all target and suspend individuals were

painted using both target and suspend individuals as donors. To generate "painting samples", target individuals were painted using only suspend individuals as donors.

In running GLOBEROTTER, we set parameters (prop. ind: 1, bootstrap. date. ind: 0, null. ind: 0) to detectable admixture. All other parameters use the recommended parameters in the user manual of GLOBETROTTER.

According to each generation of 29 years, the result from GLOBETROTTER is that the admixture of Yao population took place about 1.1 kya. However, because these source populations have been affected by gene flow from surrounding populations recently (Fig.2), we think this result can only show that the Yao population has been affected by gene flow from Zhuang, Han, and Miao recently. this result can not represent the time when the Yao population appeared.

S6. The rare allele sharing on populations

	Yao	Miao	She	Han	TIB
Yao	.	307,977	290,843	303,250	257,731
Miao	307,977	.	286,659	310,432	269,957
She	290,843	286,659	.	289,854	250,037
Han	303,250	310,432	289,854	.	295,242
TIB	257,731	269,957	250,037	295,242	.

We randomly select 10 individuals from Yao, Miao, She, Han and Tibetan in Panel1 (sequencing data). We focused on the rare alleles (singleton) in each group. The number of the sharing rare alleles was calculated for pairwise populations. The results were similar to the F_{ST} analysis. Compared with the She population, the Yao and Miao

populations share more rare variations with the Han population. She population is more isolated than Yao and Miao populations.

S7. The model of qpGraph

First, we run find_graph. we set numadmix from 0 to 8 and stop_gen = 100 to automatically search the model (FigS13.A).

numadmix	0	1	2	3	4
score	348.76	75.96	4.34	6.88×10^{-5}	1.38×10^{-4}
numadmix	5	6	7	8	
score	2.87×10^{-5}	9.84×10^{-6}	3.42×10^{-5}	9.84×10^{-6}	

When numadmix is equal to 0, we still observe branches with a length of 0 (Fig. S13A), which may be the reason for the high genetic similarity of the population in the model. In addition, we observed that there was a large deviation between the admixture model generated by automatic search and the real population history (Fig. S13A). Therefore, we designed the basic skeleton of the model based on our analysis results. For example, the Yao received the gene flow from the Zhuang (Fig. 2A, Fig. S5), the ancestor of Han population received gene flow from HM ancestors (Fig. 1D & Table S11). HM and TK share an ancestor (Fig. 1D), Yao first separated from HM population (Fig1.A&E&F, Fig. S5), and the Yao, Miao, and She received the recent genetic contribution from the Han population (Fig2.A), etc. Our model got a score of 7.53×10^{-6} (Fig.S13B), which is better than the model generated by automatic search with the same times (numadmix = 7) of admixture.We verify our model through alternative graphs. The score in the model

of Yao population not receiving the gene flow from the TK was 0.12 (Fig.S13C). The score in the model of the three HM populations not receiving the gene flow from the Han population was 159.00 (Fig.S13D). The score of the model with both gene flows deleted above is 398.29 (Fig.S13E).

S8. Statistical analysis

Fisher's Exact Test for the relationship between HM ancestral and IBD fragments. We compared the H-M ancestral fragment from local ancestral inference and a homologous fragment from IBD analysis at the haplotype level for 30 H-M samples used in the reconstruction of ancestral genomes. We designed the above contingency tableand tested whether there was a significant correlation between the above two fragments through Fisher's exact test.

(Both HM ancestral and IBD fragments)<br>26982679.18	(HM ancestral fragments, not IBD fragments)<br>659611995.60
(IBD fragments, not HM ancestral fragments)<br>83666672.65	(Neither IBD nor ancestral fragments)<br>2229738652.56

```
Fisher's Exact Test for Count Data
data:   m
p-value < 0.00000000000000022204460492503131
alternative hypothesis: true odds ratio is not equal to 1
95 percent confidence interval:
  1.07498970394537107786 4 1.10555876012940257346 9
sample estimates:
                    odds ratio
1.09020019170445547906 9
```

Fisher's Exact Test for the number of eQTLs and significant SNVs.

To identify variants affected by natural selection in H-M populations, we used the Han Chinese population as the reference population. The loci with large F_{ST}(top 0.1%) are considered to be affected by natural selection. All the SNVs were annotated by GTEx database v7 (https://gtexportal.org/). We designed the following contingency table to test. And there was a significant correlation between the above two fragments through Fisher's exact test.

	Top 0.1% F_{ST}	Not Top 0.1% F_{ST}
eQTL	5,535	2,033,335
Not eQTL	7,645	11,133,620

```
> fisher.test(m)
  Fisher's Exact Test for Count Data
data:   m
p-value < 0.00000000000000022204460492503131
alternative hypothesis: true odds ratio is not equal to 1
95 percent confidence interval:
  3.829025142076214471842 4.104560861340387667440
sample estimates:
                    odds ratio
3.964372405158349543797
```

Fisher's Exact Test for the relationship between genetic relatives and the carrying derived alleles.

To identify these strong effects rare variants whether showed familial aggregation for 7 genes we selected, we used all 80 Yao samples to traverse the combination of two

samples. Among the 42 loci of the seven genes, only two samples with the same SNV carry at least one derived allele (see Methods, 'Selection'), which is considered to be carried by this combination. We cover four categories: (1) The two samples were genetically related and carried common derived allele; (2) The two samples were genetically related but did not carry common derived allele; (3) The two samples were not genetically related and carried common derived allele; (4) The two samples were not genetically related but did not carry common derived allele. We designed the following contingency table to test. And there was a significant correlation between the genetic relatives and carrying derived alleles.

	Genetic relatives	Not genetic relatives
Common derived alleles	4	35
Without common derived alleles	20	3,101

```
Fisher's Exact Test for Count Data
data:    m
p-value = 0.00017639224770604153843
alternative hypothesis: true odds ratio is not equal to 1
95 percent confidence interval:
   4.17181698572738124397 6 56.681153975292652091866
sample estimates:
                    odds ratio
17.647521629855926761 37
```

S9. Supplementary Figures

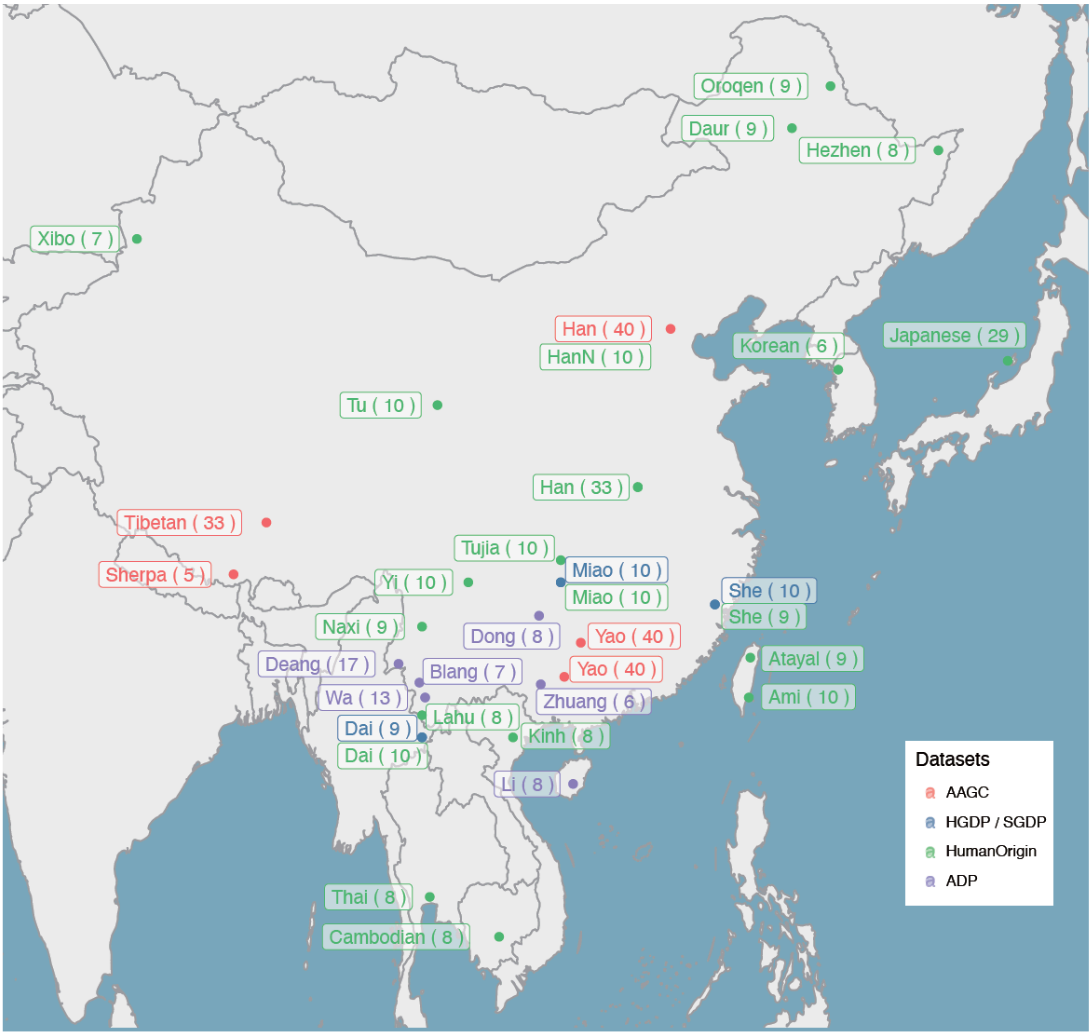

Fig.S1 | Geographic distribution of populations involved in the article.

This picture shows the geographical distribution of the populations involved in this article. The red part is from the Asian Admixed Genomes Consortium (AAGC), and all the samples shown in the figure are generated by ourselves. The blue part is from the Human Genome Diversity Project (HGDP) and the Simons Genome Diversity Project (SGDP). The green part is from the Affymetrix Human Origins genotyping dataset (HumanOrigin), and the purple part is from the East Asian population from the Asian Diversity Project (ADP).

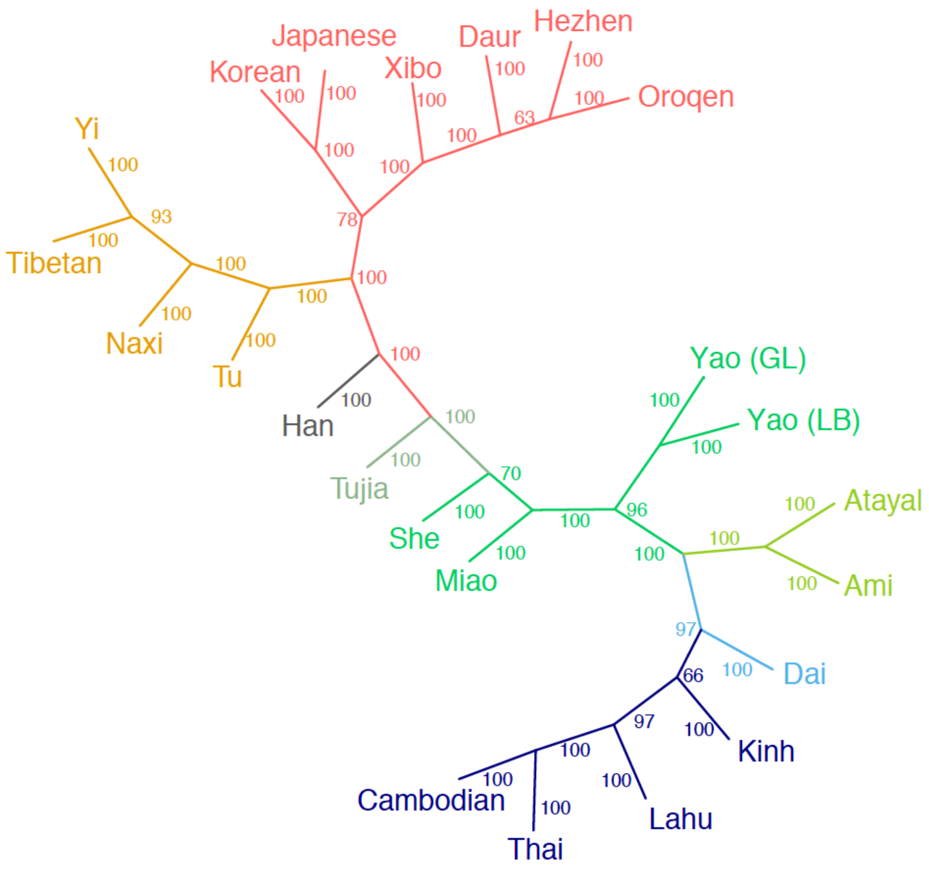

Fig S2 | The genetic location of the Yao population on the phylogenetic tree in the context of East Asian populations.

We controlled the quality of the data by filtering out the variation with deletion rate > 1% and MAF < 5%. Based on the frequency information of each variant in each population, we first used the *seqboot* command in the PHYLIP to sample 100 times, and then used the *contml* command to build a population tree for each sampling result. In addition, we also specify the "-J" parameter as 10 to random input order of populations 10 times for each tree. Finally, the result of 100 bootstraps generates a consensus tree through the *consense* command.

From the results, the newly collected Yao samples from two sampling points are close together. Yao people are located in the southernmost part of Hmong-Mien speakers, and have a close relationship with Taiwan aboriginal people and Tai-Kadai speakers. Colors represent different language families or genetic regions. Red and orange represent the Northeast and Northwest genetic groups. She|Miao\Yao are HM populations. Atayal\Ami are Taiwan aboriginal people. Dai is Tai-Kadai population. Dark blue represents the southern genetic group.

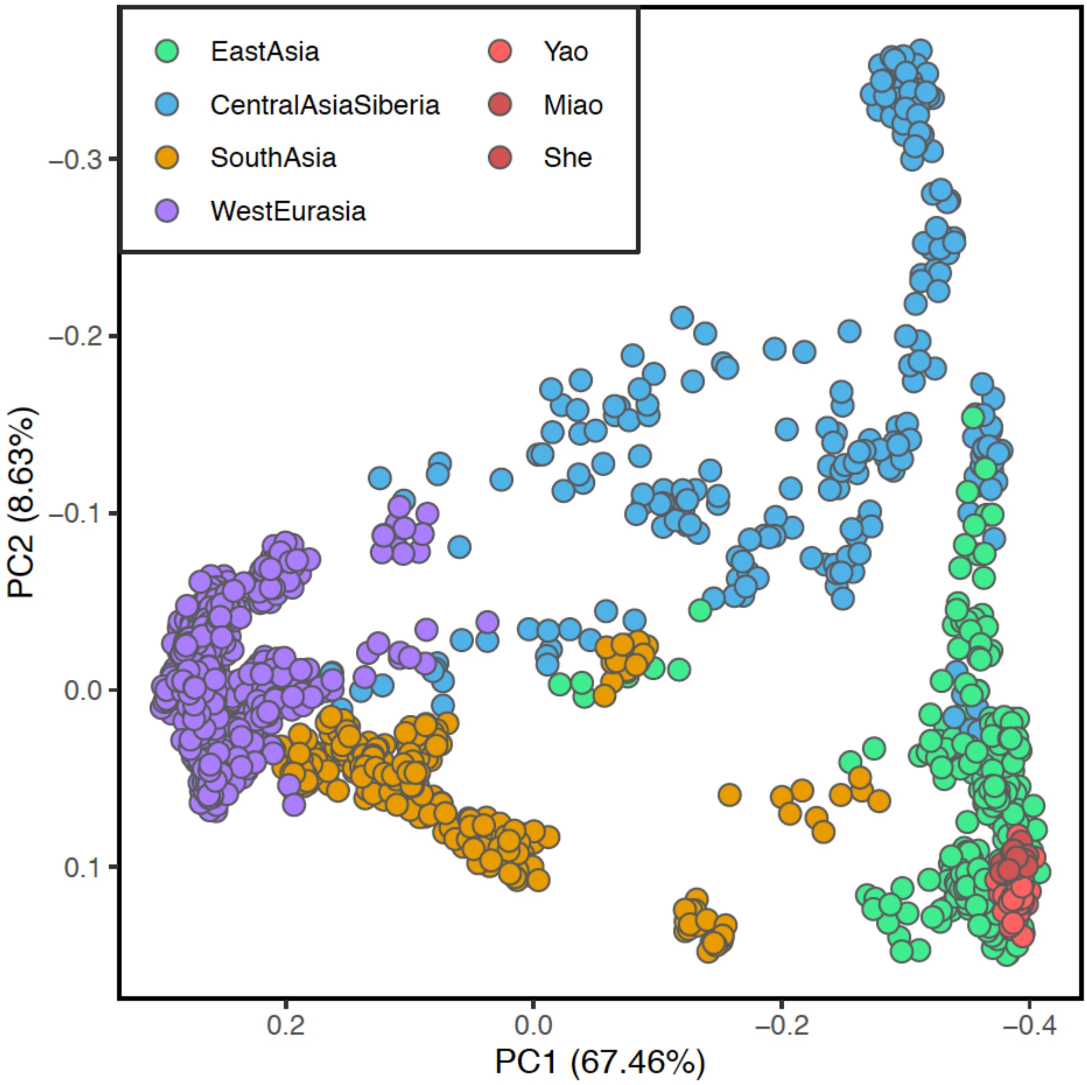

Fig.S3 | PCA results in the context of Eurasia.

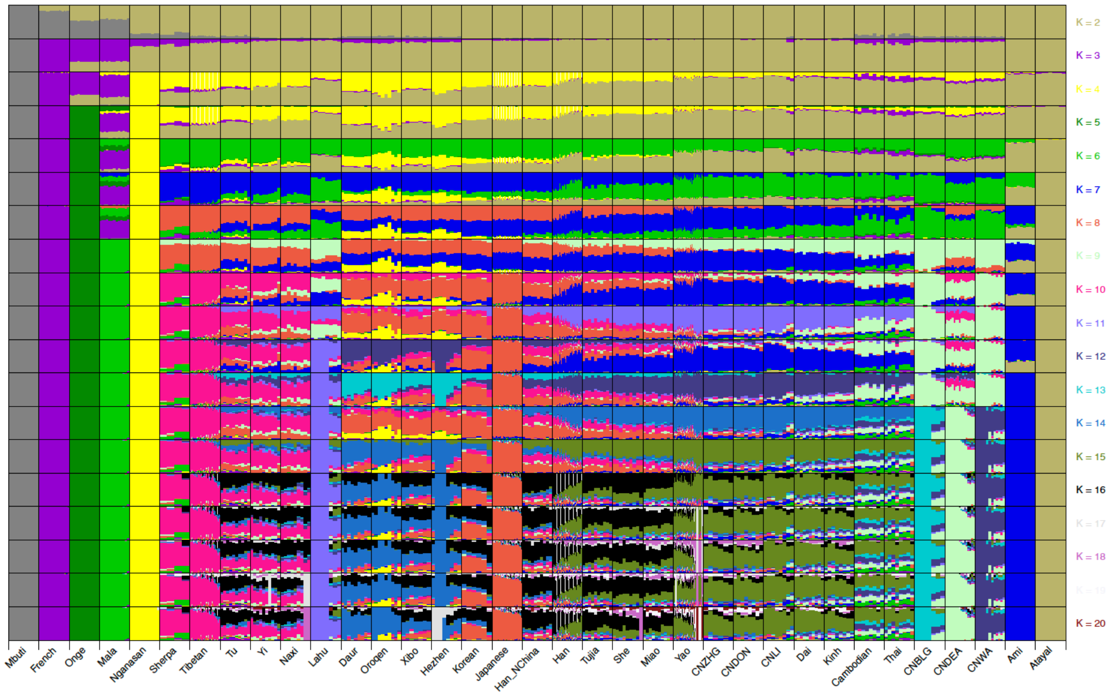

Fig.S4a | The initial result of admixture.

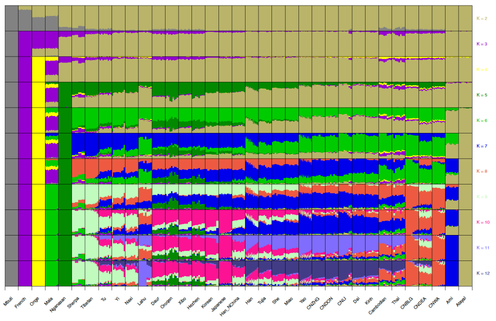
K = 2
K = 3
K = 4
K = 5
K = 6
K = 7
K = 8
K = 9
K = 10
K = 11
K = 12
Mbuti French Onge Mala Nganasan Sherpa Tibetan Tu Yi Naxi Lahu Daur Oroqen Xibo Hezhen Korean Japanese Han_NChina Han Tujia She Miao Yao CNZHG CNDON CNLI Dai Kinh Cambodian Thai CNBLG CNDEA CNWA Ami Atayal

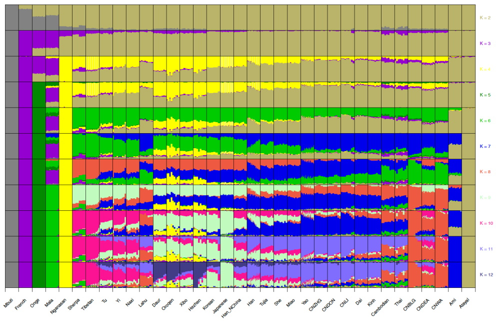
K = 2
K = 3
K = 4
K = 5
K = 6
K = 7
K = 8
K = 9
K = 10
K = 11
K = 12
Mbuti French Onge Mala Nganasan Sherpa Tibetan Tu Yi Naxi Lahu Daur Oroqen Xibo Hezhen Korean Japanese Han_NChina Han Tujia She Miao Yao CNZHG CNDON CNLI Dai Kinh Cambodian Thai CNBLG CNDEA CNWA Ami Atayal

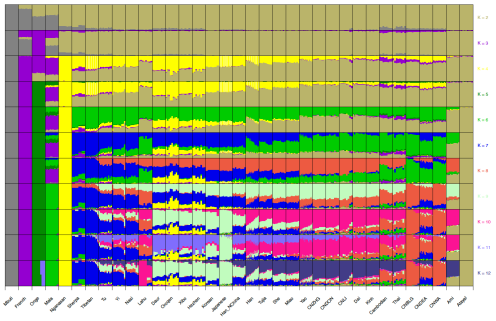
K = 2
K = 3
K = 4
K = 5
K = 6
K = 7
K = 8
K = 9
K = 10
K = 11
K = 12
Mbuti French Onge Mala Nganasan Sherpa Tibetan Tu Yi Naxi Lahu Daur Oroqen Xibo Hezhen Korean Japanese Han_NChina Han Tujia She Miao Yao CNZHG CNDON CNLI Dai Kinh Cambodian Thai CNBLG CNDEA CNWA Ami Atayal

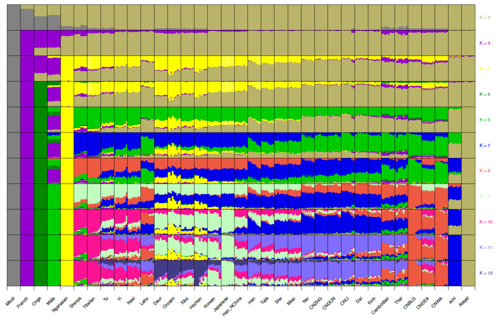
K = 2
K = 3
K = 4
K = 5
K = 6
K = 7
K = 8
K = 9
K = 10
K = 11
K = 12
Mbuti French Onge Mala Nganasan Sherpa Tibetan Tu Yi Naxi Lahu Daur Oroqen Xibo Hezhen Korean Japanese Han_NChina Han Tujia She Miao Yao CNZHG CNDON CNLI Dai Kinh Cambodian Thai CNBLG CNDEA CNWA Ami Atayal

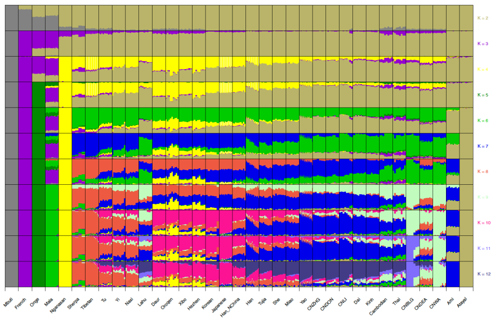
K = 2
K = 3
K = 4
K = 5
K = 6
K = 7
K = 8
K = 9
K = 10
K = 11
K = 12
Mbuti French Onge Mala Nganasan Sherpa Tibetan Tu Yi Naxi Lahu Daur Oroqen Xibo Hezhen Korean Japanese Han_NChina Han Tujia She Miao Yao CNZHG CNDON CNLI Dai Kinh Cambodian Thai CNBLG CNDEA CNWA Ami Atayal

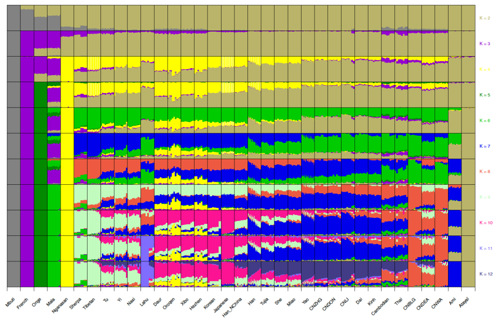
K = 2
K = 3
K = 4
K = 5
K = 6
K = 7
K = 8
K = 9
K = 10
K = 11
K = 12
Mbuti French Onge Mala Nganasan Sherpa Tibetan Tu Yi Naxi Lahu Daur Oroqen Xibo Hezhen Korean Japanese Han_NChina Han Tujia She Miao Yao CNZHG CNDON CNLI Dai Kinh Cambodian Thai CNBLG CNDEA CNWA Ami Atayal

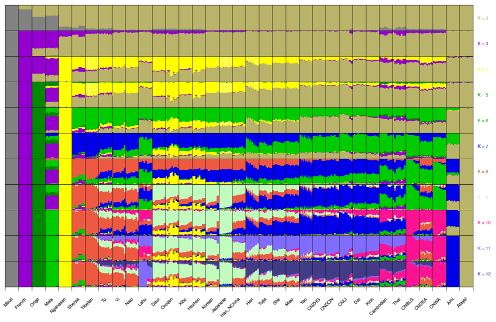
K = 2
K = 3
K = 4
K = 5
K = 6
K = 7
K = 8
K = 9
K = 10
K = 11
K = 12
Mbuti French Onge Mala Nganasan Sherpa Tibetan Tu Yi Naxi Lahu Daur Oroqen Xibo Hezhen Korean Japanese Han_NChina Han Tujia She Miao Yao CNZHG CNDON CNLI Dai Kinh Cambodian Thai CNBLG CNDEA CNWA Ami Atayal

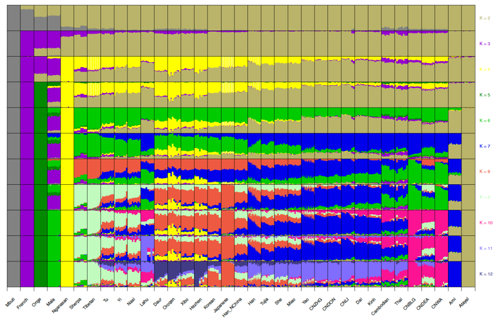
K = 2
K = 3
K = 4
K = 5
K = 6
K = 7
K = 8
K = 9
K = 10
K = 11
K = 12
Mbuti French Onge Mala Nganasan Sherpa Tibetan Tu Yi Naxi Lahu Daur Oroqen Xibo Hezhen Korean Japanese Han_NChina Han Tujia She Miao Yao CNZHG CNDON CNLI Dai Kinh Cambodian Thai CNBLG CNDEA CNWA Ami Atayal

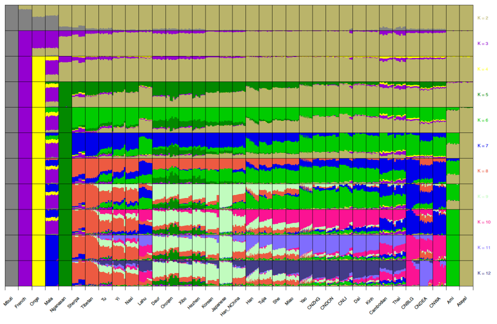
K = 2
K = 3
K = 4
K = 5
K = 6
K = 7
K = 8
K = 9
K = 10
K = 11
K = 12
Mbuti
French
Onge
Mala
Nganasan
Sherpa
Tibetan
Tu
Yi
Naxi
Lahu
Daur
Oroqen
Xibo
Hezhen
Korean
Japanese
Han_NChina
Han
Tujia
She
Miao
Yao
CNZHG
CNDON
CNLI
Dai
Kinh
Cambodian
Thai
CNBLG
CNDEA
CNWA
Ami
Atayal

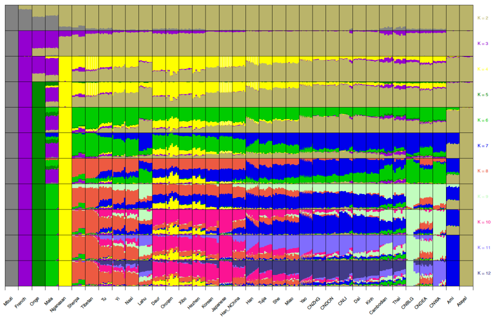
K = 2
K = 3
K = 4
K = 5
K = 6
K = 7
K = 8
K = 9
K = 10
K = 11
K = 12
Mbuti
French
Onge
Mala
Nganasan
Sherpa
Tibetan
Tu
Yi
Naxi
Lahu
Daur
Oroqen
Xibo
Hezhen
Korean
Japanese
Han_NChina
Han
Tujia
She
Miao
Yao
CNZHG
CNDON
CNLI
Dai
Kinh
Cambodian
Thai
CNBLG
CNDEA
CNWA
Ami
Atayal

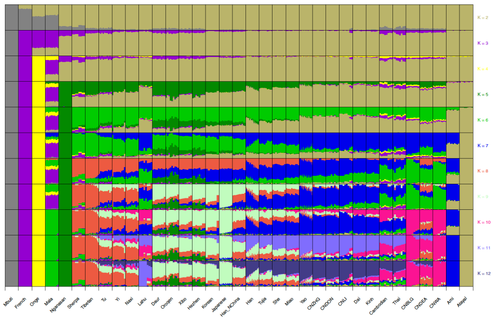
K = 2
K = 3
K = 4
K = 5
K = 6
K = 7
K = 8
K = 9
K = 10
K = 11
K = 12
Mbuti
French
Onge
Mala
Nganasan
Sherpa
Tibetan
Tu
Yi
Naxi
Lahu
Daur
Oroqen
Xibo
Hezhen
Korean
Japanese
Han_NChina
Han
Tujia
She
Miao
Yao
CNZHG
CNDON
CNLI
Dai
Kinh
Cambodian
Thai
CNBLG
CNDEA
CNWA
Ami
Atayal

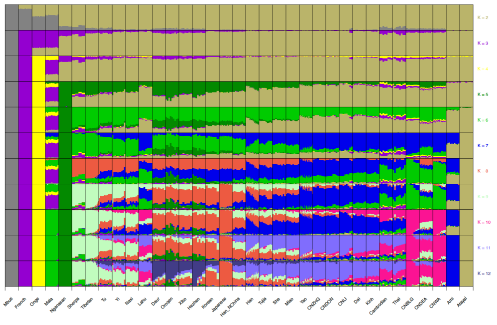
K = 2
K = 3
K = 4
K = 5
K = 6
K = 7
K = 8
K = 9
K = 10
K = 11
K = 12
Mbuti
French
Onge
Mala
Nganasan
Sherpa
Tibetan
Tu
Yi
Naxi
Lahu
Daur
Oroqen
Xibo
Hezhen
Korean
Japanese
Han_NChina
Han
Tujia
She
Miao
Yao
CNZHG
CNDON
CNLI
Dai
Kinh
Cambodian
Thai
CNBLG
CNDEA
CNWA
Ami
Atayal

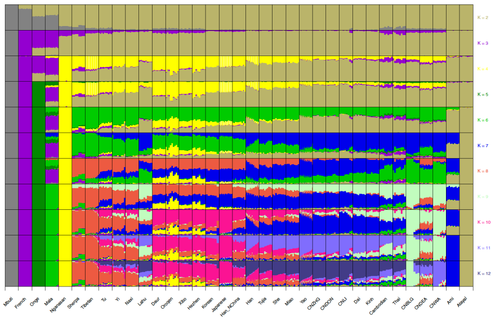
K = 2
K = 3
K = 4
K = 5
K = 6
K = 7
K = 8
K = 9
K = 10
K = 11
K = 12
Mbuti
French
Onge
Mala
Nganasan
Sherpa
Tibetan
Tu
Yi
Naxi
Lahu
Daur
Oroqen
Xibo
Hezhen
Korean
Japanese
Han_NChina
Han
Tujia
She
Miao
Yao
CNZHG
CNDON
CNLI
Dai
Kinh
Cambodian
Thai
CNBLG
CNDEA
CNWA
Ami
Atayal

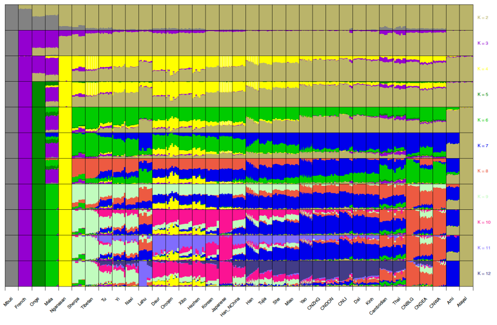
K = 2
K = 3
K = 4
K = 5
K = 6
K = 7
K = 8
K = 9
K = 10
K = 11
K = 12
Mbuti
French
Onge
Mala
Nganasan
Sherpa
Tibetan
Tu
Yi
Naxi
Lahu
Daur
Oroqen
Xibo
Hezhen
Korean
Japanese
Han_NChina
Han
Tujia
She
Miao
Yao
CNZHG
CNDON
CNLI
Dai
Kinh
Cambodian
Thai
CNBLG
CNDEA
CNWA
Ami
Atayal

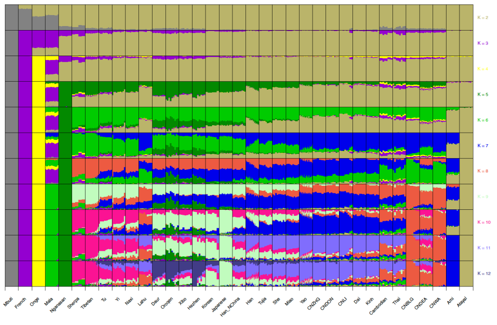
K = 2
K = 3
K = 4
K = 5
K = 6
K = 7
K = 8
K = 9
K = 10
K = 11
K = 12
Mbuti
French
Onge
Mala
Nganasan
Sherpa
Tibetan
Tu
Yi
Naxi
Lahu
Daur
Oroqen
Xibo
Hezhen
Korean
Japanese
Han_NChina
Han
Tujia
She
Miao
Yao
CNZHG
CNDON
CNLI
Dai
Kinh
Cambodian
Thai
CNBLG
CNDEA
CNWA
Ami
Atayal

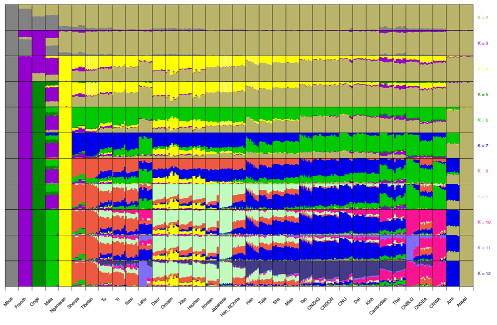
K = 2
K = 3
K = 4
K = 5
K = 6
K = 7
K = 8
K = 9
K = 10
K = 11
K = 12
Mbuti
French
Onge
Mala
Nganasan
Sherpa
Tibetan
Tu
Yi
Naxi
Lahu
Daur
Oroqen
Xibo
Hezhen
Korean
Japanese
Han_NChina
Han
Tujia
She
Miao
Yao
CNZHG
CNDON
CNLI
Dai
Kinh
Cambodian
Thai
CNBLG
CNDEA
CNWA
Ami
Atayal

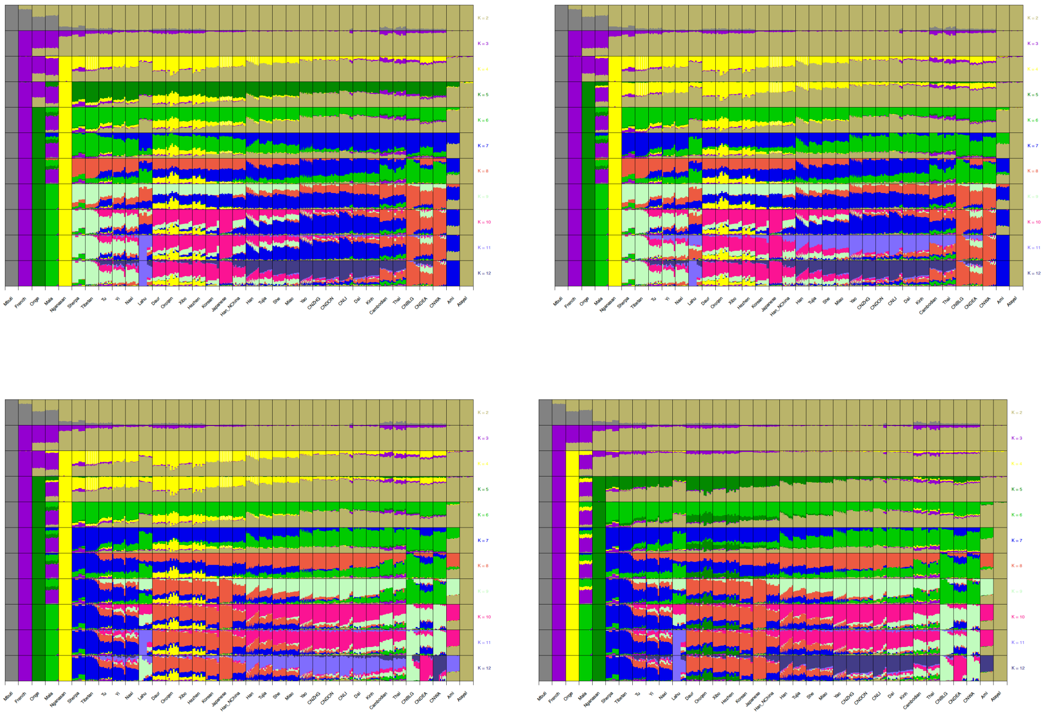

Fig.S4b | 20 additional results of admixture.

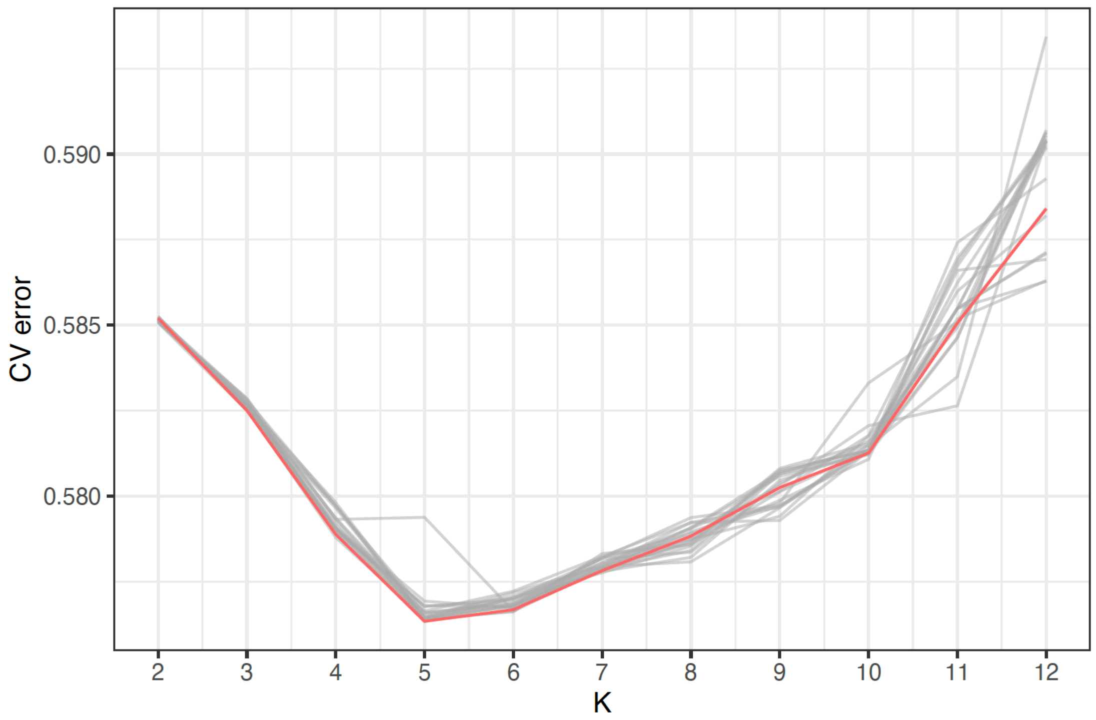

Fig.S4c | The CV error of 21 repetitions of admixture.

The red line on the CV error is the initial result we finally adopted.

Fig.S4 | The results of 21 repetitions of admixture.

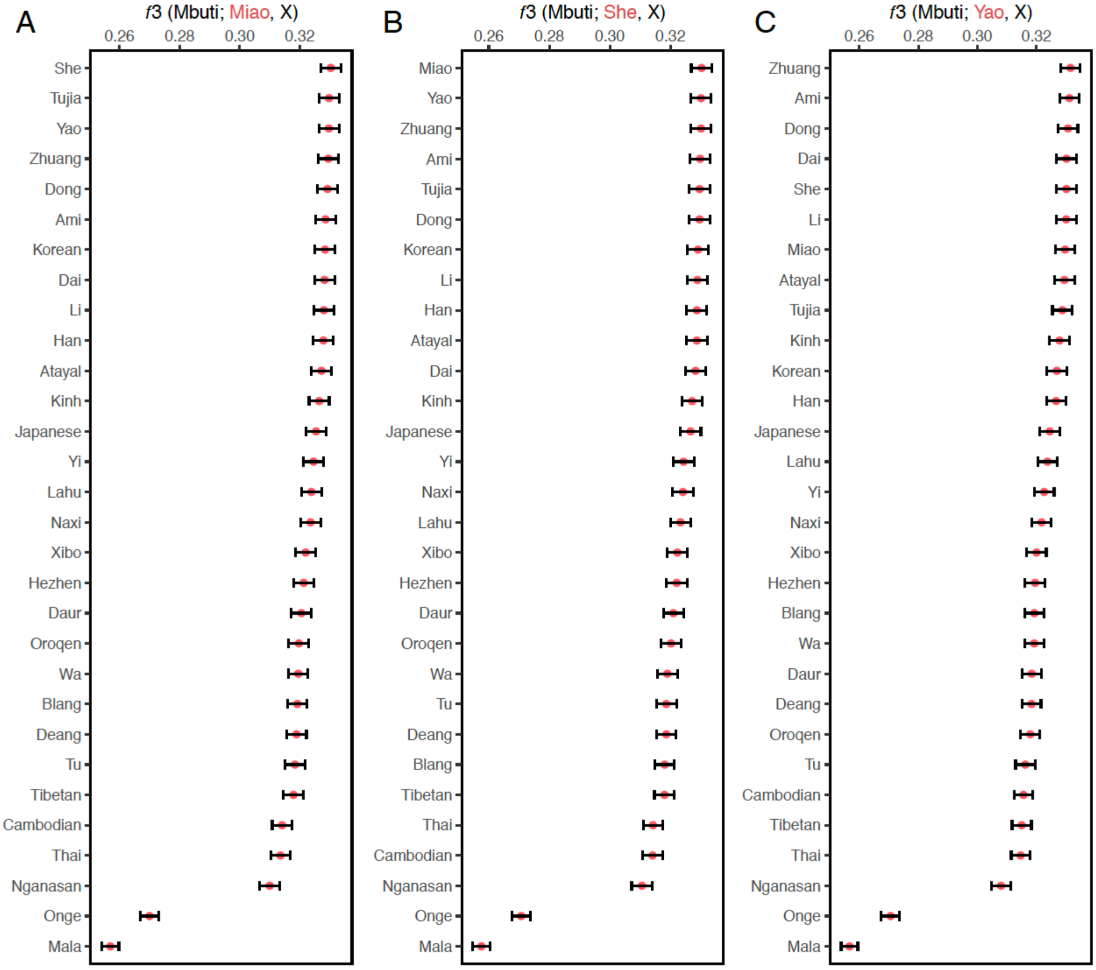

Fig S5 | Shared genetic drift of three present-day HM populations with other East Asian populations by outgroup F3.

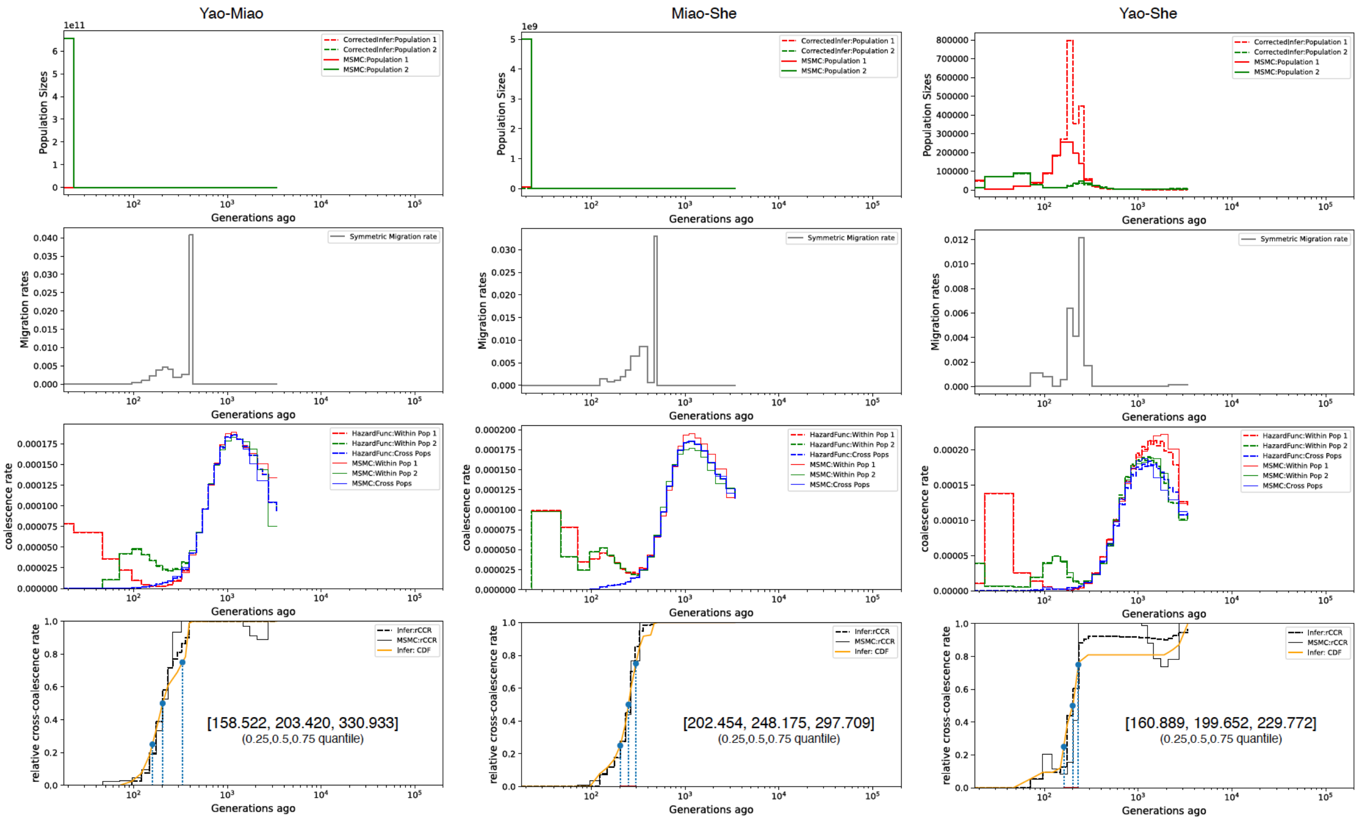

Fig S6a | The divergence time between three HM subgroups.

The divergence between the Yao population and the Miao/She population occurred ~200 generations ago, while the divergence between the Miao and She population occurred ~248 generations ago. This contradictory model means that after population differences, there is still varying degrees of gene flow between the three populations

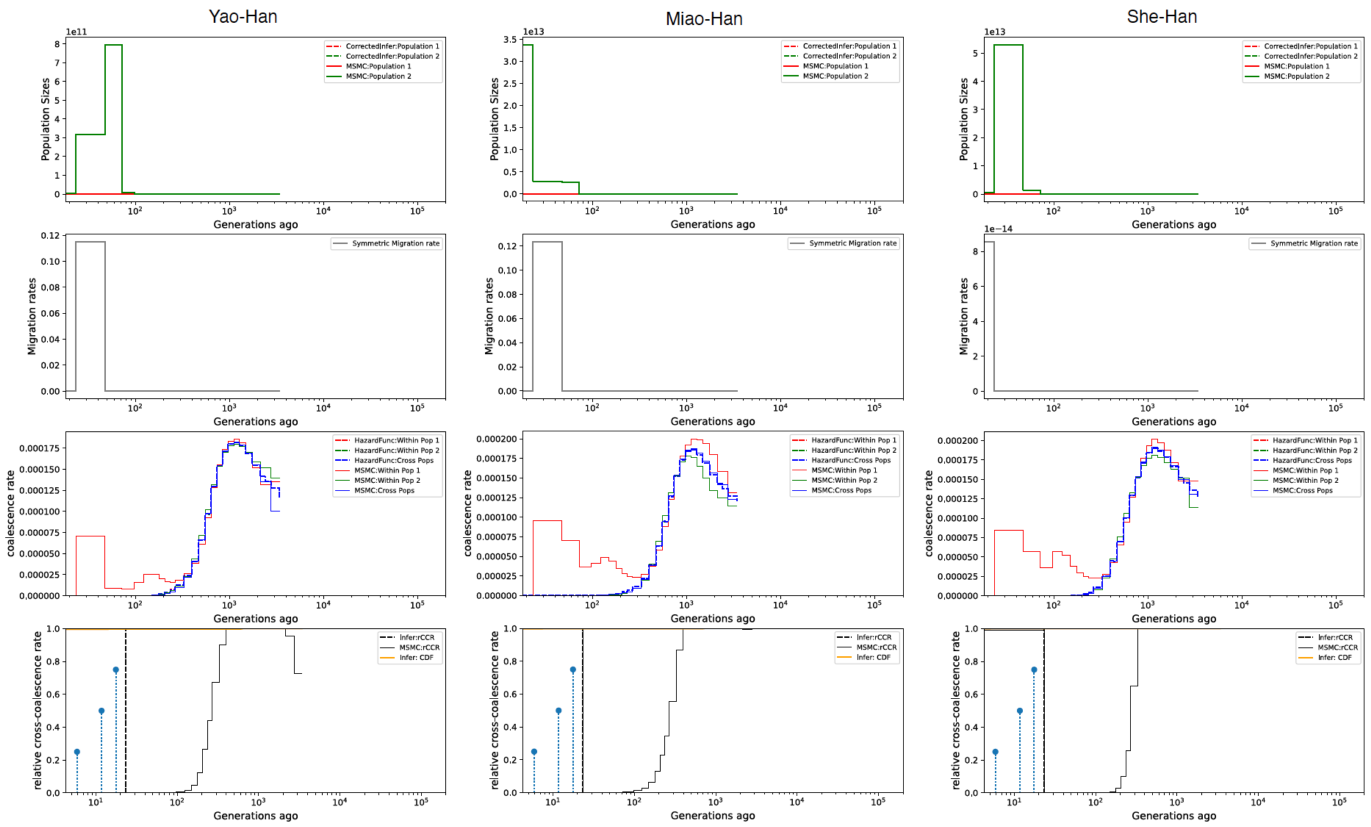

Fig S6b | The divergence time between HM subgroups and Han.

The divergence time between three HM subgroups and Han population estimated by MSMC is closer than the divergence time among the three subgroups, and MSMC-IM infers that there is recent genetic admixture between the three HM subgroups and Han. Therefore, we did not adopt this divergence time.

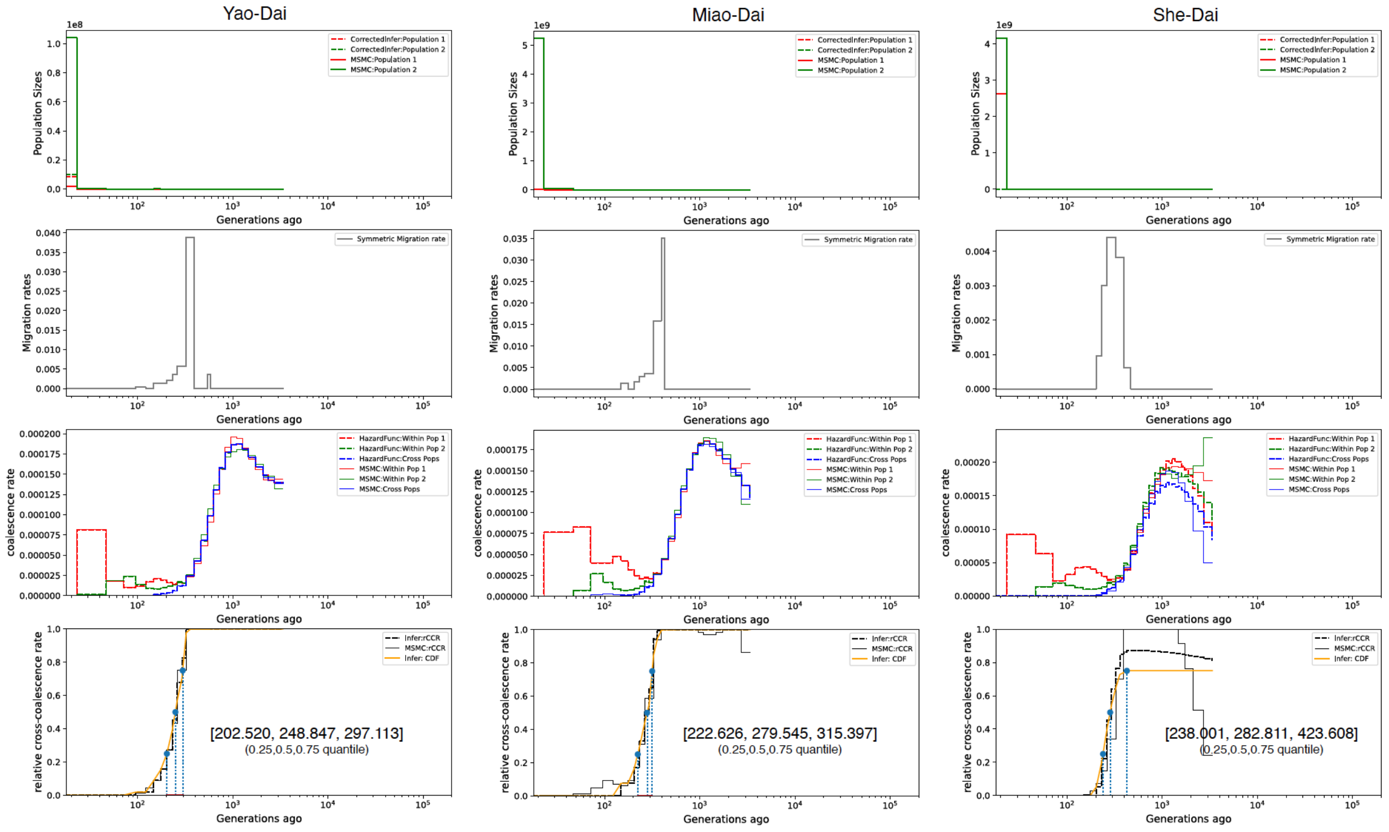

Fig S6c | The divergence time between HM subgroups and Dai.

In the analysis of ancestral inference, we found that Hmong-Mien population and Tai-Kadai population share a large proportion of the genetic component, and they share an ancestral population (Fig.1 d). To this end, we evaluated the divergence time between Hmong-Mien speakers and Tai-Kadai speakers.

The divergence time between Miao and Dai, as well as the divergence time between She and Dai, is before 280 generations, but the divergence time between Yao and Dai is before 249 generations, which is even consistent with the divergence time between Miao and She. We speculate that the recent gene flow between Yao and Dai may have influenced this result.

Fig S6 | The divergence time inferred by MSMC and MSMC-IM.

According to the MSMC software recommendation process, we estimate the divergence time between two populations using 8 haplotypes. Then, further analysis was conducted according to the recommended parameters of MSMC-IM (-beta 1e-8,1e-6 --printfittingdetails --plotfittingdetails --xlog).

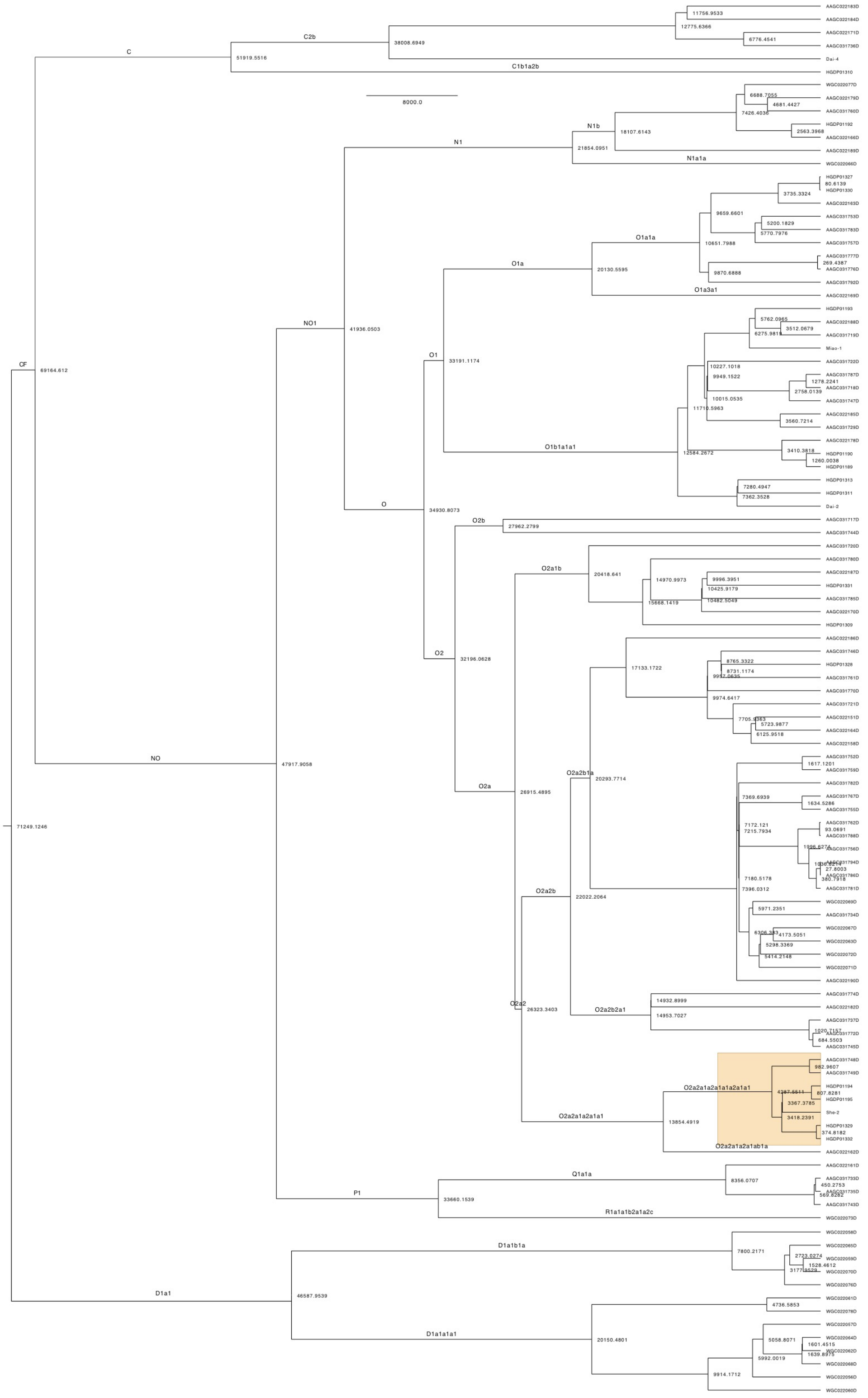

Fig S7 | NRY phylogenetic tree is constructed based on samples in Panel1 dataset.

(see Methods, 'Y chromosome and mtDNA haplogroups')

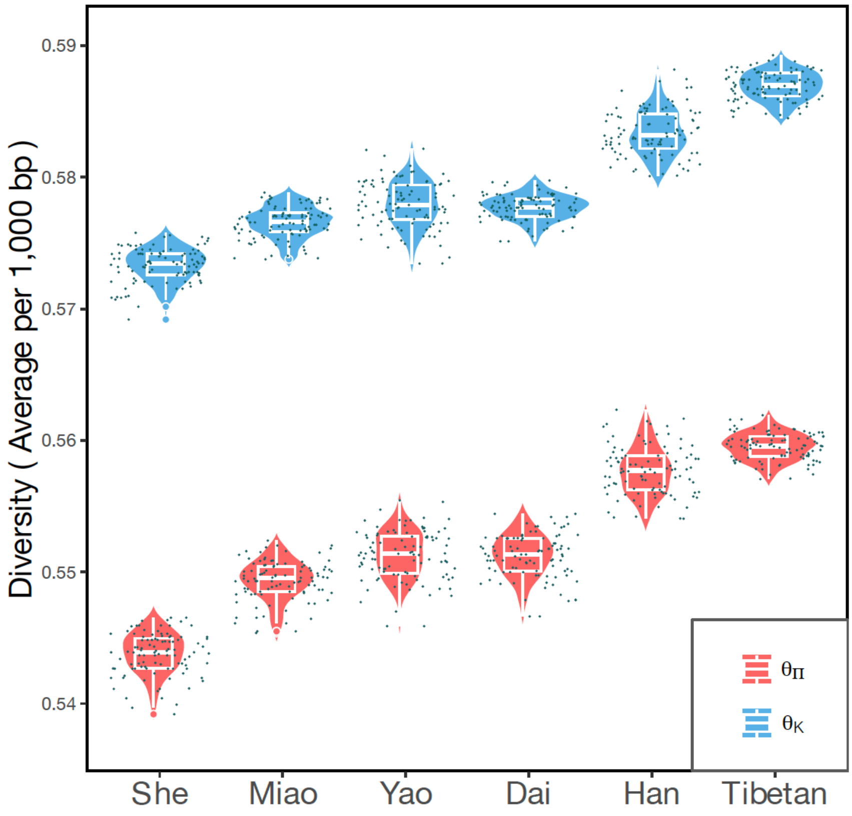

Fig.S8 | Genetic diversity in several East Asian populations from Panel 1 dataset.

We estimated population genetic diversity through the diversity parameters θ_π and θ_K. The θ_π represents average pairwise differences, that is, the average number of differential loci between two haploids. The θ_K represents number of aggregating sites, which is affected by the sample size. We randomly selected 5 samples from each population at a time, calculated θ_K and θ_π for each population at the whole genome level, and performed 100 times. The figure above shows the results of these 100 calculations. Each point represents a repetition.

The θ_K of these East Asian populations were lower than θ_π, and θ_K and θ_π showed a consistent trend. For θ_π, the genetic diversity of Yao people is lower than that of Han and Tibetan people belonging to Sino-Tibetan language family, equivalent to that of Dai people belonging to Tai-Kadai language family, and higher than that of She and Miao people belonging to Hmong-Mien language family. The difference of genetic diversity may be caused by many factors, including the recent admixture between Yao population and Tai-Kadai speakers, the bottleneck effect in she and Miao population, and the inbreeding in She population, etc.

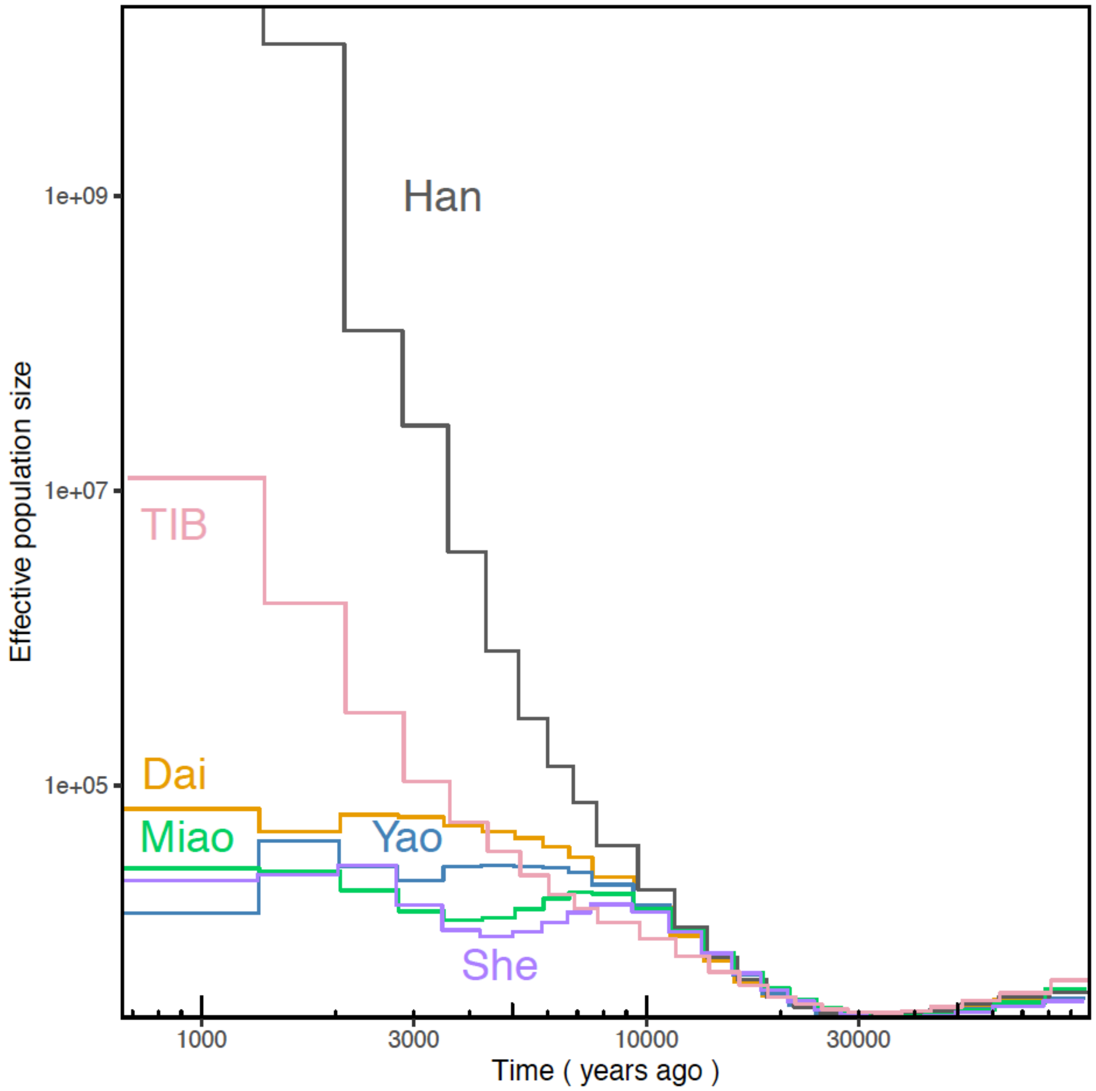

Fig.S9 | The changes in historical effective population size inferred by MSMC.

We estimated the historical changes of effective population size of several populations in the Panel 1 dataset. We selected 4 samples for each population, and other processing details were consistent with the method of estimating divergence time by MSMC (see Methods, 'Divergence time'). From the results, both She and Miao populations experienced a bottleneck event after the divergence of Hmong-Mien populations. This result also supports the conclusion that the Yao population are separated from Hmong-Mien speakers first, while She and Miao populations separated later.

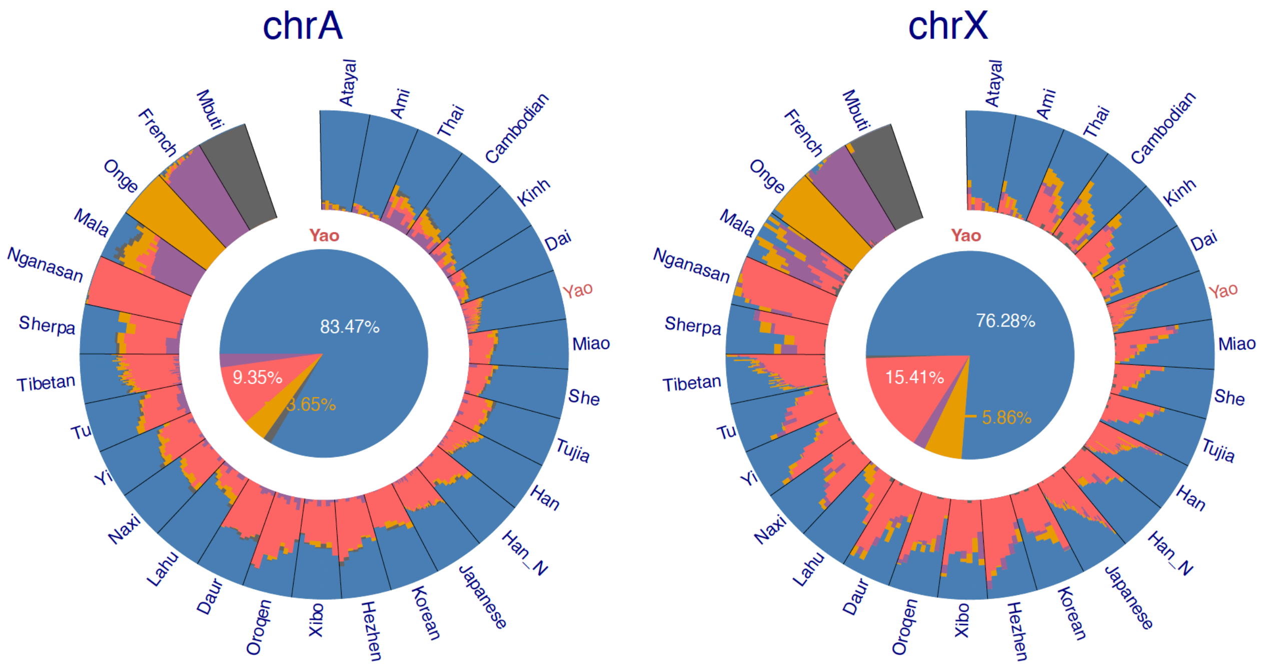

Fig.S10 | Comparison of ancestral components between autosomal and X-chromosome.

The sex-biased admixture can be identified by comparing the comparison of ancestral components between autosomal and X-chromosome. Using the part of X-chromosome in Panel 2 dataset, we labeled male X-chromosome as haploid through the haploid mode of ADMIXTURE software to infer the ancestral component of X chromosome. We compare the inference results when K = 5. To avoid the possible impact of the number of loci, we selected loci at a spacing of 1.6Mb in autosomal, and ultimately used ~1600 loci for both autosomal and X-chromosome analyses.

Firstly, the ancestral component pattern of each population from East Asia on X chromosome is basically the same as that on autosomes. The Yao population is mainly composed of Southern components and a small part of northern components. However, the proportion of northern and southern components in the X chromosome is higher than that in the autosome, including more men from the south and women from the north. Secondly, the proportion of the Onge component (yellow part) carried by East Asian populations on X chromosome is significantly higher than that on autosomal. The variance in the proportion of ancestral composition on X-chromosome is also greater than that on autosomes. Both phenomena may be due to the fact that the admixture rate of X-chromosome is slower than that of autosomes (about two-thirds of that of autosomes).

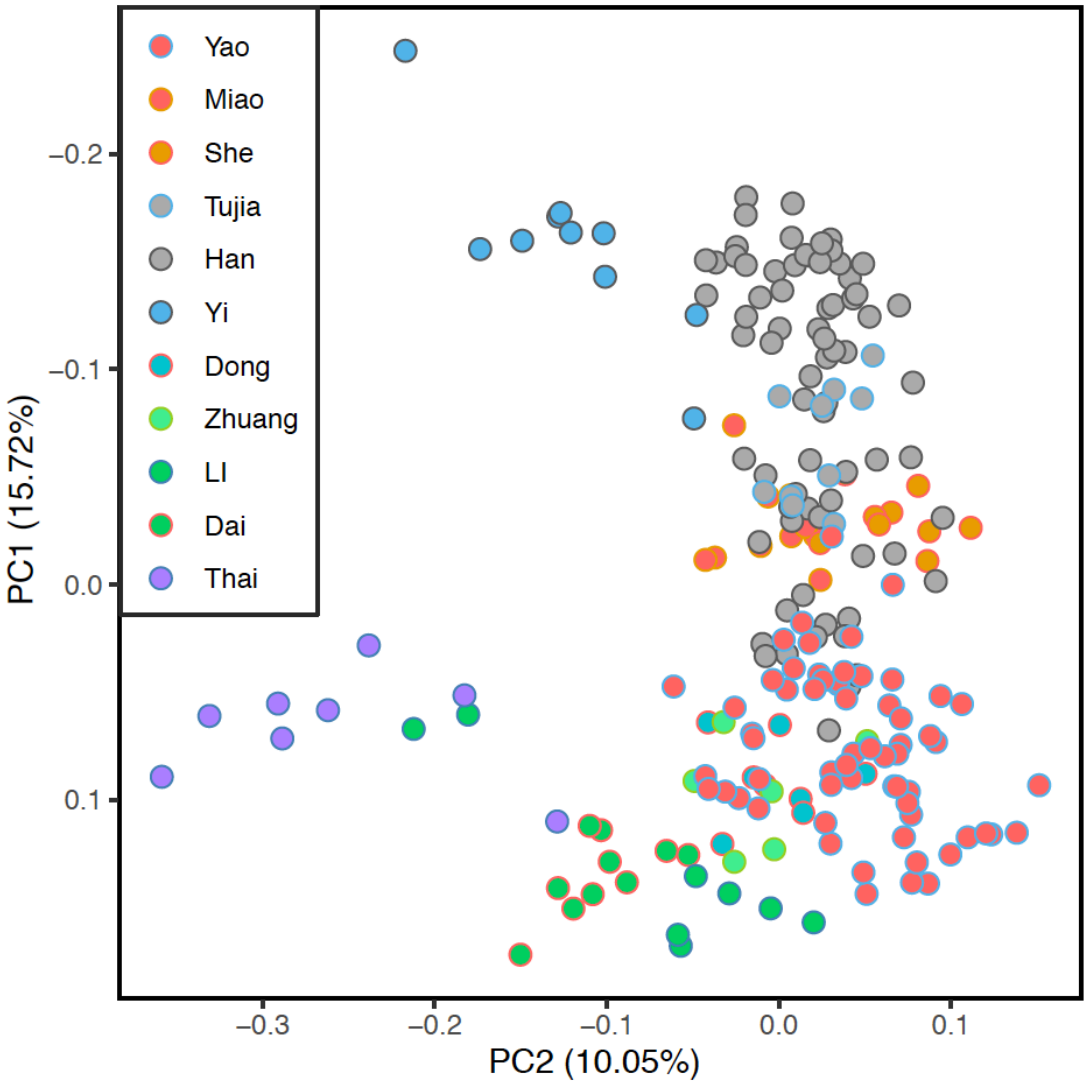

Fig.S11 | PCA analysis in the context of Southern East Asia

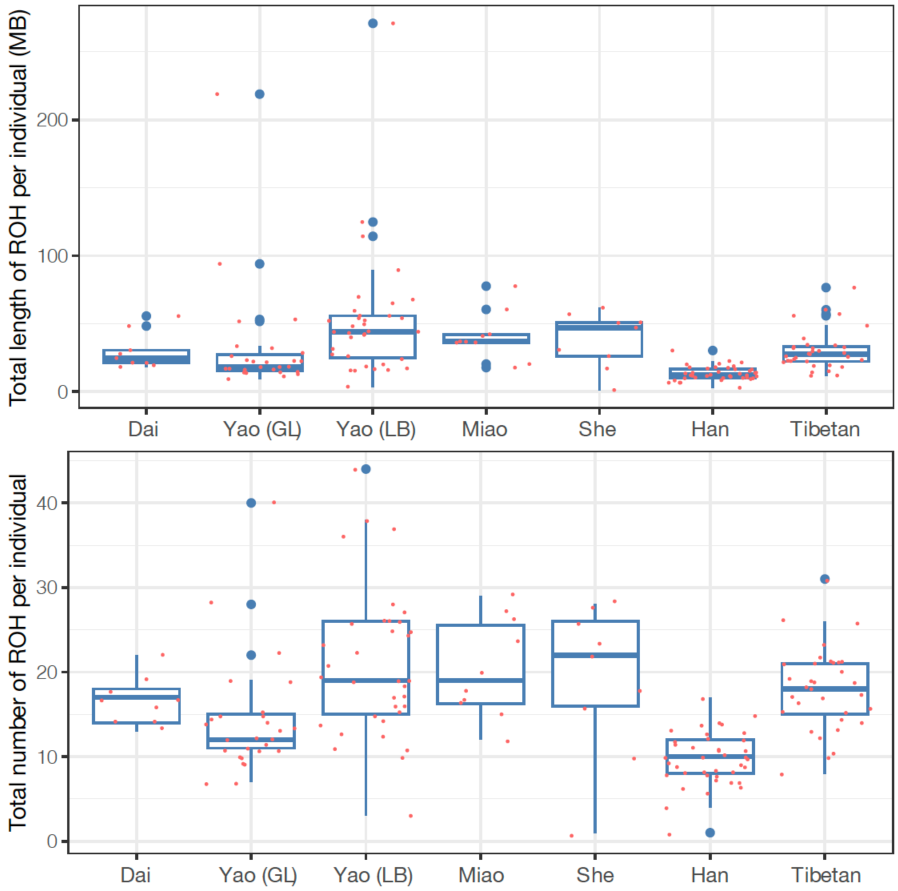

Fig.S12 | The length and quantity distribution of run of homozygosity (ROH) in different populations in the Panel 1 dataset.

We infer that the ROH of each population by the Plink software, and all parameters are default. Each point represents a sample. The sample size for each population is not less than 9. The length and number of ROH can help us infer the population history and the degree of inbreeding. Generally, more and longer ROH indicate the occurrence of inbreeding. In this result, the Yao population from the two sampling sites showed different ROH characteristics. Yao samples from Guilin (GL) have less and shorter ROH, which is also lower than Dai, She, Miao and Tibetan populations, and only higher than Han population. The length and number of ROH of Yao samples from Laibin (LB) are equivalent to that of She and Miao, higher than that of the Dai, Han and Tibetan populations. This difference is probably due to the city size of the sampling site. GL is the capital of Guangxi Province, and the population size is more than twice that of LB (census in 2020). In addition, we also found that a few Yao samples from GL and LB carried very many and very long ROH, which means that inbreeding still occurs recently. In addition, the She nationality is also very isolated compared with other groups.

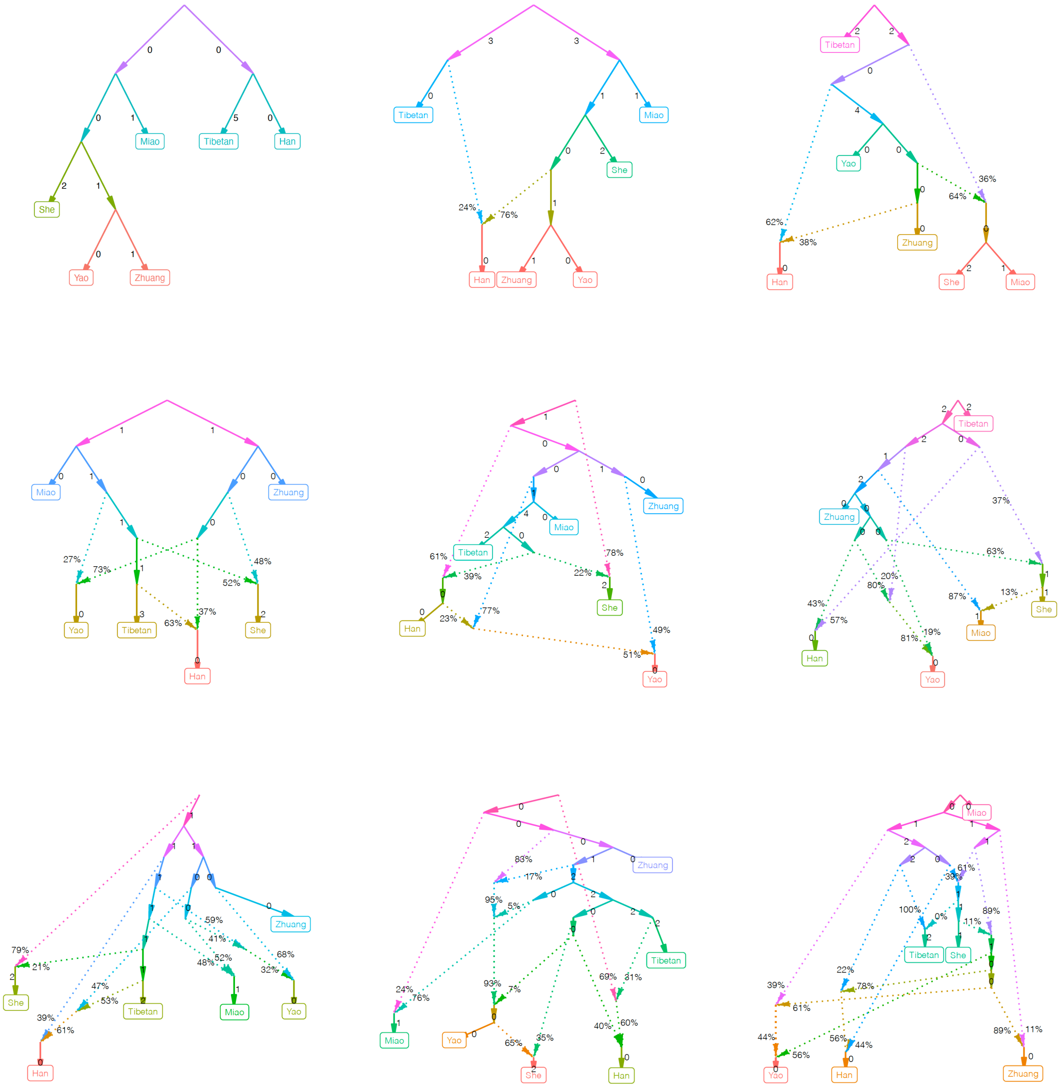

Fig.S13a | qpGraph models generated by automatic search.

numadmix from 0 to 8 and stop_gen = 100.

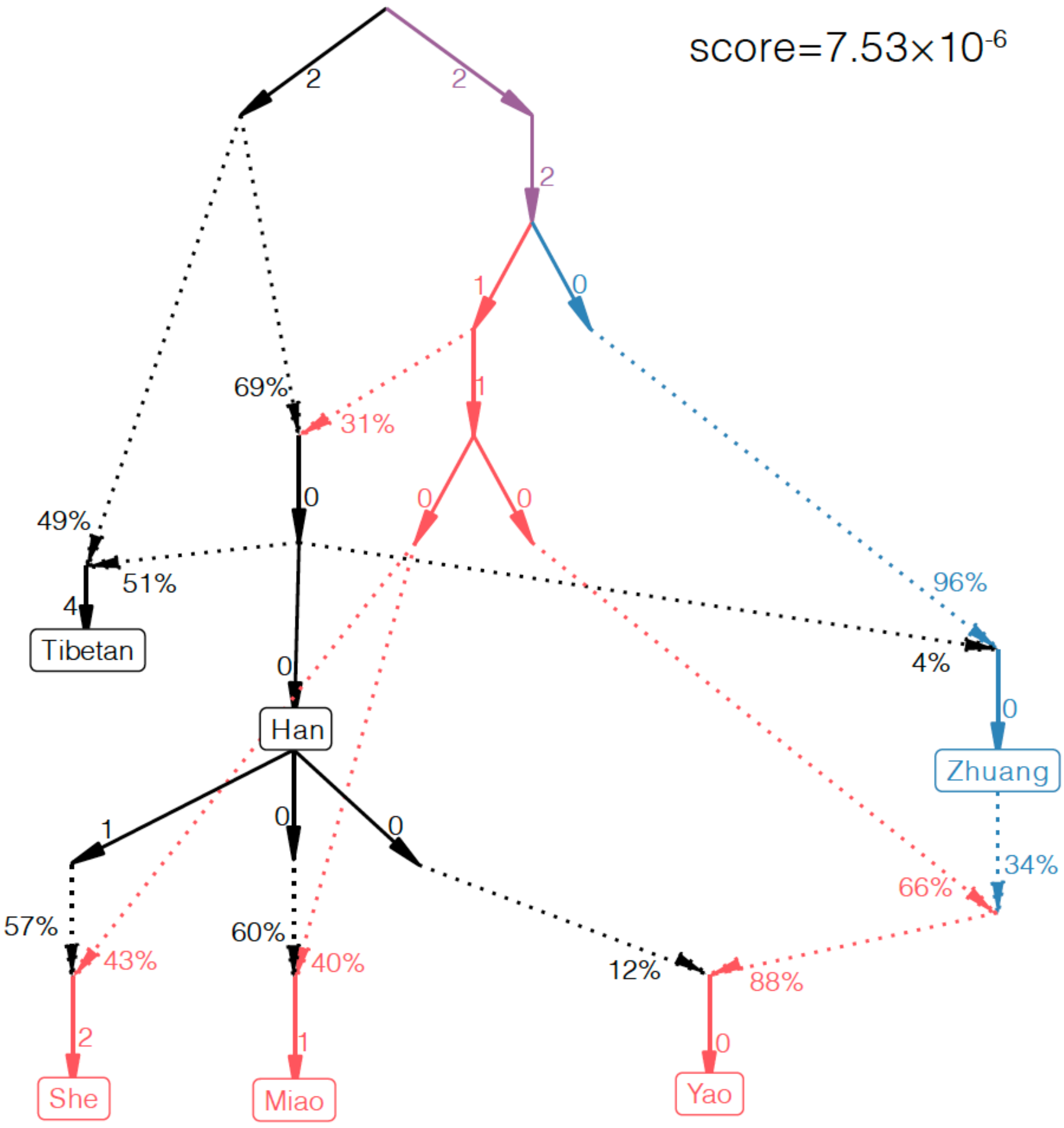

Fig.S13b | The qpGraph model of the basic skeleton designed by us.

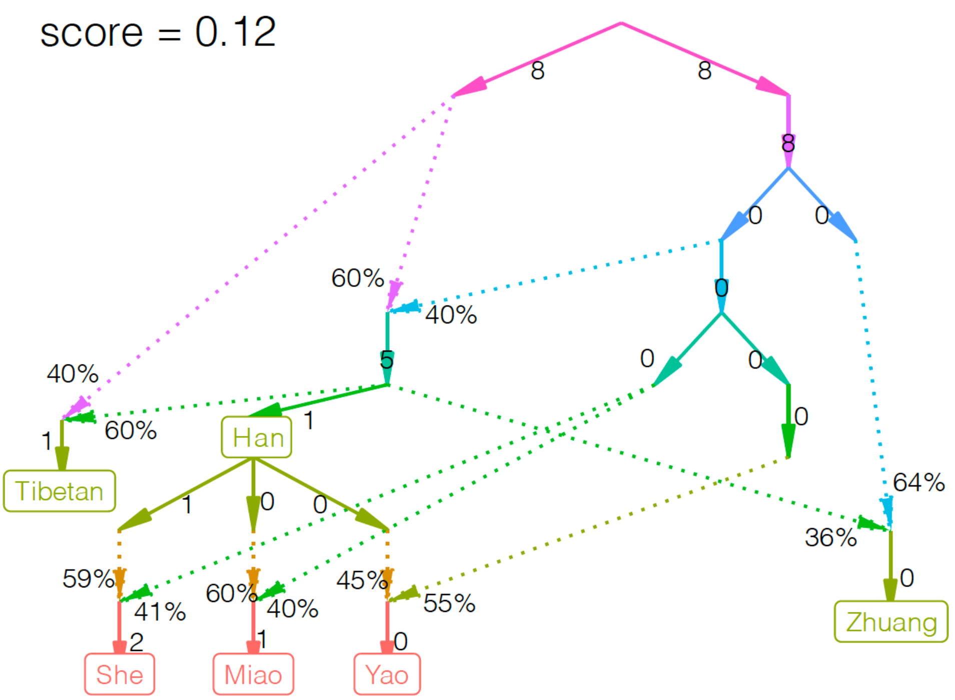

Fig.S13c | An alternative graph of Yao population not receiving the gene flow from the TK.

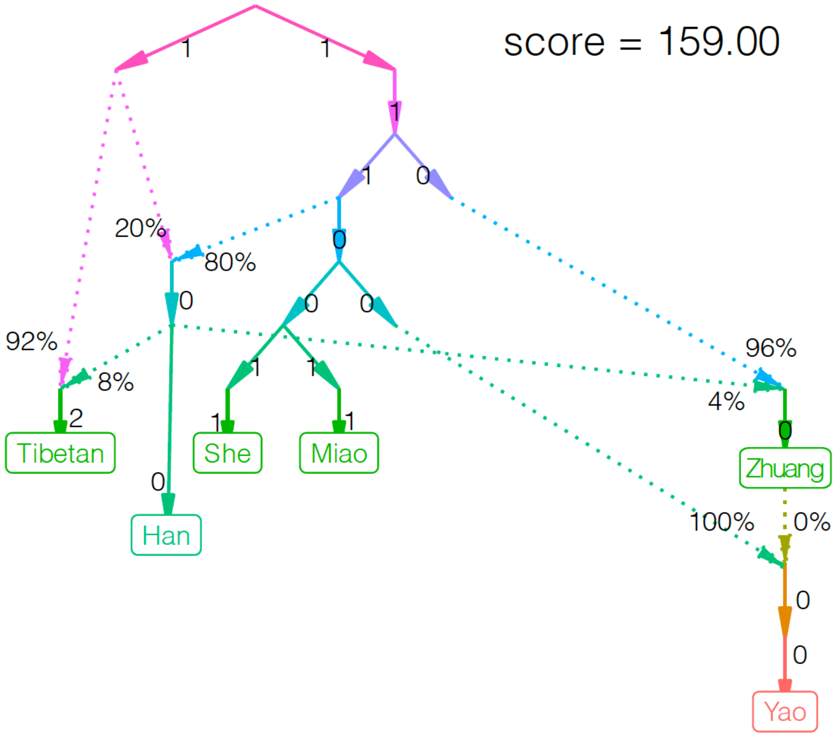

Fig.S13d | An alternative graph of the three HM populations not receiving the gene flow from the Han population.

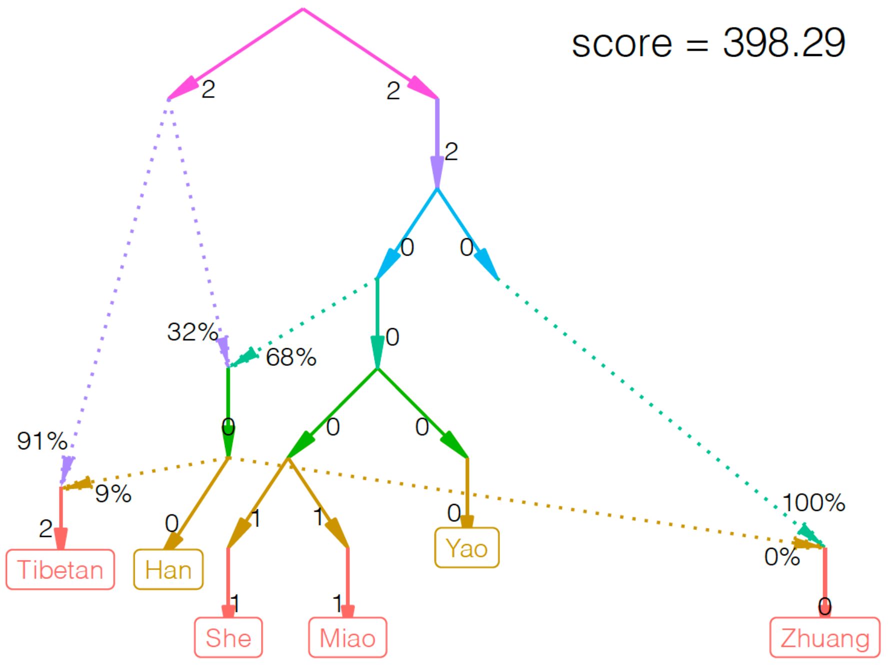

Fig.S13e | An alternative graph combining missing gene flow in Fig.S13d and Fig.S13c.

Fig.S13 | The admixture model of HM populations from qpGraph.

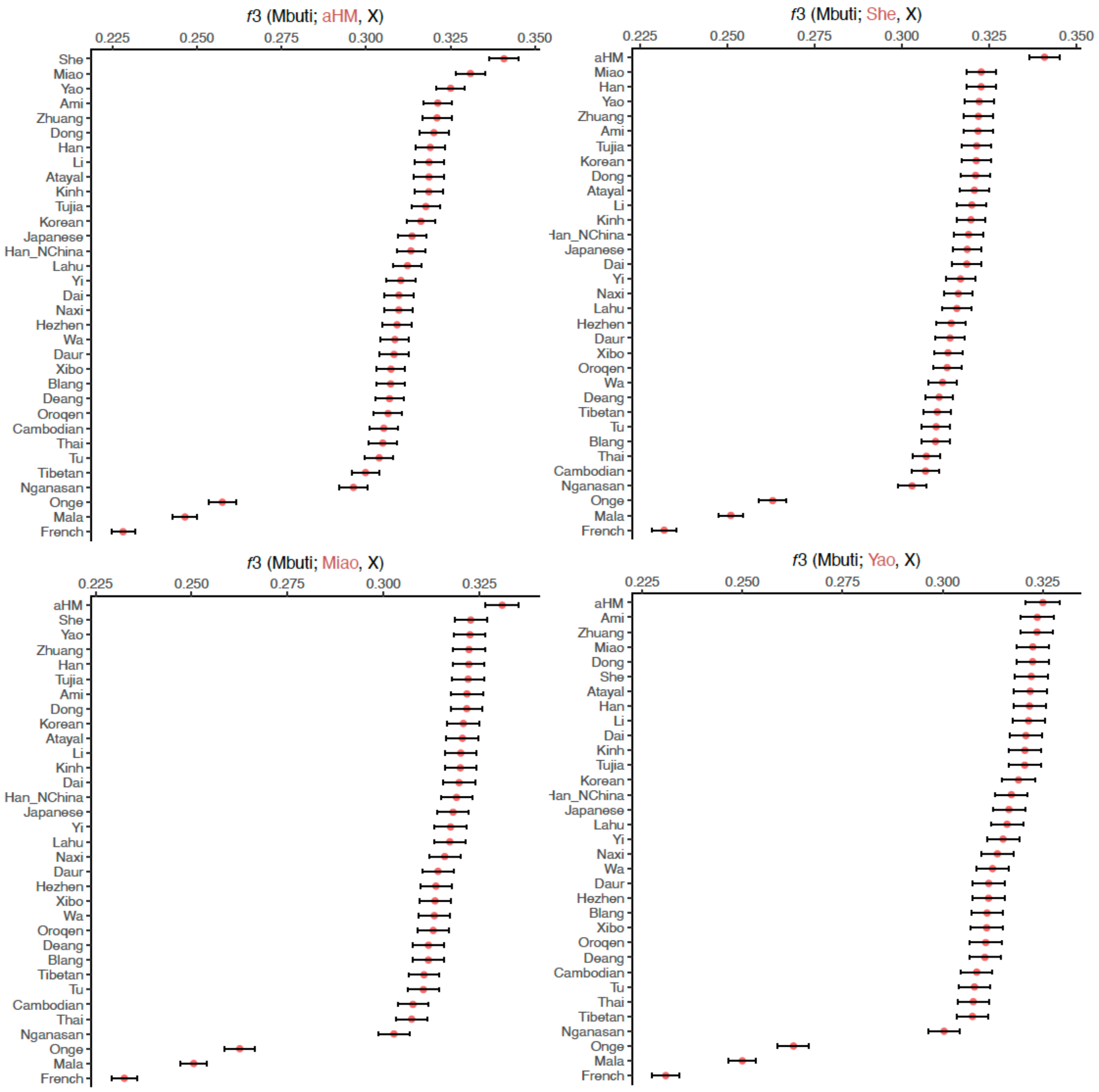

Fig.S14 | The present-day Hmong-Mien populations and the reconstructed ancestral population shared the most genetic drift

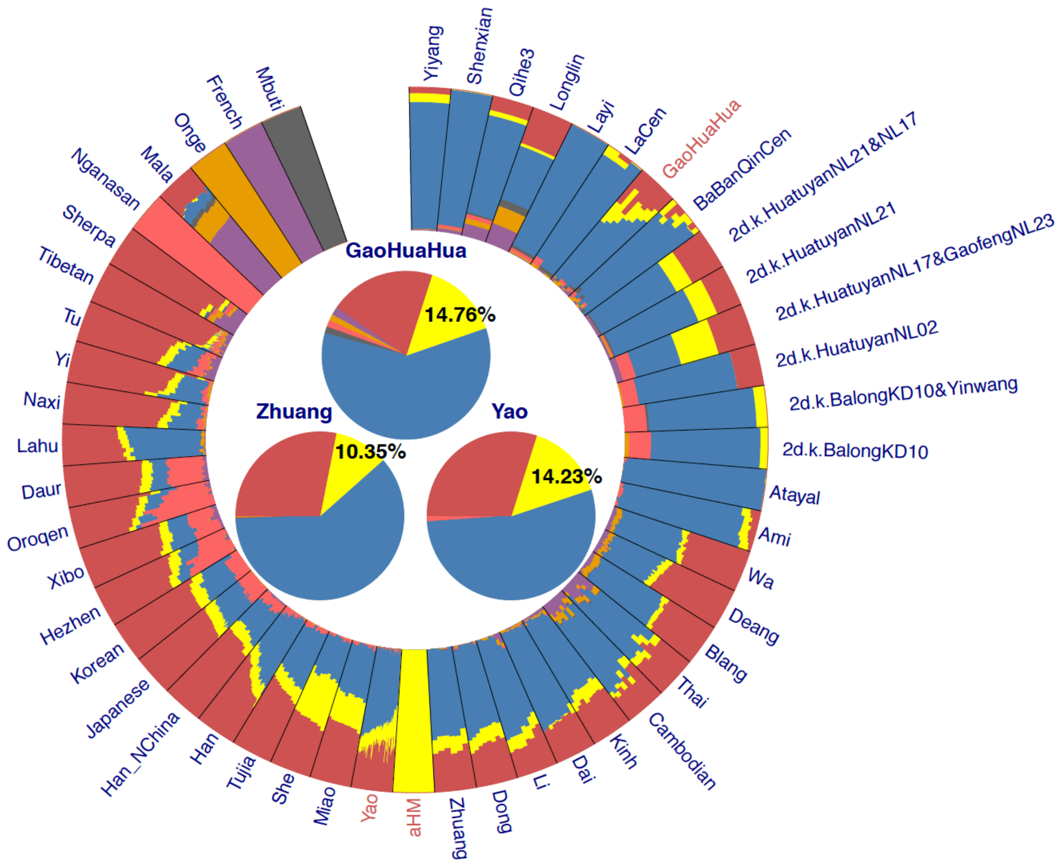

Fig.S15 | Inference of ancestral components of ancient DNA samples from Guangxi.

In a recent study on ancient DNA samples in Guangxi, they found that present-day HM speakers is closely related to an ancient population ("GaoHuaHua" cluster) about 500 years ago. Here we use this ancient population to test the representativeness of our reconstructed Hmong-Mien ancestral population. We integrated this batch of data and Panel 2 data set. After a loose quality control on the missing rate, we used ADMIXTURE to infer the ancestral source according to the number of ancestral components from 2 to 15. We selected the result that independent genetic components were separated in the north and south populations of East Asia, that is, K = 7.

Firstly, the genetic composition of the reconstructed Hmong-Mien ancestral population (aHM) is 14.23% in the Yao population (excluding the samples used to reconstruct the HM ancestral population). This proportion is higher than that of the Zhuang population (10.35%), although the ancestral composition of the Zhuang population is similar to that of the Yao population. In GaoHuaHua cluster, the proportion of aHM genetic components is 14.76%, which is closer and higher than that of today's Yao population. This result proves that our reconstructed Hmong-Mien ancestral population has a good representation of Hmong-Mien-specific genetic components.

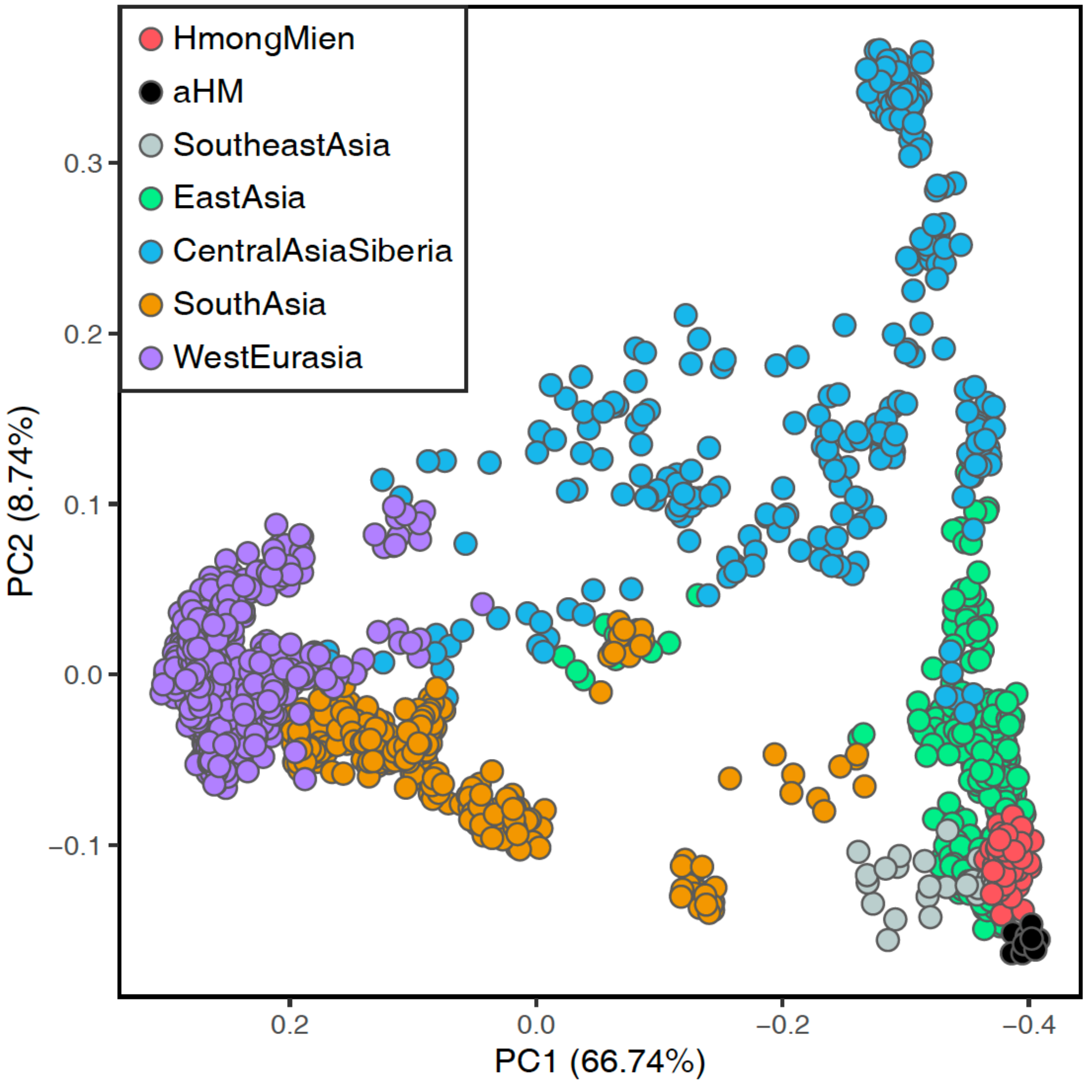

Fig.S16 | PCA of reconstructed HM ancestors in the context of Eurasia.

PCA was implemented for the reconstructed ancestral population together with the present-day Eurasian populations. The black dot represents our reconstructed HM ancestor population, and the red dot represents the present-day HM population. The genetic coordinates of HM ancestors are farther southeast than those of the present-day East-Asian population.

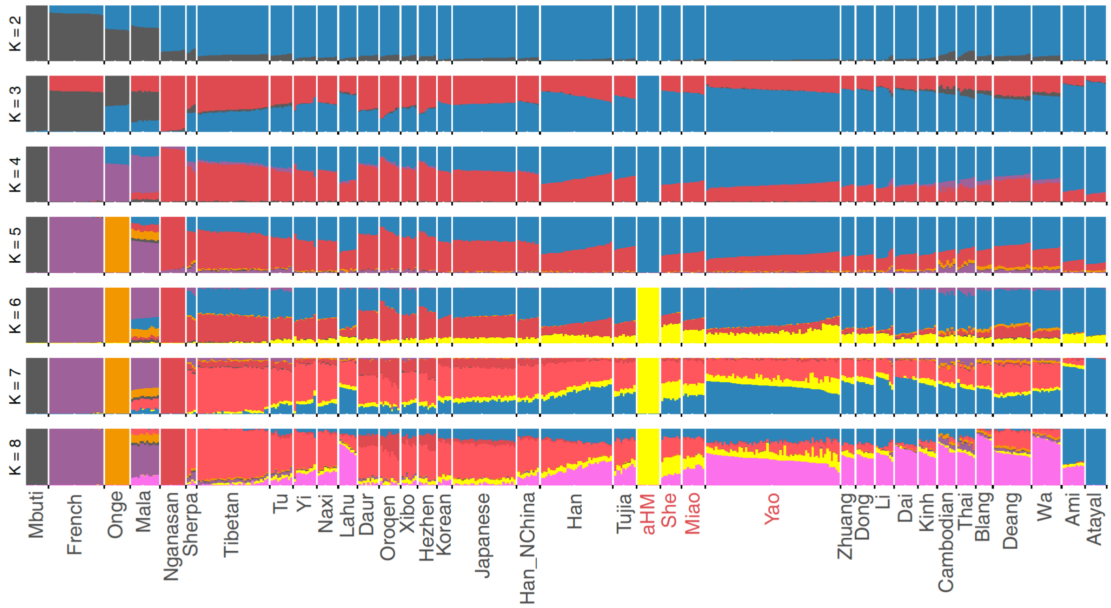

Fig.S17 | The reconstructed HM ancestral genome in admixture analysis.

We show the results of ADMIXTURE on the number of ancestral components K from 2 to 8. aHM is the HM ancestor we constructed. This ancestor of HM speakers always keeps a single genetic component. When k equals 3–5, this component represents a genetic component of Southern East Asia. When k is greater than 5, this component represents the ancestral component of the present-day HM population.

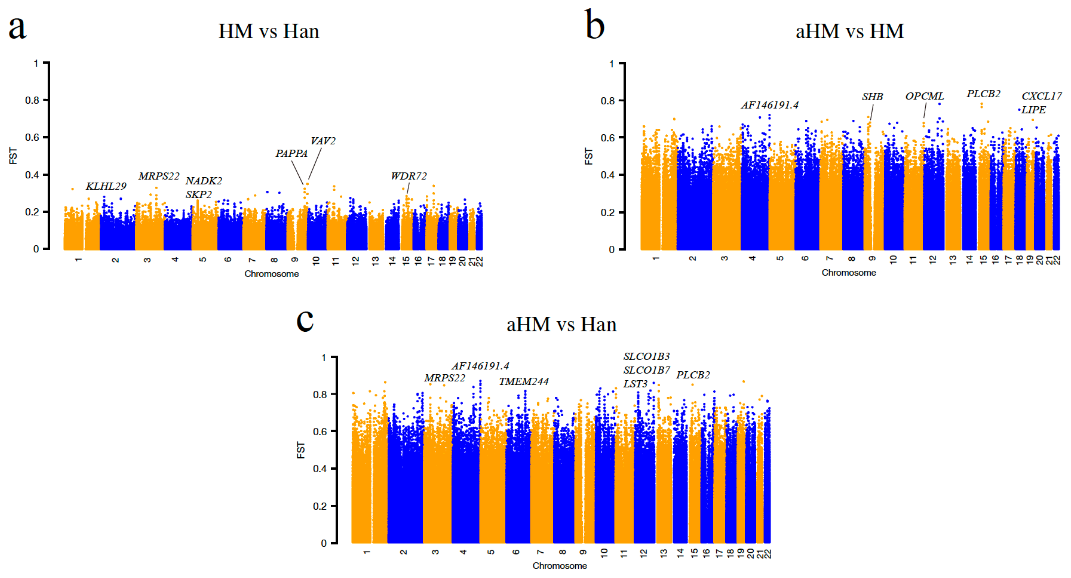

Fig.S18 | Manhattan plot of pairwise F_{ST} for aHM, HM and Han.

F_{ST} were calculated for each SNV among common SNVs for each population combination. Genes were marked in the figures for the most significant SNVs.

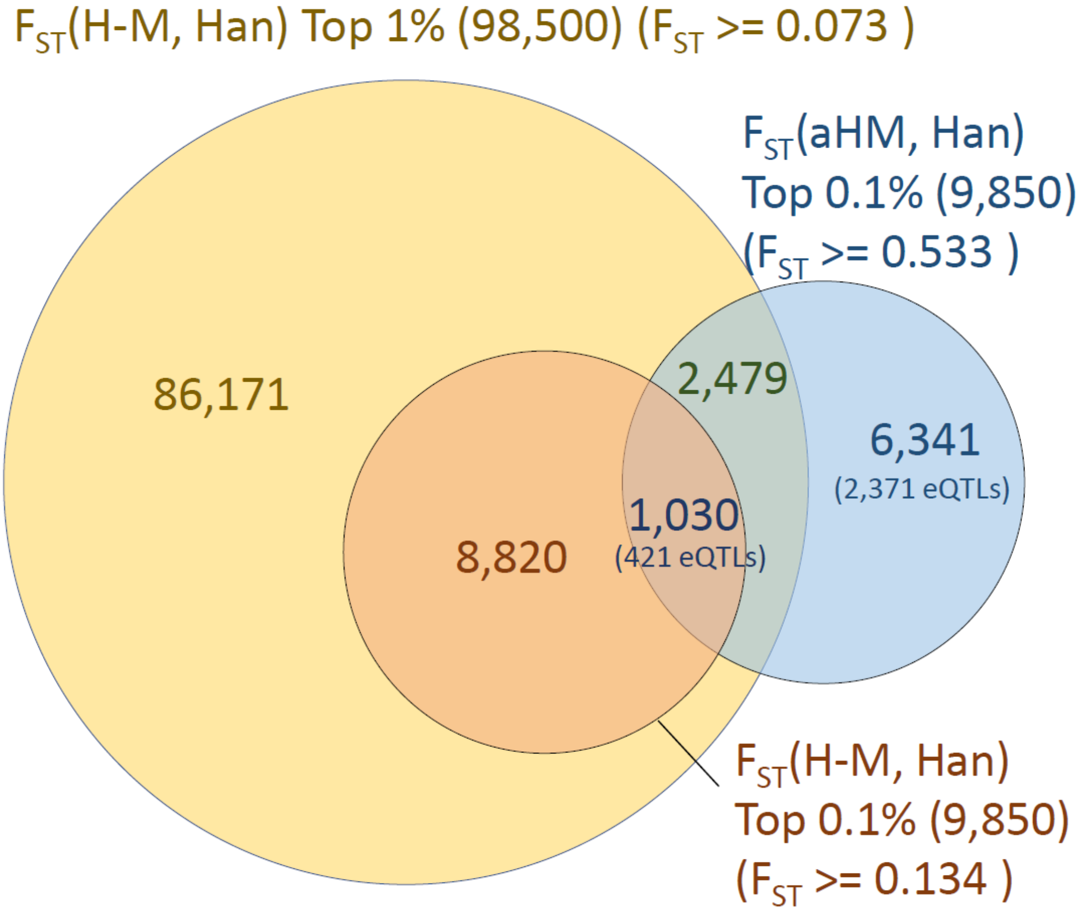

Fig.S19 | Additional selection signals were found in HM ancestral populations reconstructed by ancestral segments.

According to the regions covered by the reconstructed aHM genome, we calculated the F_{ST} for each variant. The red part is the top 0.1% part of F_{ST} between the modern H-M population (H-M) and Han. The blue part is the top 0.1% of F_{ST} between the reconstructed H-M ancestral population (aHM) and Han. The yellow part is the top 1% of F_{ST} between the modern H-M population (H-M) and Han.

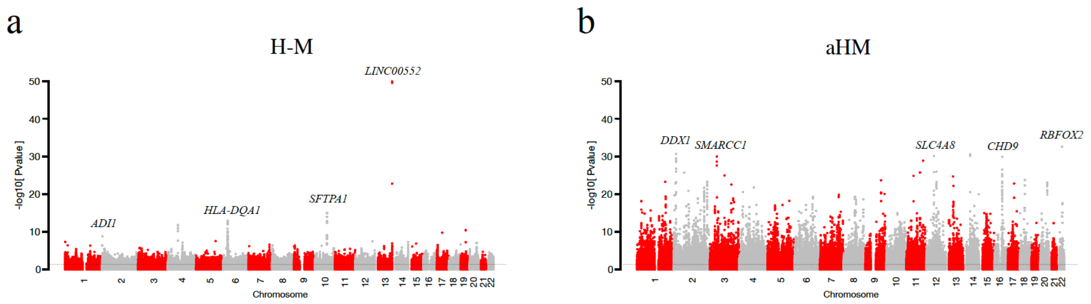

Fig.S20 | Manhattan plot using the P value of iHS for HM and aHM.

iHS were calculated and normalized by Selscan v1.2.0 with default parameters for each population using phased data. Take the negative logarithm with the base of ten for the P value and plot. (a) signals of Hmong-Mien speaking population. (b) signals of re-constructed Hmong-Mien ancestry. Genes were marked in the figures for the most significant SNVs.

For Hmong-Mien speaking population, the most significant signal gene is *LINC00552*, a lncRNA. Other genes such as *ADI1*, *HLA-DQA1* and *SFTPA1* are potentially affected by natural selection. For the reconstructed Hmong-Mien speaking ancestry population, the most significant signal gene is *RBFOX2*. Other genes such as *DDX1*, *SLC4A8*, *SMARCC1* and *CHD9* are identified with strong signal. Some of genes are still signals (|iHS|>2) in Hmong-Mien speaking population, but it is not prominent.

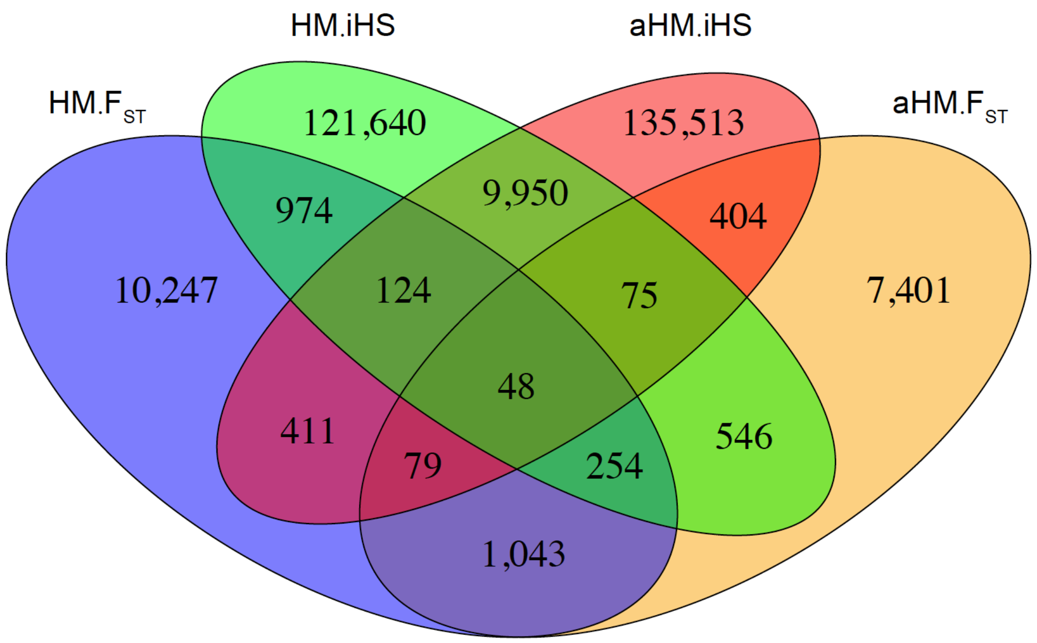

Fig.S21 | Venn plot of HM selection signals detected by different methods.

We used F_{ST} and iHS methods to detect selection signals in the HM (10 Yao, 10 Miao and 10 She) and aHM populations, and compared the differences in selection signals detected by different methods.

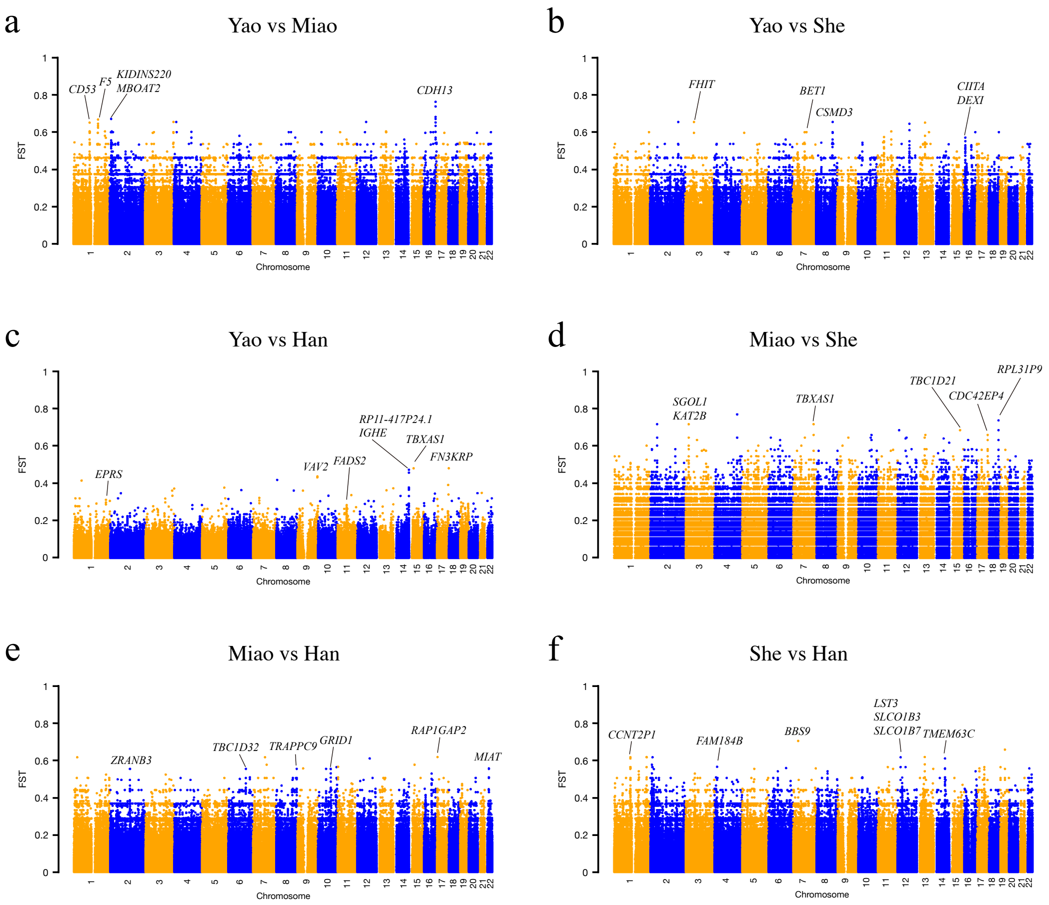

Fig.S22 | Manhattan plot of pairwise F_{ST} between HM and Han.

F_{ST} were calculated for each SNV among the whole genome in each population combination. Genes were marked in the figures for the most significant SNVs by VEP (GRCh37 ensemble92).

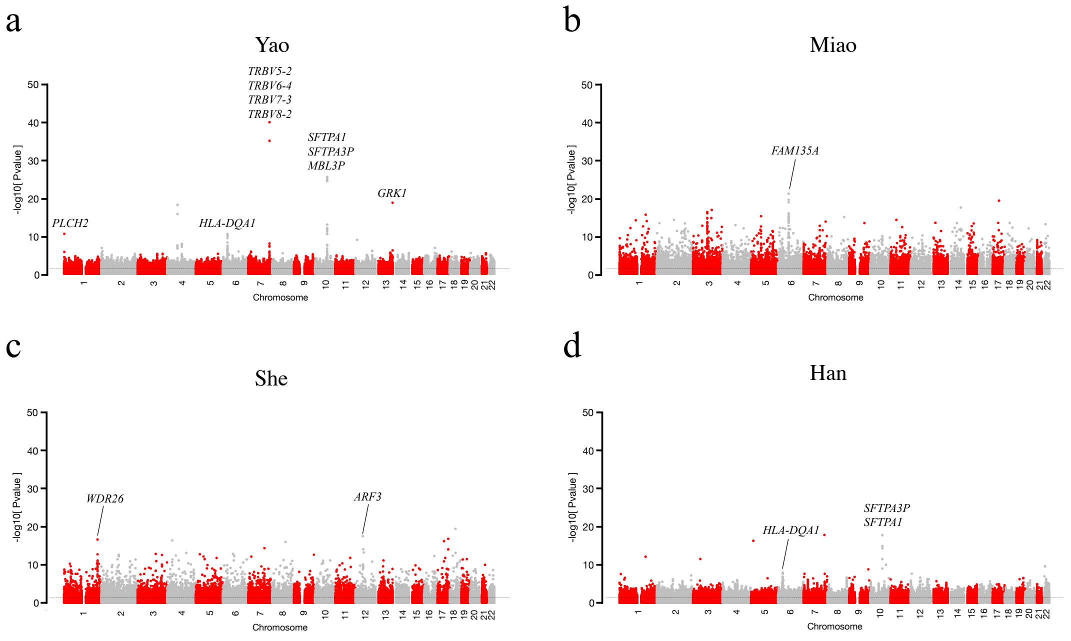

Fig.S23 | Manhattan plot using the P value of iHS for Yao, Miao, She and Han.

iHS were calculated and normalized by Selscan v1.2.0 with default parameters for each population using phased data. Take the negative logarithm with the base of ten for the P value and plot. Genes were marked in the figures for the most significant SNVs by VEP (GRCh37 ensemble92).

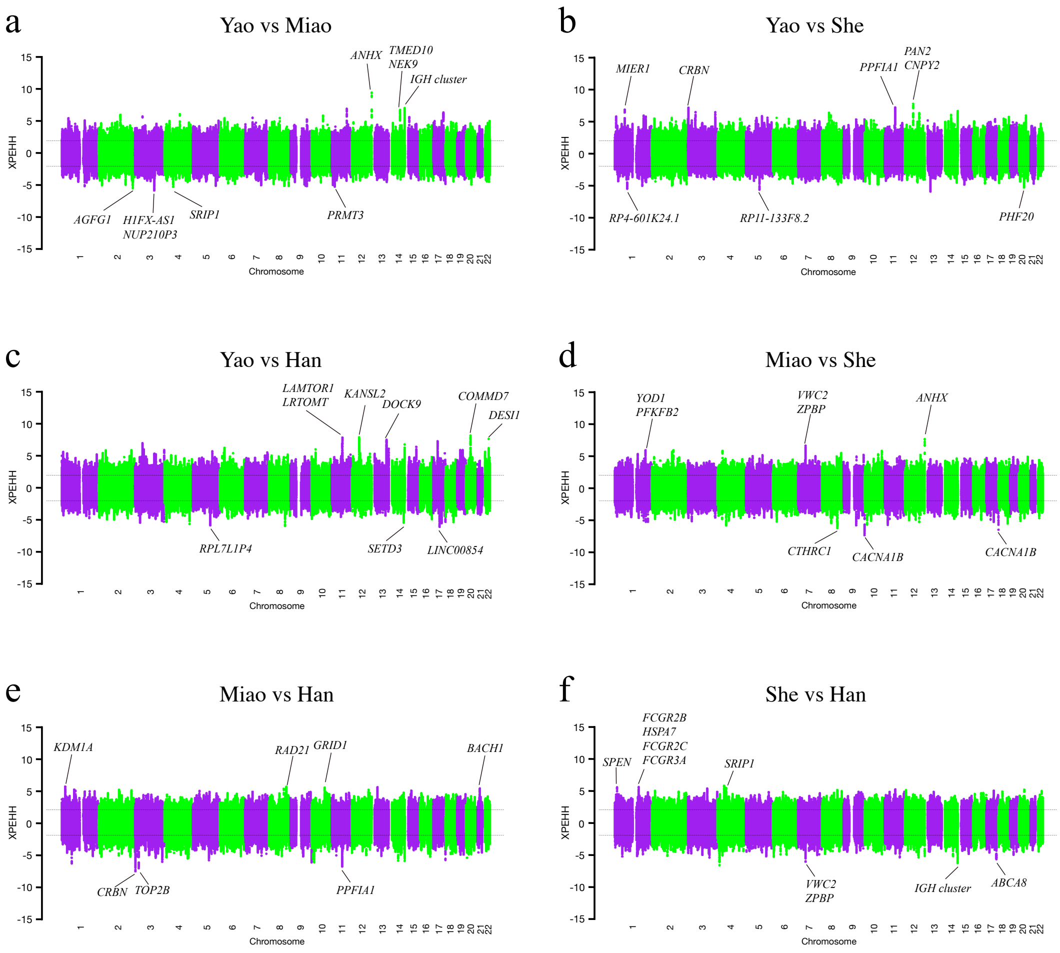

Fig.S24 | Manhattan plot of pairwise XPEHH for Yao, Miao, She and Han.

XPEHH were calculated and normalized by Selscan v1.2.0 with default parameters for each population combination using phased data. Positive values suggested potential selection signals in the first population, and negative values in the second population. Genes were marked in the figures for the most significant SNVs by VEP (GRCh37 ensemble92).

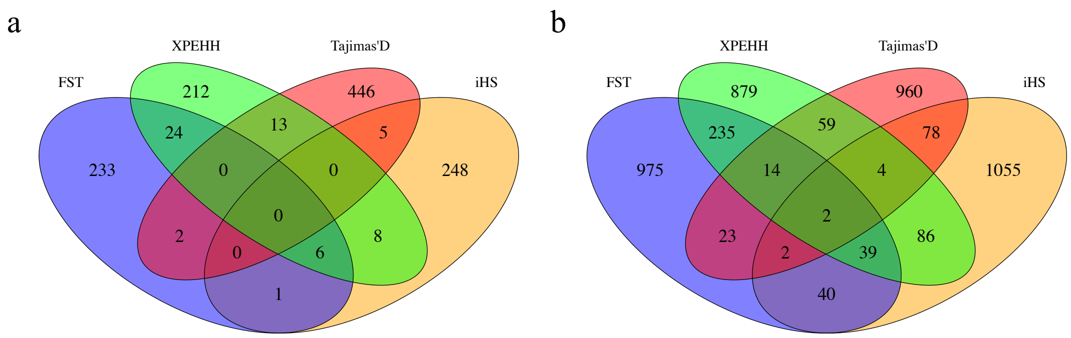

Fig.S25 | Venn plot of shared signal segments identified by 4 methods in Yao.

Whole genome was divided into 100kb segments. For methods between populations, Yao and Han were selected as concerned populations. Average of top 3 highest F_{ST}, iHS with maximum absolute value and the most extreme XPEHH value suggesting Yao under selection were regarded as the characteristic values of one segment. Further segments were sorted by characteristic values separately. (a) top 1% significant segments of F_{ST}, iHS and XPEHH and segments with origin P value less than 0.01 for Tajima' s D. (b) top 5% significant segments of F_{ST}, iHS and XPEHH and segments with P value less than 0.05 for Tajima' s D.

For the results, most of signal segments identified in each method were unique for its own, only a small number of signals were shared with other methods. Thus, we found 6 sharing segments in 3 most commonly used method (F_{ST}, iHS and XPEHH). These 6 shared signal segments included chr13:99400001-99800000 (Two protein coding genes: *SLC15A1*, plays an important role in the uptake and digestion of dietary proteins; *DOCK9,* associated with irregular astigmatism and corneal ectasia.), chr14:106000001-106100000 (Two protein coding genes: *IGHA2* and *IGHG4*, both of them involve immunoglobulin heavy chains.) and chr19:57500001-57600000 (No protein coding gene. Only a pseudogene *RPL7AP69*.).

Furthermore, using more relaxed threshold can help us find two signal segments sharing in all four methods including chr1:161500001-161600000 (Two protein coding genes: *FCGR3A* and *FCGR3B*, both of them involve wow affinity immunoglobulin gamma Fc region receptor) and chr6:32500001-32600000 (Two protein coding genes: *HLA-DRB1* and *HLA-DQA1*, both of them involve HLA class II histocompatibility antigen.).

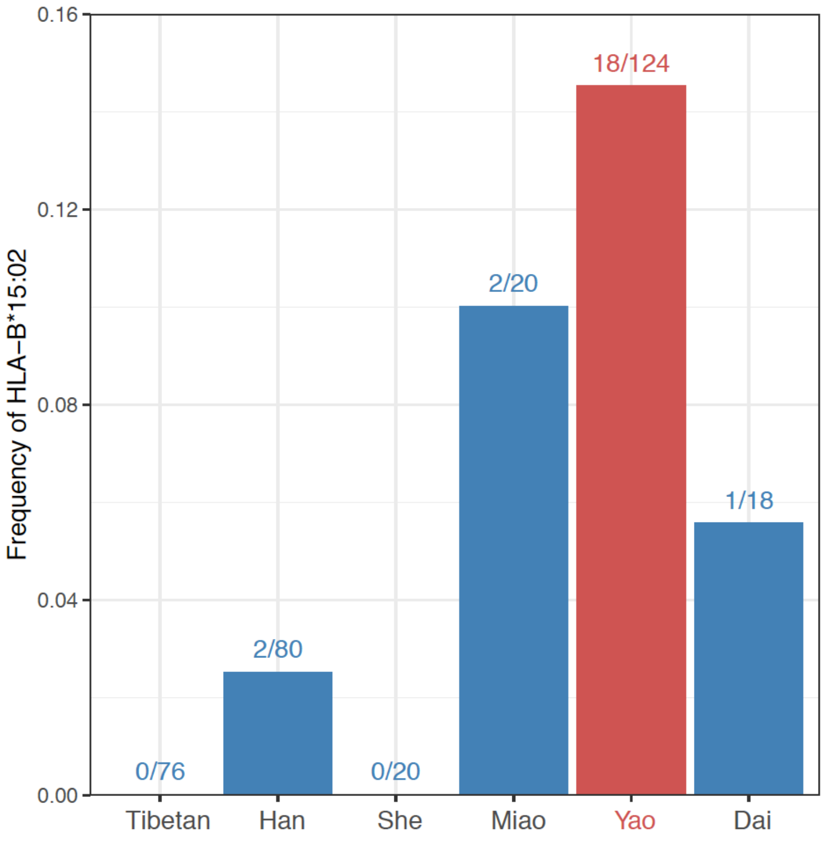

Fig.S26 | The frequency of HLA-B*1502 allele in East Asia populations.

We inferred the HLA type of each sample based on the Panel 1 dataset. The HLA-B*15:02 allele was reported to be strongly associated with severe skin allergic reaction caused by carbamazepine, including Stevens Johnson syndrome (SJS) and toxic epidermal necrolysis (TEN). The HLA-B*15:02 alleles exist almost exclusively in populations of Asian descent. Therefore, with the support of large sample size Yao deep sequencing data, we calculated the frequency of the HLA-B*15:02 allele.

From the results, the Yao population carries 14.52% of the HLA-B*15:02 allele, which is much higher than that of Han, Tibetan and Dai population. The Miao population carry 10.00% of the HLA-B*15:02 allele, second only to the Yao population. However, No the HLA-B*15:02 allele was observed in the She population. About 5.56% of the Dai population carry the HLA-B*15:02 allele, but considering the low resolution caused by small sample size, we use the CDX population from KGP for further verification. 6.99% (13/186) of Dai samples from KGP carry the HLA-B*15:02 allele, which means that the frequency in our data is basically stable. The high frequency of the HLA-B*15:02 allele observed in Yao population provides a certain guiding role for drug use in the future. In addition, the high frequency in Yao population may also be the influence of the natural selection, which needs further research.

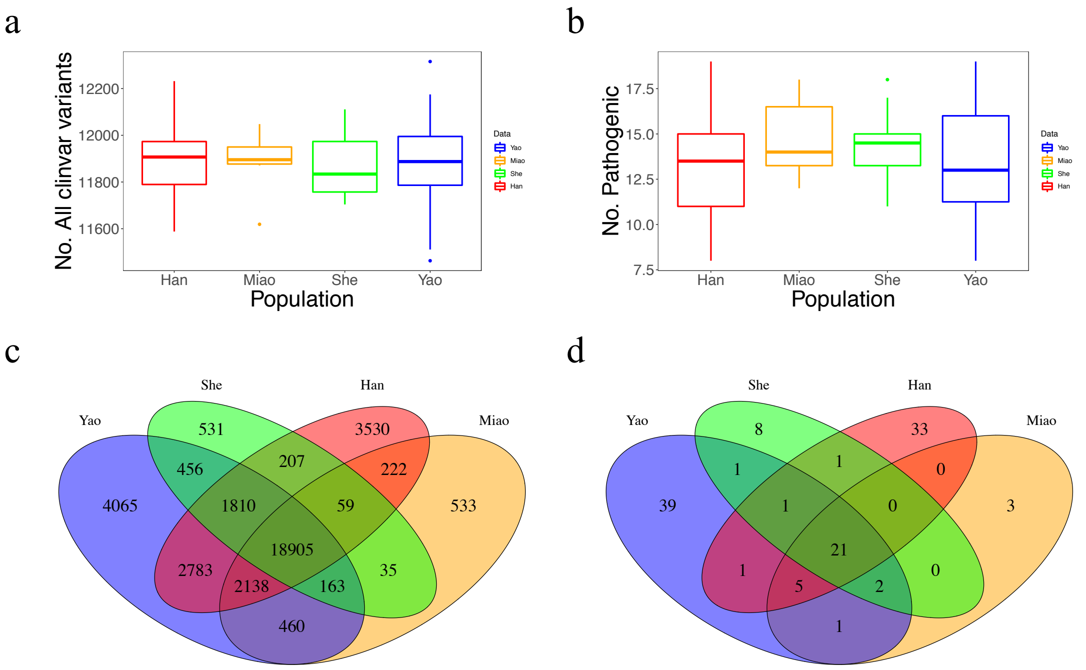

Fig.S27 | Clinically relevant SNVs carried by the population and sharing among populations.

Genome wide SNVs of Yao, Miao, She and Han were annotated by Clinvar database (GRCh37 20210302). (a) boxplot of the number of all Clinvar variants in each population. (b) boxplot of the number of pathogenic and likely pathogenic variants in each population. (c) venn plot of all Clinvar variants among four populations. (d) venn plot of only pathogenic variants among four populations. To compare differences of carrying clinical variants between Yao and other populations, we performed two side Wilcoxon rank sum test. As a result, Yao did not show significant difference from other populations about the number of all Clinvar variants (Yao-Han: Pvalue=0.7708; Yao-Miao: Pvalue=0.7508; Yao-She: Pvalue=0.5307) and pathogenic including likely pathogenic variants (Yao-Han: Pvalue=0.4988; Yao-Miao: Pvalue=0.2315; Yao-She: Pvalue=0.3055).

In total, four populations carried 35,497 clinical SNVs, more than half of which (18,905) were shared in all of the four populations. Yao and Han had much more clinical SNVs as well as pathogenic SNVs than Miao and She for their large sample size. As for pathogenic SNVs, we found two variants only shared in three Hmong-Mien speaking populations. One was rs11594656 (chr10:6122009-A; intergenic variant) related to insulin-dependent diabetes mellitus. The other was rs72474224 (chr13:20763612-T; *GJB2*; missense variant) related to hearing impairment.

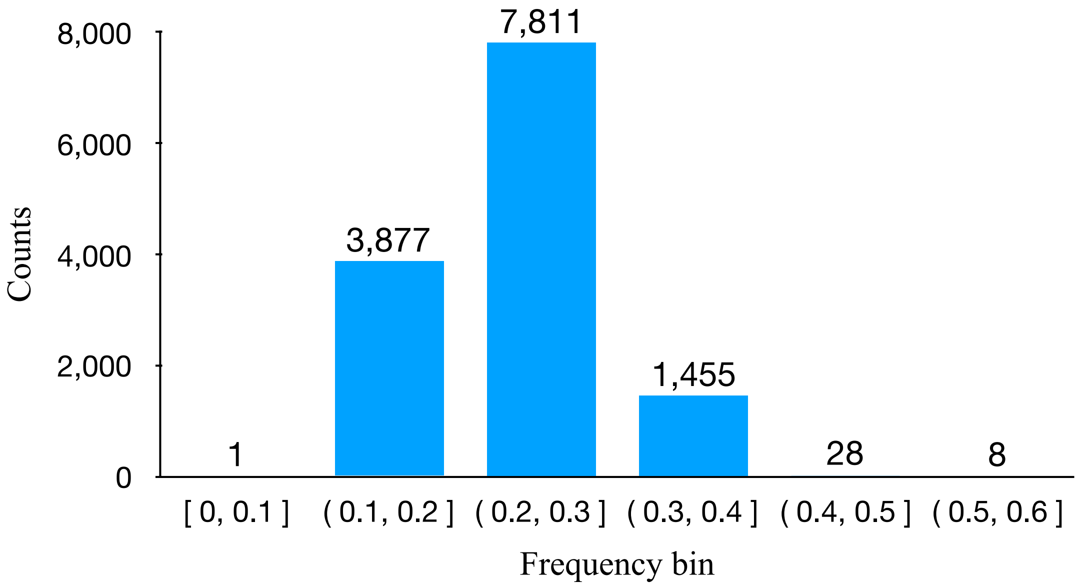

Fig.S28 | The distribution of frequency differences of HDVs between Yao and Han.

We calculated pairwise F_{ST} for each SNV in the whole genome between Yao and Han population Top 0.1% highest F_{ST} were identified as highly differentiated variants (HDVs). Finally, 13,180 HDVs were divided into 6 frequency bins based on frequency differences between Yao and Han. More than half of the HDVs had a frequency difference between 0.2 and 0.3. 8 HDVs had a frequency difference more than 0.5 (chr14:106249173, chr14:106249198, chr14:106250353, chr14:106259853, chr14:106261106, chr14:106265167, chr14:106267941, chr14:106268138). All these 8 SNVs are located in intergenic region.
